# Spatial modeling of the membrane-cytosolic interface in protein kinase signal transduction

**DOI:** 10.1371/journal.pcbi.1006075

**Published:** 2018-04-09

**Authors:** Wolfgang Giese, Gregor Milicic, Andreas Schröder, Edda Klipp

**Affiliations:** 1 Mathematical Cell Physiology, Max Delbrück Center for Molecular Medicine, Berlin, Germany; 2 Department of Mathematics, University of Salzburg, Salzburg, Austria; 3 Theoretische Biophysik, Humboldt-Universität zu Berlin, Berlin, Germany; Chalmers University of Technology, SWEDEN

## Abstract

The spatial architecture of signaling pathways and the interaction with cell size and morphology are complex, but little understood. With the advances of single cell imaging and single cell biology, it becomes crucial to understand intracellular processes in time and space. Activation of cell surface receptors often triggers a signaling cascade including the activation of membrane-attached and cytosolic signaling components, which eventually transmit the signal to the cell nucleus. Signaling proteins can form steep gradients in the cytosol, which cause strong cell size dependence. We show that the kinetics at the membrane-cytosolic interface and the ratio of cell membrane area to the enclosed cytosolic volume change the behavior of signaling cascades significantly. We suggest an estimate of average concentration for arbitrary cell shapes depending on the cell volume and cell surface area. The normalized variance, known from image analysis, is suggested as an alternative measure to quantify the deviation from the average concentration. A mathematical analysis of signal transduction in time and space is presented, providing analytical solutions for different spatial arrangements of linear signaling cascades. Quantification of signaling time scales reveals that signal propagation is faster at the membrane than at the nucleus, while this time difference decreases with the number of signaling components in the cytosol. Our investigations are complemented by numerical simulations of non-linear cascades with feedback and asymmetric cell shapes. We conclude that intracellular signal propagation is highly dependent on cell geometry and, thereby, conveys information on cell size and shape to the nucleus.

## Author summary

Frequently, cells detect signals at their surface, which are transmitted to the nucleus. The influence of cell shape and size is often neglected and cells are regarded as well-mixed compartments. However, the advance of modern microscopy has unraveled heterogeneous distribution of signaling molecules in the cell and variations depending on cell shape, size and organelle arrangement. Understanding spatial signaling usually involves solving mathematical equations in space and time including approximations or sophisticated numerical methods. We provide exact analytical solutions for the steady state of two different spatial arrangements of a generic linear signaling cascade model. Furthermore, the dynamic process is investigated using advanced computational techniques. Implications are drawn on single-cell variation in signal transduction and on spatial regulation by cell size and shape.

## Introduction

Cells need to respond to a large variety of external stimuli such as environmental changes or extracellular communication signals. Signals transmitted from cell surface receptors to target genes in the nucleus are frequently transduced by cascades of covalent protein modifications. These modifications consist of inter-convertible protein forms, for instance, a phosphorylated and an unphosphorylated protein. Signaling cascades occur in many different variations including mitogen-activated protein-kinase (MAPK) cascades and small GTPase cascades.

**Fig 1:**
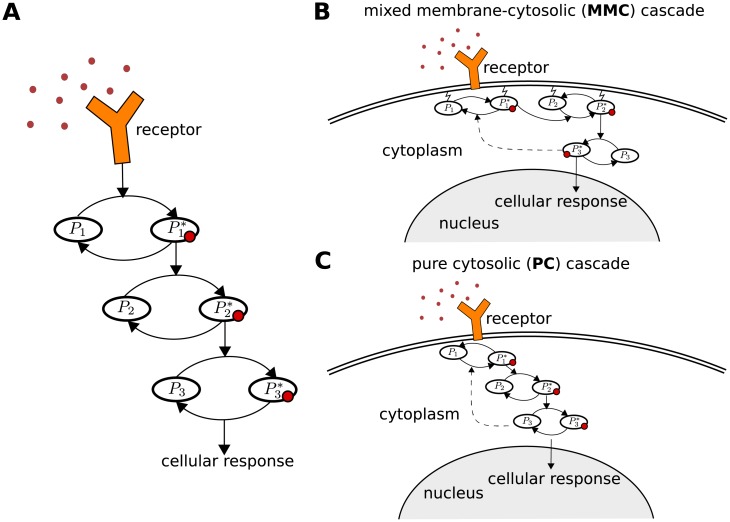
Spatial organization of signaling cascades. (**A**) Sketch of the classical temporal signal transduction model. Extension of this model into three-dimensional space naturally results in a variety of different spatial motifs. (**B**) The signal is first processed by signaling components tethered to the membrane, and then transduced at membrane-cytosolic interface into the cytosol. (**C**) The signaling components are directly activated at the membrane-cytosolic interface and diffuse through the cytosol. Note that diffusion coefficients for lateral diffusion along the membrane are much lower than in the cytosol.

Signal transduction mechanisms carried out by networks of protein-protein interactions are highly modular and regulatory behavior arises from relatively simple modifications [1]. The spatial arrangement of signaling cascades varies in different biological systems. We focus on the localization of signaling components, which can be tethered to the cell-membrane or freely diffuse in the cytosol. Tethering of signaling molecules to the cell-membrane can be mediated by lipidation modifications [2–6], co-localization by membrane-bound scaffolds [7] or membrane anchoring proteins [8]. Frequently, the first steps of signal transduction occur at the membrane and are then continued into the cytosol. We investigate linear signaling cascades with different realizations of spatial arrangements of signaling components as shown in Fig 1. Here, we focus on the membrane-cytosolic interface, which is included in the signaling motif shown in Fig 1(B) and 1(C).

In many experimental and theoretical studies on signaling cascades, the cell is regarded as a number of well-mixed compartments with no variation in size, shape or organelle location. Attempts of a quantitative description of signaling cascades with a focus on temporal aspects have been made in [9–12]. However, the spatial description of signaling processes has received less attention despite its relevance in understanding cell morphology and growth regulation in time and space [13]. Examples of spatial effects on the length scale of single cells range from the yeast mating process [14, 15] to the propagation of spatial information in hippocampal neurons which is controlled by cell shape and *vice versa* [16, 17].

Since the cytosol scales with cell volume and the cell membrane with the cell surface area, reactions on the membrane and in the cytosol scale with the cell-surface to cell-volume ratio. For instance, we obtain an area/volume ratio of ∝ 3/*R*_cell_ for a spherical cell geometry, where *R*_cell_ is the cell radius. We will show that this scaling affects the global phosphorylation rate of signaling proteins that diffuse in the cytoplasmic volume, which depends on cell size. While cytosolic gradients naturally occur from the membrane to the nucleus, membrane-bound components can only form gradients along the membrane, which changes the response to heterogeneous signals. Furthermore, the diffusion on the membrane is much slower for membrane-bound components than for cytosolic components [18]. Both of these factors are expected to largely change signal transduction properties of the pathway.

An analysis and comparison of spatial signal transduction motifs in response to spatially homogeneous and heterogeneous signals is presented in this study. The natural extension of widespread used ordinary differential equations are bulk-surface partial differential equations [19, 20]. Here, *bulk* refers to the cellular compartments that are represented as a volume such as the cytoplasm or the nucleus, while *surface* refers to all cellular structures that are represented as an area such as the cellular or nuclear membrane. Since their introduction to cell signaling systems [21], bulk-surface partial differential equations have been successfully employed in several models for cell polarization [18, 22–24]. However, membrane-cytosolic interfaces at different stages of a signaling cascade have not yet been investigated.

We start with an analysis of two different motifs with simplified linear kinetics, which allows to develop exact analytical solutions of the steady state. Both motifs differ in their cell size dependence and we show further that their behavior can be drastically different from the assumption of well-mixed compartments. The time-scaling of signal transduction is investigated using the method of local accumulation times [25]. We continue by investigating the response and sensitivity to spatially heterogeneous signals such as signaling gradients for symmetrical and asymmetrical cell shapes. In the last section, we proceed with numerical investigations of systems with negative feedbacks which lead to cell-size dependent oscillations. A Fourier analysis in time is used to provide insight into the dependency of oscillation frequency and amplitude on cell size. Depending on the spatial motif, cell size limits for the extinction of oscillatory behavior are obtained.

We start with a linear signaling cascade with different localizations of the membrane-cytosolic interface as shown in Fig 1. We employ a simple cascade model from [9], in which stimulation of a receptor leads to the consecutive activation of several down-stream protein kinases. This model is extended into space in the following. We assume a linear cascade with *N* components, where the first *M* < *N* components are localized at the membrane while the remaining *N* − *M* components are assumed to freely diffuse in the cytosol. The equations for the membrane-bound components read

$$\begin{array}{ccc} \frac{\partial P_{n}}{\partial t} & =D_{\text{mem}}\Delta_{\Gamma }P_{n}+v_{n}^{a}-v_{n}^{d} & \;\text{on}\;\text{the}\;\text{cell}\;\text{membrane},\;\text{for}\;n=1,\ldots ,M. \end{array}$$ (1)

Here, $P_{1}\left(\vec{x},t\right),\ldots ,P_{M}\left(\vec{x},t\right)$ are the local concentrations of signaling molecules on the cell membrane. The activation rate of the first signaling component $v_{1}^{a}$ is assumed to be dependent on the input signal, which is denoted by $P_{0}\left(\vec{x},t\right)$. The input signal on the cell surface can be a trigger on the cell membrane or arise from an extracellular signal. All of these species are functions of space and time, where $\vec{x}$ is a point on the membrane and *t* is the time. Diffusion along the cell membrane, which is assumed to be a two-dimensional curved surface in three-dimensional space, is described by the Laplace-Beltrami operator Δ_Γ_ and the diffusion coefficient *D*_mem_. Since the membrane is a surface in three-dimensional space with negligible thickness, the natural unit for concentrations of the cell membrane-bound species *P*_*n*_ (*n* = 1, …, *M*), is molecules per area. Molecular concentrations of signaling molecules are frequently provided in nanomolar or micromolar (nM or *μ*M). For convenience, we therefore use the units nanomolar or micromolar times micrometer (*n*M*μm* or *μ*M*μm*) for the membrane-bound signaling molecules. Note that 1 *μ*M*μ*m ≈ 602 molec/*μm*^2^.

The phosphorylation rates $v_{n}^{a}$ as well as the dephosphorylation rates $v_{n}^{d}$ have units molecules per area and time. If the input signal is homogeneous in space, meaning $P_{0}\left(\vec{x},t\right)=P_{0}\left(t\right)$, all spatial fluxes *D*_mem_∇_Γ_*P*_*n*_ are zero and the equation system for the membrane-bound species can be described by an equivalent system of ordinary differential equations (S1 Appendix). In contrast to the membrane-bound signaling components *P*_1_, …, *P*_*M*_, the signaling component *P*_*M*+1_ can freely diffuse in the cytosol. For the modeling of the membrane-cytosolic interface, we need to include diffusion in the cytosol and reactions on its boundaries, which are the membranes. These processes are modeled by a reaction-diffusion equation

$$\begin{array}{ccc} \frac{\partial P_{M+1}}{\partial t} & =D_{\text{cyt}}\Delta P_{M+1}-v_{M+1}^{d} & \;\text{in}\;\text{the}\;\text{cytosol,} \end{array}$$ (2)

with the boundary condition

$$\begin{array}{ccc} -D_{\text{cyt}}\nabla P_{M+1}\cdot \vec{n} & =v_{M+1}^{a}-v^{i} & \;\text{on}\;\text{the}\;\text{cell}\;\text{membrane.} \end{array}$$ (3)

Since *P*_*M*+1_ is activated by the upstream component *P*_*M*_, which is tethered to the membrane, there is a phosphorylation reaction only at the cell membrane but not in the interior of the cytosol. This reaction is, therefore, modeled as a boundary condition. The reactions at the membrane-cytosolic interface are described by the phosphorylation rate $v_{M+1}^{a}$ and the inactivation rate *v*^*i*^, both with units molecules per area and time. The species *P*_*M*+1_ diffuses freely in the cytosolic volume with the diffusion rate *D*_cyt_ and therefore its local concentration is described in units molecules per volume. The dephosphorylation rate $v_{M+1}^{d}$ in the cytosol is given in molecules per volume and time. Note that the inactivation rate and *v*^*i*^ can be invoked by membrane-bound phosphatases or saturation of phosphorylation at the membrane. Both, *v*^*a*^ and *v*^*i*^, comprise the kinetics at the membrane-cytosolic interface. For the flux on all other membrane enclosed organelles we assume a zero-flux condition

$$\begin{array}{cc} -D_{\text{cyt}}\nabla P_{M+1}\cdot \vec{n} & =0. \end{array}$$ (4)

The equations for the components of the downstream cytosolic cascade read

$$\begin{array}{ccc} \frac{\partial P_{n}}{\partial t} & =D_{\text{cyt}}\Delta P_{n}+v_{n}^{a}-v_{n}^{d}, & \;\text{in}\;\text{the}\;\text{cytosol},\;\text{for}\;n=M+2,\ldots ,N. \end{array}$$ (5)

The concentrations of the cytosolic components at position $\vec{x}$ in the cytosolic volume at time *t* are described by functions $P_{n}\left(\vec{x},t\right)$ with *n* = *M* + 1, …, *N*. For the cytosolic components we assume zero-flux conditions:

$$\begin{array}{ccc} -D_{\text{cyt}}\nabla P_{n}\cdot \vec{n} & =0, & \;\text{on}\;\text{the}\;\text{cell}\;\text{membrane,} \end{array}$$ (6)

$$\begin{array}{ccc} -D_{\text{cyt}}\nabla P_{n}\cdot \vec{n} & =0, & \;\text{on}\;\text{the}\;\text{nuclear}\;\text{membrane,}\\ & & \;\text{for}\;\;n=M+2,\ldots ,N. \end{array}$$ (7)

In classical MAPK cascades the last component of the cascade, which is the phosphorylated MAPK, is imported into the nucleus. Examples range from Hog1 nuclear import in yeast [26, 27] to the import of ERK in mammals [28]. In this case, the boundary condition Eq (7) on the nucleus for the last cytosolic component *P*_*N*_ needs to be modified to

$$\begin{array}{cc} -D_{\text{cyt}}\nabla P_{N}\cdot \vec{n} & =-\epsilon P_{N}, \end{array}$$ (8)

where *ϵ* represents a nuclear-import reaction rate on the nuclear membrane. Unless otherwise stated, a zero-flux boundary condition is assumed on the nucleus throughout this paper.

We will test and compare systems with three components *N* = 3 as shown in Fig 1, where the spatial arrangement of the components is varied. Here, *M* = 2 describes the case of two membrane-bound and one cytosolic element (motif Fig 1B) and *M* = 0 the case of only cytosolic components (motif Fig 1C). In the following the case *M* = 2 is referred to as mixed membrane-cytosolic (MMC) and *M* = 0 as pure cytosolic (PC) cascade.

## Results

### The mixed membrane-cytosolic cascade is strongly size dependent

We start this section with an analysis of a spherical cell and then generalize the analysis to arbitrary cell shapes. A spherical cell of radius *R*_cell_ with a spherical nucleus of radius *R*_nuc_ placed in the center of the cell is assumed in the following.

The input signal is denoted by *P*_0_(*t*) and is assumed to be homogeneous on the cell surface. The concentrations of protein kinases are described by functions *P*_*i*_(*r*, *t*) depending on space and time. Note, since the cellular geometry is radially symmetric and the input signal *P*_0_ acts homogeneously on the cell membrane, these functions depend only on the radial distance from the cell center, denoted by *r*, and time *t*. In the following analysis, the kinetic rates are linearized, meaning that we assume $v_{n}^{a}=\alpha_{n}P_{n-1}$ and $v_{n}^{d}=\beta_{n}P_{n}$ for the phosphorylation and dephosphorylation, respectively. The inactivation rate *v*^*i*^ at the membrane-cytosolic interface is as well linearized by *v*^*i*^ = *γP*_*M*+1_. Note that the interface kinetics can be reformulated as $v_{M+1}^{a}-v^{i}=\gamma \left(\frac{\alpha_{M+1}}{\gamma }P_{M}-P_{M+1}\right)$, from which it can easily be seen that the activation at the membrane saturates at $P_{M+1}=\frac{\alpha_{M+1}}{\gamma }P_{M}$. The model equations for the mixed membrane-cytosolic cascade (MMC) with linearized kinetics read

$$\begin{array}{ccc} \frac{\partial P_{1}}{\partial t} & = & D_{\text{mem}}\Delta_{\Gamma }P_{1}+\alpha_{1}P_{0}-\beta_{1}P_{1}\;\;\text{on}\;\text{the}\;\text{membrane}, \end{array}$$ (9)

$$\begin{array}{ccc} \frac{\partial P_{2}}{\partial t} & = & D_{\text{mem}}\Delta_{\Gamma }P_{2}+\alpha_{2}P_{1}-\beta_{2}P_{2}\;\;\text{on}\;\text{the}\;\text{membrane}, \end{array}$$ (10)

$$\begin{array}{ccc} \frac{\partial P_{3}}{\partial t} & = & D_{\text{cyt}}\Delta P_{3}-\beta_{3}P_{3}\;\;\text{in}\;\text{the}\;\text{cytosol}, \end{array}$$ (11)

and boundary conditions for the cytosolic species *P*_3_:

$$\begin{array}{ccc} -D_{\text{cyt}}\nabla P_{3}\cdot \vec{n} & = & \alpha_{3}P_{2}-\gamma P_{3}\;\;\text{on}\;\text{the}\;\text{membrane}, \end{array}$$ (12)

$$\begin{array}{ccc} -D_{\text{cyt}}\nabla P_{3}\cdot \vec{n} & = & -\epsilon P_{3}\;\;\text{at}\;\text{the}\;\text{nucleus}. \end{array}$$ (13)

There are several estimates of phosphatase activity and diffusion coefficients for MAPK signaling components. The diffusion coefficient, *D*_cyt_, of globular cytosolic proteins has been shown to be in the range 1 - 10 *μm*^2^*s*^−1^, while the diffusion coefficient of membrane-bound components, *D*_mem_, is much lower with a value in the range of 10^−3^ - 0.1 *μm*^2^*s*^−1^ [29–31]. The phosphatase rates *β*_*n*_ range over three orders of magnitude 0.1 - 100 *s*^−1^ [32, 33]. In the case of Fus3, which is the MAPK in the mating pathway of the yeast *S. cerevisiae*, the diffusion coefficient and cytosolic dephosphorylation rate were estimated to be 4.2 *μm*^2^*s*^−1^ and 1 *s*^−1^, respectively [14]. See Table 1 for an overview on parameter values and units.

**Table 1:** An overview on values and parameters. For all parameters given in the table, the units apply to the numerical values in figures and text of the paper.

entity	value	unit	reference	description
*D*_mem_	10^−3^ - 0.1	*μm*^2^*s*^−1^	[29–31]	diffusion coefficient for the membrane-bound species
*D*_cyt_	1.0 - 10.0	*μm*^2^*s*^−1^	[14, 34, 35]	diffusion coefficient for the cytosolic species
*α*_*i*_	1.0 - 10.0	*s*^−1^	[9, 36]	phosphorylation rate
*β*_*i*_	0.1 - 100.0	*s*^−1^	[14, 32, 33, 36]	phosphatase activity
*γ*	0.1 - 100.0	*μm* *s*^−1^	[32, 33]	reaction rate at the membrane- cytosolic interface at the cell membrane
*R*_cell_	2.0 - 50.0	*μm*	[8]	radius of the cell

**Fig 2:**
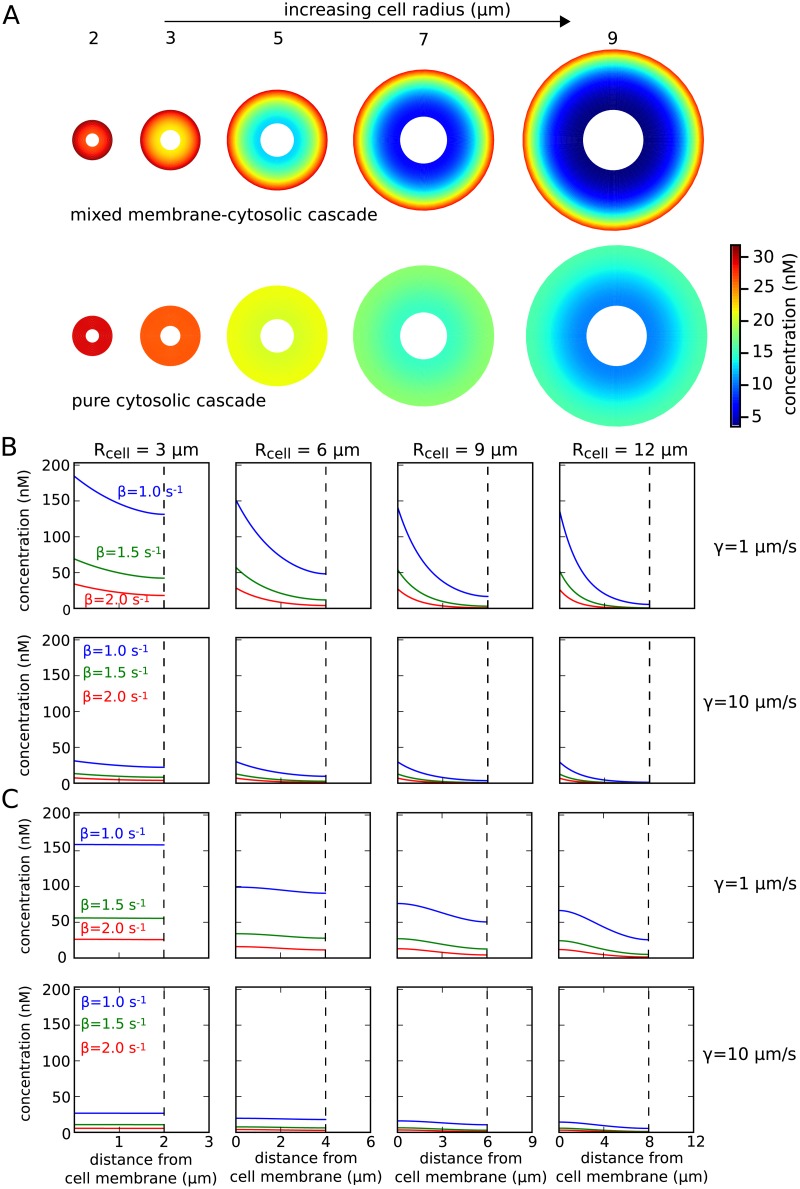
Intracellular concentration profiles for two different signal transduction motifs. (**A**) Concentration of the third cascade element *P*_3_ was plotted along a slice through three-dimensional cells of varying size. Numbers above the cells indicate their radius. Intracellular gradients are steeper for the MMC cascade [upper row] than for the PC cascade [lower row]. The parameters used were *α*_1_ = *α*_2_ = *α*_3_ = 1.5 *s*^−1^, *β*_1_ = *β*_2_ = *β*_3_ = 1.0 *s*^−1^, *D*_mem_ = 0.03 *μm*^2^*s*^−1^, *D*_cyt_ = 3.0 *μm*^2^*s*^−1^, *γ* = 10.0 *μm* *s*^−1^, *P*_0_ ≡ 100 *n*M *μm*. (**B**) Size dependence of the MMC cascade with two membrane-bound and one cytosolic species. (**C**) Size dependence of the PC cascade. The nuclear membrane is indicated by a dashed line.

We begin with a steady state analysis of this system in the parameter regimes of interest and assume that the signal *P*_0_ is constant over time. Here and in the following we indicate the steady distribution with a bar, meaning that ${\bar{P}}_{n}$ denotes the steady state of *P*_*n*_. The steady state of the first two elements is given by ${\bar{P}}_{1}=\frac{\alpha_{1}}{\beta 1}{\bar{P}}_{0}$ and ${\bar{P}}_{2}=\frac{\alpha_{1}\alpha_{2}}{\beta 1\beta_{2}}{\bar{P}}_{0}$. For the steady state of *P*_3_, the solution is given by

$$\begin{array}{c} {\bar{P}}_{3}\left(r\right)=Ai_{0}(r\sqrt{\frac{\beta_{3}}{D_{\text{cyt}}}})+Bk_{0}(r\sqrt{\frac{\beta_{3}}{D_{\text{cyt}}}}), \end{array}$$ (14)

where *i*_0_ and *k*_0_ are modified spherical Bessel functions of the first and second kind, respectively [37]. Note that *i*_0_ is increasing with *r* (distance from the cell center), while *k*_0_ is a decreasing function of *r*. The coefficients *A* and *B* are derived in S1 Appendix. If we neglect the nucleus or there is no nucleus in the cytosol, meaning *R*_nuc_ = 0, the coefficient *B* becomes zero. The steady state solution for different cell sizes is shown in Fig 2.

The concentration is maximal at the cell membrane and decays towards the nucleus. An estimate of the decay length *L*_gradient_ of the intracellular gradient (with highest concentration at the membrane) is given by $L_{\text{gradient}}=\sqrt{D_{\text{cyt}}/\beta_{3}}$ [32]. This decay length can be compared with the actual cell size. Their ratio is called the Thiele modulus, a dimensionless measure defined as $\Phi =R_{\text{cell}}/L_{\text{gradient}}=\sqrt{\beta_{3}R_{\text{cell}}^{2}/D_{\text{cyt}}}$ [33]. For Φ ≫ 1 strong intracellular gradients and concentration heterogeneities of signaling molecules are to be expected, while for Φ ≪ 1 the concentration is almost homogeneous. Since the Thiele modulus relates the diffusion coefficient and degradation rate to cell size, it is an important parameter to investigate gradient formation [33] and signal propagation for several cascade levels [38]. However, in a three-dimensional space *L*_gradient_ can not be interpreted as the actual gradient anymore, since its derivation is based on the assumption of a one-dimensional geometry. In addition, if an excluding volume such as the nucleus is assumed, the one-dimensional *L*_gradient_ overestimates the concentration gradient. For example, if we assume *D*_cyt_ = 4.0 *μm*^2^/*s* and *β* = 1.0 *s*^−1^, we obtain *L*_gradient_ = 2.0 *μm*. In the case of the classical one-dimensional simplification a decay proportional to ∝ exp(−*x*/*L*_gradient_) is assumed, which suggests a concentration decrease in a distance of *x* = 2 *μm* by a factor of exp(−*x*/*L*_gradient_) ≈ 0.37. However, in a spherical cell with radius *R*_cell_ = 3 *μm* with excluding volume *R*_nuc_ = 1 *μm*, the concentration decreases only by a factor of 0.77 in a distance of 2 *μm* from the cell membrane. The effect of cell size on intracellular concentration gradients is shown in Fig 2.

The cell size dependence in cell signaling systems does not only arise by the characteristic length scale for intracellular gradient formation, but by the change of average intracellular concentration levels with cell size. We start with the simplifying assumption that there is no nucleus or excluding volume in the cytosol, meaning *R*_nuc_ = 0. In this case the steady state solution reads

$$\begin{array}{c} {\bar{P}}_{3}\left(r\right)=\frac{\alpha_{3}{\bar{P}}_{2}}{\sqrt{D_{\text{cyt}}\beta_{3}}i_{1}(\Phi )+\gamma i_{0}(\Phi )}i_{0}(r\sqrt{\frac{\beta_{3}}{D_{\text{cyt}}}}). \end{array}$$ (15)

The modified spherical Bessel functions *i*_0_ and *i*_1_ are monotonically increasing functions with $\lim_{\Phi \to 0}i_{0}\left(\Phi \right)=1$ and $\lim_{\Phi \to 0}i_{1}\left(\Phi \right)=0$. We obtain ${\bar{P}}_{3}\left(R_{\text{cell}}\right)\approx \alpha_{3}{\bar{P}}_{3}/\gamma$ for cells with small Φ and, therefore, the phosphorylation reaction at the membrane is at saturation in this case. For large Φ, we obtain from $\lim_{\Phi \to \infty }i_{1}\left(\Phi \right)/i_{0}\left(\Phi \right)=1$ the lower bound ${\bar{P}}_{3}\left(R_{\text{cell}}\right)\approx \alpha_{3}{\bar{P}}_{3}/\left(\sqrt{D_{\text{cyt}}\beta_{3}}+\gamma \right)$. These estimates also hold in the case of an excluding volume which is the nucleus and we obtain the estimate for the concentration ${\bar{P}}_{3}\left(R_{\text{cell}}\right)$ at the cell membrane:

$$\begin{array}{c} \frac{\alpha_{3}{\bar{P}}_{2}}{\sqrt{D_{\text{cyt}}\beta_{3}}+\gamma }\leq {\bar{P}}_{3}\left(R_{\text{cell}}\right)\leq \frac{\alpha_{3}{\bar{P}}_{2}}{\gamma }. \end{array}$$ (16)

**Fig 3:**
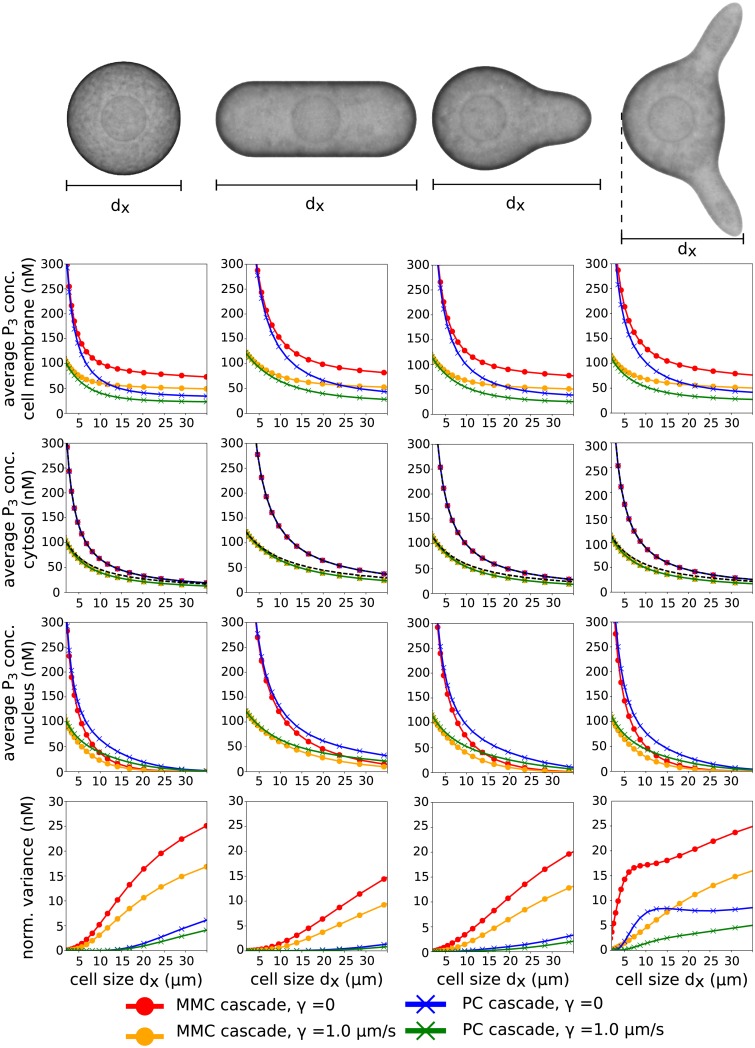
Dependence of concentration levels on cell size and shape. The average concentration of the third cascade element on the cell-membrane [first row], in the cytosol [second row], at the nucleus [third row] as well as the normalized variance [fourth row] was plotted against the cell diameter *d*_*x*_. With variation of of *d*_*x*_, cell shapes were scaled proportionally in y and z direction. The parameters used were *α*_1_ = *α*_2_ = *α*_3_ = 1.5 *s*^−1^ and *β*_1_ = *β*_2_ = *β*_3_ = 1.5 *s*^−1^, *D*_mem_ = 0.03 *μm*^2^*s*^−1^, *D*_cyt_ = 3.0 *μm*^2^*s*^−1^ and *P*_0_ ≡ 100 *n*M *μm*. Simulations of the MMC cascade [dots] and the [crosses] were performed for *γ* = 0 [blue and red] as well as *γ* = 1.0 *μm* *s*^−1^ [orange and green]. The average concentration in the cytosol are exactly the same for both cascades [second column]. For all shapes the average concentration is exactly approximated by Λ_3_ (MMC) or $\frac{\alpha_{2}\alpha_{3}}{\beta_{2}\beta_{3}}\Lambda_{1}$ (PC), while for *γ* = 1.0 *μm* *s*^−1^ the approximation slightly overestimates the average concentration [dashed line].

The dependence of absolute concentration levels on the membrane-cytosolic interface is shown in Figs 2 and 3 for a set of different parameters. For a large inactivation rate at the membrane-cytosolic interface $\gamma >\sqrt{D_{\text{cyt}}\beta_{3}}$, the cell size dependence decreases. Therefore, cell size dependence is mainly determined by *γ* and $\sqrt{D_{\text{cyt}}\beta_{3}}$ but is independent of the phosphorylation rate *α*.

We can further investigate the evolution of the average concentration levels, which depends on the concentration at the cell membrane and the strength of the intracellular gradient. In case of arbitrary cell shapes with cell volume *V*_cell_ and cell membrane area *M*_cell_, the average concentration is obtained from

$$\begin{array}{ccc} P_{m}^{\text{avg}} & = & \frac{1}{|M_{\text{cell}}|}\int_{M_{\text{cell}}}P_{m}dA\;\text{for}\;1\leq m\leq M,\;\text{(membrane-bound}\;\text{components),}\\ P_{n}^{\text{avg}} & = & \frac{1}{|V_{\text{cell}}|}\int_{V_{\text{cell}}}P_{n}dV\;\text{for}\;M+1\leq n\leq N,\;\text{(cytosolic}\;\text{components).} \end{array}$$ (17)

In [33], analytical solutions for the average concentration in a spherical cell and a slab have been derived as functions of the Thiele modulus. However, since the derivations are restricted to cell geometries, where an explicit analytical solution of the reaction diffusion equation is available, we introduce an alternative approach to estimate average concentration levels depending on the cytosolic volume and cell membrane area. Since the signal propagates from the cell membrane to the cytosol, the cell membrane can be regarded as a source, while the cytosolic volume, where phosphorylated signaling molecules are dephosphorylated, can be regarded as a sink. This idea can be derived mathematically by integration of Eq (2) and application of Green’s theorem, which results in

$$\begin{array}{c} \alpha_{M+1}|M_{\text{cell}}|{\bar{P}}_{M}^{\text{avg}}-\gamma \int_{M_{\text{cell}}}{\bar{P}}_{M+1}dA=\beta_{M+1}\left|V_{\text{cell}}\right|{\bar{P}}_{M+1}^{\text{avg}}. \end{array}$$ (18)

Here, the production on the left hand side of the equation depends on the cell membrane area, which is balanced by the degradation in the cytosol on the right hand side of the equation. On the basis of the equation of mass conservation (see S1 Appendix) in reaction diffusion systems, we introduce the following measure:

$$\begin{array}{c} \Lambda_{M+1}=\frac{\alpha_{M+1}\left|M_{\text{cell}}\right|{\bar{P}}_{M}^{\text{avg}}}{\gamma |M_{\text{cell}}|+\beta_{M+1}\left|V_{\text{cell}}\right|}. \end{array}$$ (19)

This measure has the property $\Lambda_{M+1}={\bar{P}}_{M+1}^{\text{avg}}$ for *γ* = 0 or *β* = 0, which holds for arbitrary cell shapes. Furthermore, for a spherical cell the estimate ${\bar{P}}_{M+1}^{\text{avg}}\leq \Lambda_{M+1}\leq {\bar{P}}_{M+1}^{\text{max}}$ holds for *β* > 0 and *γ* > 0 (see S1 Appendix and Fig 3). Therefore, we use Λ_*M*+1_ as a proxy for the average concentration for arbitrary cell shapes, which can be easily calculated.

A comparison of the estimate Λ_3_ to the average concentration is shown in Fig 3 for different cell shapes, which are a spherical cell, a rod shaped cell, a cell with one protrusion and a cell with two protrusions. These cell shapes occur for example in *S. cerevisiae*, *S. pombe* and haploid *S. cerevisiae* stimulated with mating pheromone [39, 40]. The MMC cascade was simulated for these shapes with varying cell size. The measure Λ_3_ is an exact predictor for the average concentration in the case *γ* = 0 for all cell shapes and slightly overestimates the average concentration for *γ* = 1 *μm* *s*^−1^.

Furthermore, we investigated the concentration differences of ${\bar{P}}_{3}$ between cell membrane and nucleus and compared them to the average concentration in the cytosol. For the spherical cell the average concentration levels of ${\bar{P}}_{3}$ in the cytosol as well as on the membrane were the lowest, which is expected since the surface to volume ratio is the lowest among all shapes. The concentration differences between membrane and nucleus were the highest for the spherical cell and the cell with two protrusions and the average concentration at the nucleus decreased almost to zero for large cells. For the rod shape cell the concentration differences were the smallest, since the distance along the short axis is small and the concentration does not drop as sharply as for the other cell shapes.

We furthermore established a correspondence to the evolution of the average concentration levels in time. In the case *γ* = 0 and for arbitrary cell shapes, the average concentration levels follow the system of ordinary differential equations

$$\begin{array}{ccc} \frac{dP_{1}^{\text{avg}}\left(t\right)}{dt} & = & \alpha_{1}P_{0}^{\text{avg}}\left(t\right)-\beta_{1}P_{1}^{\text{avg}}\left(t\right), \end{array}$$ (20)

$$\begin{array}{ccc} \frac{dP_{2}^{\text{avg}}\left(t\right)}{dt} & = & \alpha_{2}P_{1}^{\text{avg}}\left(t\right)-\beta_{2}P_{2}^{\text{avg}}\left(t\right), \end{array}$$ (21)

$$\begin{array}{ccc} \frac{dP_{3}^{\text{avg}}\left(t\right)}{dt} & = & \frac{|M_{\text{cell}}|}{|V_{\text{cell}}|}\alpha_{3}P_{2}^{\text{avg}}\left(t\right)-\beta_{3}P_{3}^{\text{avg}}\left(t\right), \end{array}$$ (22)

where $P_{1}^{\text{avg}}$ and $P_{2}^{\text{avg}}$ are the average concentration levels in molecules per cell membrane area. This system of ordinary equations can be obtained by integrating Eqs (9)–(13) over their respective spatial domains. See S1 Appendix for details of the derivation. The steady state for the average concentration of ${\bar{P}}_{3}$ is given by

$$\begin{array}{c} {\bar{P}}_{3}^{\text{avg}}=\frac{|M_{\text{cell}}|}{|V_{\text{cell}}|}\frac{\alpha_{1}\alpha_{2}\alpha_{3}}{\beta_{1}\beta_{2}\beta_{3}}{\bar{P}}_{0}^{\text{avg}}. \end{array}$$ (23)

Therefore, the average concentration level scales with the ratio of membrane area to cytosolic volume which is given by $\frac{|M_{\text{cell}}|}{|V_{\text{cell}}|}$. The effective global phosphorylation rate for the average concentration of active signaling molecules in the cytosol is therefore determined by ${\tilde{\alpha }}_{3}=\frac{|M_{\text{cell}}|}{|V_{\text{cell}}|}\alpha_{3}$. These relations give us a correspondence between widespread used ordinary differential equations and the bulk-surface partial differential equations employed in this paper. In summary, we have strong cell size dependence, with decreasing concentrations for larger cells.

### Efficient cytosolic transport via cytosolic cascades

In the following we consider a pure cytosolic (PC) cascade with three elements, in which all elements diffuse freely through the cytosol. The reaction-diffusion system is given by

$$\begin{array}{ccc} \frac{\partial P_{1}}{\partial t} & = & D_{\text{cyt}}\Delta P_{1}-\beta_{1}P_{1}\;\;\text{in}\;\text{the}\;\text{cytosol}, \end{array}$$ (24)

$$\begin{array}{ccc} \frac{\partial P_{2}}{\partial t} & = & D_{\text{cyt}}\Delta P_{2}+\alpha_{2}P_{1}-\beta_{2}P_{2}\;\;\text{in}\;\text{the}\;\text{cytosol}, \end{array}$$ (25)

$$\begin{array}{ccc} \frac{\partial P_{2}}{\partial t} & = & D_{\text{cyt}}\Delta P_{3}+\alpha_{3}P_{2}-\beta_{3}P_{3}\;\;\text{in}\;\text{the}\;\text{cytosol} \end{array}$$ (26)

with boundary conditions on the membrane

$$\begin{array}{ccc} -D_{\text{cyt}}\nabla P_{1}\cdot \vec{n} & = & \alpha_{1}P_{0}-\gamma P_{1}, \end{array}$$ (27)

$$\begin{array}{ccc} -D_{\text{cyt}}\nabla P_{2}\cdot \vec{n} & = & -D_{\text{cyt}}\nabla P_{3}\cdot \vec{n}=0, \end{array}$$ (28)

and at the nucleus

$$\begin{array}{ccc} -D_{\text{cyt}}\nabla P_{1}\cdot \vec{n} & = & -D_{\text{cyt}}\nabla P_{2}\cdot \vec{n}=0, \end{array}$$ (29)

$$\begin{array}{ccc} -D_{\text{cyt}}\nabla P_{3}\cdot \vec{n} & = & -\epsilon P_{3}. \end{array}$$ (30)

Note that the membrane-cytosolic interface occurs at the first cascade level, meaning that only *P*_1_ is activated at the membrane with rate *α*_1_ *P*_0_ − *γP*_1_. In the special case of *β*_1_ = *β*_2_ = *β*_3_ = *β* analytical approximations to cytosolic cascades in a one-dimensional system have been derived in [41, 42]. While a one-dimensional cellular geometry can be used to study gradient formation qualitatively, spatial effects such as the cell surface to volume ratio are neglected. Therefore, we derived exact analytical solutions to the linear system in three dimensions. The steady state solutions for ${\bar{P}}_{n}\left(r\right)$ are expanded as follows

$$\begin{array}{ccc} {\bar{P}}_{n}\left(r\right) & = & \sum_{k=1}^{n}A_{n,k}r^{k-2}\exp\left(\sqrt{\frac{\beta }{D_{\text{cyt}}}}r\right)+\sum_{k=1}^{n}B_{n,k}r^{k-2}\exp\left(-\sqrt{\frac{\beta }{D_{\text{cyt}}}}r\right). \end{array}$$ (31)

The algebraic expressions of the coefficients *A*_*n*, *k*_ and *B*_*n*, *k*_ and their derivation are given in the S1 Appendix. In comparison to the MMC cascade, which was discussed in the previous section, the third cascade element *P*_3_ is more evenly distributed in the cell and concentration gradients are much more shallow (see Fig 2).

In order to quantify the concentration differences in a cell of arbitrary shape, we measure the concentration variance in the cell. Therefore, we introduce the variance as

$$\begin{array}{cc} \Sigma_{n}^{2} & =\int_{V_{\text{cell}}}{\left({\bar{P}}_{n}-{\bar{P}}_{n}^{\text{avg}}\right)}^{2}dV\;\;\text{(Variance).} \end{array}$$ (32)

This measure has a close correspondence to the variance in image analysis [43, 44]. In contrast to image analysis, where the square of the deviation of the fluorescence intensity from the average fluorescence intensity of a marker is integrated pixel-wise, the integration here is continuous. As for the analog in image analysis, the normalized variance is calculated as a measure for the deviation from the average [44, 45]. While this measure is frequently used in auto-focus algorithms in image analysis, we suggest the normalized variance as a measure for the degree of localization of signaling molecules within a cell. An estimate the propagation of the normalized variance in the cytosolic cascade is given by

$$\begin{array}{ccc} \frac{\Sigma_{n}^{2}}{{\bar{P}}_{n}^{\text{avg}}\left|V_{\text{cell}}\right|} & \leq & C_{n}\frac{\Sigma_{n-1}^{2}}{{\bar{P}}_{n-1}^{\text{avg}}\left|V_{\text{cell}}\right|},\;\text{with}\;C_{n}=\frac{\alpha_{n}\beta_{n}}{{\left(\frac{D_{\text{cyt}}}{C_{s}^{2}d^{2}}+\beta_{n}\right)}^{2}}. \end{array}$$ (33)

Here, *C*_*s*_ is a constant depending on cell shape and *d* is the cell diameter. Note that *C* = *C*_*s*_*d* is the Poincaré constant from the well known Poincaré inequality [46]. For convex cell shapes the estimate holds for $C_{s}=\frac{1}{\pi }$. In the case of a convex cell shape of a small cell as yeast (without protrusion), we therefore have the estimate *C*_*n*_ ≈ 0.3 ≤ 1 for *D*_cyt_ = 3 *μm*^2^/*s*, *α*_*n*_ = *β*_*n*_ = 1 *s*^−1^ and a cell diameter of *d* = 6 *μm*. For this parameter set, the normalized variance decreases at least by 70% at the second cytosolic cascade level and by 90% at the third cytosolic cascade level (compared to the first cascade element). In general, for *C*_*n*_ < 1, the normalized variance of the intracellular concentrations decreases with increasing cascade level and concentration differences in the cell are balanced out (see Fig 3).

Similar to the previous section, we derived an estimate for the average concentration. In this case, we employed the estimate Λ_1_ to ${\bar{P}}_{1}^{\text{avg}}$, since *P*_1_ is the cascade element that is activated at the membrane-cytosolic interface for the PC cascade. The average concentration ${\bar{P}}_{3}^{\text{avg}}$ is related to ${\bar{P}}_{1}^{\text{avg}}$ by ${\bar{P}}_{3}^{\text{avg}}=\frac{\alpha_{2}\alpha_{3}}{\beta_{2}\beta_{3}}{\bar{P}}_{1}^{\text{avg}}$ and, therefore, we used the approximation $\frac{\alpha_{2}\alpha_{3}}{\beta_{2}\beta_{3}}\Lambda_{1}$ for ${\bar{P}}_{3}^{\text{avg}}$. As in the case of the MMC, this approximation is exact for *γ* = 0 and overestimates the average concentration sligthly for *γ* = 1 *μm* *s*^−1^ (see Fig 3).

Exact expressions for the steady states of the average concentration of signaling components in the case *γ* = 0 and for arbitrary cell shapes are given by

$$\begin{array}{c} {\bar{P}}_{1}^{\text{avg}}=\frac{|M_{\text{cell}}|}{|V_{\text{cell}}|}\frac{\alpha_{1}}{\beta_{1}}{\bar{P}}_{0}^{\text{avg}},\;{\bar{P}}_{2}^{\text{avg}}=\frac{|M_{\text{cell}}|}{|V_{\text{cell}}|}\frac{\alpha_{1}\alpha_{2}}{\beta_{1}\beta_{2}}{\bar{P}}_{0}^{\text{avg}},\;{\bar{P}}_{3}^{\text{avg}}=\frac{|M_{\text{cell}}|}{|V_{\text{cell}}|}\frac{\alpha_{1}\alpha_{2}\alpha_{3}}{\beta_{1}\beta_{2}\beta_{3}}{\bar{P}}_{0}^{\text{avg}}. \end{array}$$ (34)

Therefore, the average concentration of the third cascade element ${\bar{P}}_{3}^{\text{avg}}$ takes the same values in the MMC and PC cascade. The major distinction of both spatial motifs is given by the fact that the concentration differences obtained at the cell membrane and nucleus are larger in the MMC cascade than in the PC cascade. Similarly as in the previous section, we can formulate a system of ordinary differential equations for the time evolution of average concentrations

$$\begin{array}{ccc} \frac{dP_{1}^{\text{avg}}\left(t\right)}{dt} & = & \frac{|M_{\text{cell}}|}{|V_{\text{cell}}|}\alpha_{1}P_{0}^{\text{avg}}\left(t\right)-\beta_{1}P_{1}^{\text{avg}}\left(t\right), \end{array}$$ (35)

$$\begin{array}{ccc} \frac{dP_{2}^{\text{avg}}\left(t\right)}{dt} & = & \alpha_{2}P_{1}^{\text{avg}}\left(t\right)-\beta_{2}P_{2}^{\text{avg}}\left(t\right), \end{array}$$ (36)

$$\begin{array}{ccc} \frac{dP_{3}^{\text{avg}}\left(t\right)}{dt} & = & \alpha_{3}P_{2}^{\text{avg}}\left(t\right)-\beta_{3}P_{3}^{\text{avg}}\left(t\right). \end{array}$$ (37)

The dependence of absolute concentration levels on the membrane-cytosolic interface is shown in Fig 2 and figure in S1 Appendix for a set of different parameters.

### The timing of spatial signaling

Time-resolved image-based analysis has shown that MAPK signaling pathways respond with a measurable signal in the nucleus in time scales of seconds to a few minutes. The Hog1 pathway response (phosphorylated MAPK) in budding yeast is at about 80% of its maximal activity within a minute [26]. Another example is the Src activation/deactivation cycle, where oscillations and pulses take place in the regime of seconds [47]. The timing of signal transduction in linear signaling cascades for well-stirred homogeneous systems has been analyzed in [9]. They concluded for weakly activated signaling cascades that phosphatases have a more pronounced effect than kinases on the rate and duration of signaling, whereas signal amplitude is controlled primarily by kinases. A thorough analysis of linear models assuming a homogeneous distribution of signaling molecules for different kinds of external stimuli has been recently worked out in [12]. We extended and compared these findings to spatial signal transduction omitting the simplification of homogeneous concentrations. How long does it take to establish an intracellular concentration gradient? How does diffusion change the timing of signal transmission from the cell membrane to nucleus? Which concentration differences are expected until a steady state is established? The time-scale analysis for spatial models is more difficult than for models based on a will-mixed assumptions due to high computational costs. Therefore, we used the recently introduced measure of accumulation times [25, 48]. The approach of *P*_*n*_(*r*, *t*) to its steady state ${\bar{P}}_{n}\left(r\right)$ at radial distance *r* from the cell center and time *t* can be characterized using the local relaxation function

$$\begin{array}{ccc} \rho_{n}\left(r,t\right) & = & \left({\bar{P}}_{n}\left(r\right)-P_{n}\left(r,t\right)\right)/{\bar{P}}_{n}\left(r\right). \end{array}$$ (38)

The difference *ρ*_*n*_(*r*, *t*_1_) − *ρ*_*n*_(*r*, *t*_2_) can be interpreted as the fraction of the steady state level ${\bar{P}}_{n}\left(r\right)$ that accumulated in the time interval [*t*_1_, *t*_2_]. In an infinitesimal time interval [*t*, *t* + *dt*] the fraction of accumulated activated signaling molecules at steady state is given by $-\frac{\partial \rho_{n}\left(r,t\right)}{\partial t}dt$. The local accumulation time is defined as [25]

$$\begin{array}{cc} \tau_{n}\left(r\right) & =-\int_{0}^{\infty }t\frac{\partial \rho_{n}\left(r,t\right)}{\partial t}dt. \end{array}$$

The accumulation time can be derived from the steady state solution even if no closed form of the time-dependent solution is known [25].

The timing of the average concentrations given in the system of ordinary differential equations for the MMC cascade (20)–(22) and the PC cascade (35)–(37) are the same and can be analytically expressed as

$$\begin{array}{c} \tau_{3}=\frac{1}{\beta_{1}}+\frac{1}{\beta_{2}}+\frac{1}{\beta_{3}}. \end{array}$$ (39)

**Fig 4:**
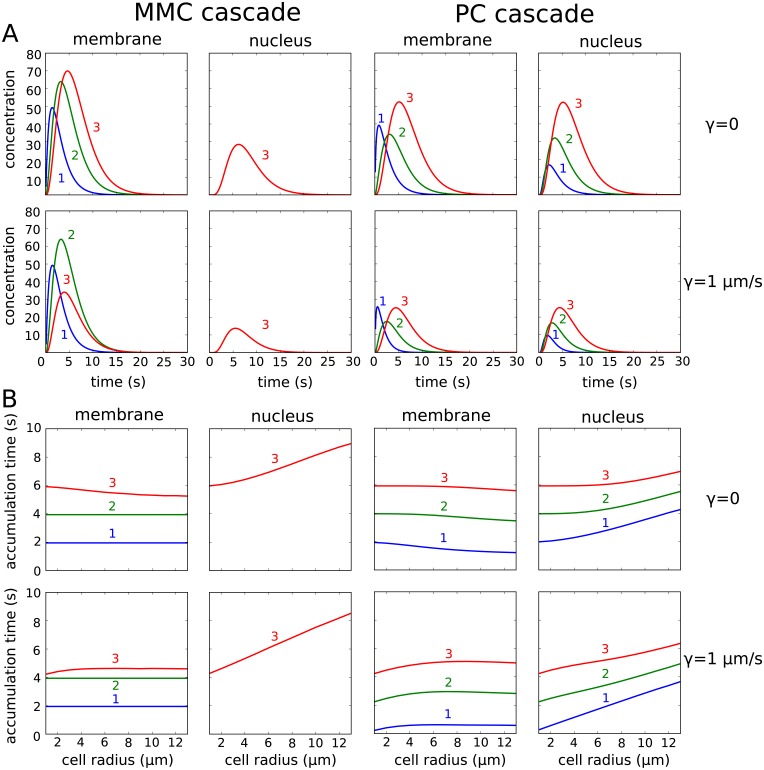
Timing of spatial signaling. The signaling time for the mixed membrane-cytosolic (MMC) cascade [left] and pure cytosolic (PC) cascade [right] at the membrane and at the nucleus was simulated. (**A**) Time course for the concentrations of *P*_1_, *P*_2_ and *P*_3_ after stimulation with a time-dependent signal $P_{0}\left(\vec{x},t\right)=P_{0}^{\text{max}}\exp\left(-\lambda t\right)$ and $P_{0}^{\text{max}}=100\;nM\mu m$ was plotted. The cascade levels are indicated by the numbers. Note that in case of the MMC, the concentrations for *P*_1_ and *P*_2_ are given in *n*M*μm*, while the concentration of *P*_3_ is given in *n*M. For the PC cascade all concentrations *P*_1_, *P*_2_ and *P*_3_ are given in *n*M. The parameters used were *R*_cell_ = 6 *μm*, *R*_nuc_ = 2 *μm*, λ = 1 *s*^−1^, *α*_1_, *α*_2_, *α*_3_ = 1.0 *s*^−1^, *β*_1_, *β*_2_, *β*_3_ = 0.5 *s*^−1^, *D*_mem_ = 0.03 *μm*^2^*s*^−1^ and *D*_cyt_ = 3.0 *μm*^2^*s*^−1^. This setup was simulated for *γ* = 0 and *γ* = 1 *μm*/*s*. (**B**) Accumulation times for the mixed membrane-cytosolic cascade. In this scenario, a constant signal $P_{0}\left(\vec{x},t\right)=100\;nM\mu m$ was applied and the cell size was varied. The ratio of cellular to nuclear radius was kept at *R*_cell_/*R*_nuc_ = 3. Otherwise the same parameters as in (**A**) were used.

This expression also coincides with signaling times calculated by Heinrich et al. [9]. However, for the spatial model the local accumulation times at the membrane and nucleus differ. The accumulation is generally faster at the membrane and slower at the nucleus, where the degree of the difference increases with cell size (see Fig 4). Furthermore, the two spatial motifs show significant differences. For the MMC cascade the accumulation time for the second element *P*_2_ is exactly $\frac{1}{\beta_{1}}+\frac{1}{\beta_{2}}$ on the membrane, while it is shorter for the cytosolic species (compare Fig 4).

The accumulation time of *P*_3_ at the nucleus is, as expected, much longer. For small cells the intracellular concentration is spatially homogeneous and the approximation $\frac{1}{\beta_{1}}+\frac{1}{\beta_{2}}+\frac{1}{\beta_{3}}$ holds, while the time for signal propagation to the nucleus increases with cell size. An analytical solution of the accumulation times for *P*_3_ for the MMC cascade and the special case of *R*_nuc_ = 0 can be derived [49], which is given in the S1 Appendix. However, for larger cells, the time for signal propagation to the nucleus increases with cell size. For the PC cascade, the increase in accumulation time at the nucleus with cell size is less pronounced than for the mixed-membrane cytosolic cascade.

While a constant stimulus was applied to calculate the accumulation times, we also tested a decaying signal $P_{0}\left(t\right)=P_{0}^{\text{max}}\exp\left(-\lambda t\right)$, with $P_{0}^{\text{max}}=100\;nM\mu m$ and solved the time-dependent system numerically. A comparison of the MMC and PC is shown in Fig 4. Interestingly, the concentration level at the membrane for the PC cascade decreases from the first cascade species *P*_1_ to the second cascade species *P*_2_ and than increases again from the second cascade species *P*_2_ to the third cascade species *P*_3_, while there is an increase from the preceding cascade species to the next cascade species at the nucleus. This phenomenon is caused by the concentration differences from cell membrane to nucleus, which is larger for *P*_1_ than for *P*_2_ in the PC cascade. Note that the parameters were chosen to be $\frac{\alpha_{n}}{\beta_{n}}=2$, which means a twofold increase for the average concentration levels from one signaling cascade element to the next. Therefore, the spatial system can behave entirely different than the homogeneous system. The accumulation time at the membrane was much faster for *γ* = 1 *μm*/*s* than for *γ* = 0 and changed only slightly with cell size. However, for larger cells the accumulation time of the signal at the nucleus was almost independent of *γ*. Therefore, the difference of accumulation times at the membrane and nucleus increased with *γ* (also compare figure in S1 Appendix). In case of the MMC cascade the accumulation time at the nucleus for a cell with *R*_cell_ = 12 *μm* almost doubled compared to a small cell with *R*_cell_ = 2 *μm*, while for the PC cascade the increase of accumulation time with cell size was less pronounced.

For calculation of higher moments of the time scaling and the special case of a cell without nucleus we refer to [49]. An analysis for time scaling of a linear cascade in one spatial dimension with four elements including higher moments has been carried out in [50].

### Quantifying the pathway sensitivity with respect to spatially heterogeneous signals

In the following we analyze signal transduction of heterogeneous external signals. For example, in cultures of mixed haploid yeast cell populations [40] as well as in microfluidic devices [51], the external pheromone signal, which triggers a MAPK cascade, is not homogeneously distributed but forms gradients in the extracellular medium. The activated signaling cascade is spatially localized and triggers subsequent directed growth in *S. cerevisiae* [14] as well as *S. pombe* [15]. Furthermore, properties of protein-protein interactions and morphological changes can be tightly connected [52].

Therefore, we investigate the signal transduction in response to an external heterogeneous signal for same cell shapes as in Fig 3, which were a spherical cell, a rod shaped cell, a cell with one protrusion and a cell with two protrusions. These cell shapes occur for example in *S. cerevisiae*, *S. pombe* and during their response to stimulation with mating pheromone [53].

We tested the linear signaling cascade with a graded stimulus of the form

$$\begin{array}{c} P_{0}\left(\vec{x}\right)=P_{0}^{\text{sig}}[1+P_{0}^{\text{slope}}\left(x_{1}-x_{1}^{\text{mid}}\right)],\;\vec{x}=\left(x_{1},x_{2},x_{3}\right), \end{array}$$ (40)

where $P_{0}^{\text{sig}}$ and $P_{0}^{\text{slope}}$ are constants describing the basal signal strength and the slope of the signal, respectively. Here, we chose the origin of coordinates to be in the center of the cell and, therefore, ${\vec{x}}^{\text{mid}}=\left(0,0,0\right)$. In this way, we obtain an input signal gradient which increases linearly in *x*_1_-direction for $P_{0}^{\text{slope}}>0$ and decreases linearly for $P_{0}^{\text{slope}}<0$. The concentration at $x_{1}^{\text{mid}}$ is given by $P_{0}^{\text{sig}}$.

**Fig 5:**
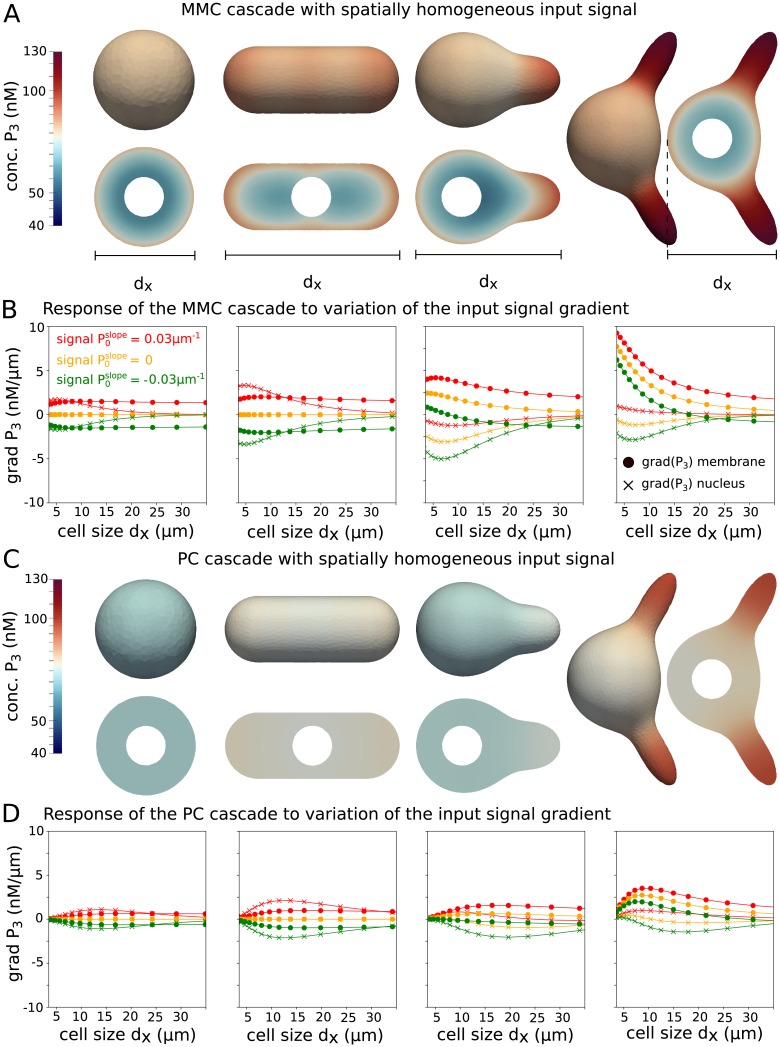
Response of the two cascade systems, MMC and PC, for different cell shapes to a variation of the signal gradient. (**A**) Spatial concentration profile of ${\bar{P}}_{3}$ on the cell membrane as well as along a slice through the cell in response to a homogeneous input signal *P*_0_ ≡ 100 *n*M*μm*. The spherical cell has a size of *R*_cell_ = 2.5 *μm* with a nucleus of radius *R*_nuc_ = 1 *μm*. All cell shapes have the same cell volume and contain a spherical nucleus of the same size. (**B**) Simulations for varying cell size measured as diameter *d*_*x*_ in x-direction and three different signal slopes $P_{0}^{\text{slope}}=-0.03\;\mu m^{-1}$ (green), 0 (orange), 0.03 *μm*^−1^ (red) were performed. The gradient grad ${\bar{P}}_{3}≔\left({\bar{P}}_{3}\left({\vec{x}}_{\text{front}}\right)-{\bar{P}}_{3}\left({\vec{x}}_{\text{back}}\right)\right)/\left|{\vec{x}}_{\text{front}}-{\vec{x}}_{\text{back}}\right|$ was plotted. Here, ${\vec{x}}_{\text{front}}$ and ${\vec{x}}_{\text{back}}$ are the extreme points *x*-direction on the cell membrane or nucleus. In (**C**) and (**D**), the PC cascades was simulated for the same setup as in (**A**),(**B**). The parameters used were *α*_1_ = *α*_2_ = *α*_3_ = 1 *s*^−1^, *β*_1_ = *β*_2_ = *β*_3_ = 1 *s*^−1^, *γ* = 0.5 *μm* *s*^−1^, *D*_mem_ = 0.03 *μm*^2^*s*^−1^ and *D*_cyt_ = 3.0 *μm*^2^*s*^−1^.

We tested the influence of asymmetries in cell shape in response to the graded stimulus Eq (40) and investigated the spatial distribution of the last signaling component of the MMC and PC cascade, which is *P*_3_. In Fig 5(A) and 5(C), the concentration profile of ${\bar{P}}_{3}$ on the cell membrane as well along a slice through the cell in response to a homogeneous signal is shown as control. Since the spherical cell is radially symmetric, no gradient was induced on the membrane. For the rod shaped cell, we observed a shallow gradient on the cell surface with higher concentration at the poles, the intracellular concentration profile exhibited two areas of low concentration, which were separated by the nucleus. This effect was more pronounced for the MMC cascade. For the PC cascade, the concentration was almost homogeneously distributed. For the asymmetric cell shapes with one and two protrusions, a gradient from the distal end (front) to the spherical part (back) was established in response to a homogeneous input signal. The protrusion, therefore, can be compared to a pocket in which higher concentrations of cytosolic signaling molecules are established. Mathematically this effect can be explained by the geometry dependence of the eigenfunctions of the Laplace operator [54], which can be employed to characterize the solution of the reaction-diffusion equations for a certain cell geometry. Therefore, these asymmetric cell shapes can already induce a gradient of signaling molecules from front to back.

In Fig 5(B) and 5(D), the responses to a signal with $P_{0}^{\text{slope}}=0.03\;\mu m^{-1}$, which is increasing in *x*_1_-direction, and a signal with $P_{0}^{\text{slope}}=-0.03\;\mu m^{-1}$, which is decreasing in *x*_1_-direction, were simulated and opposed to the response to a spatially homogeneous signal with $P_{0}^{\text{slope}}=0$. To measure the response, we define the gradient of the *n*-th cascade element naturally as the difference of concentrations at two points over the euclidean distance of these two points. In the case of the kinase concentrations, the gradient was computed from $\left(P_{n}\left({\vec{x}}_{\text{front}}\right)-P_{n}\left({\vec{x}}_{\text{back}}\right)\right)/\left|{\vec{x}}_{\text{front}}-{\vec{x}}_{\text{back}}\right|$. Here, ${\vec{x}}_{\text{front}}$ and ${\vec{x}}_{\text{back}}$ are the extreme points in *x*_1_-direction on the cell membrane or nucleus. Both motifs, the MMC and PC cascade, behave differently in the transduction of signal gradients. The gradient of the third cascade level *P*_3_ along the cell membrane and the nucleus decreased for the MMC cascade with cell size for all shapes. For the PC, the gradient increased with cell size to a maximum value and then decreased for larger cell sizes, which suggests an optimal cell size for gradient detection and transmission. This effect was expected, since for small cells the concentration was almost homogeneous in the cytosol and concentration differences were balanced by diffusion. However, with increasing cell size the average concentration level decreased in the cell and at the nucleus. As a consequence, also the absolute gradient decreased.

The rod shaped cell showed a stronger response than the spherical cell shape, since concentrations were higher at the poles and the cell was aligned along the gradient. Furthermore, the compartmentalization induced by the nucleus in the thin rod shaped cell had a pronounced effect on the *P*_3_ gradient, since diffusion in the cytosol from front to back was hindered. For the cells with one and two protrusions the gradient of *P*_3_ was strongly biased with an increase in the direction of the protrusions. Note that both motifs behave differently for the transmission of the gradient to the nucleus. While for the MMC cascade, the shape dependence was more pronounced and the gradient in the cell interior was almost decoupled from the gradient on the membrane for the asymmetric cell shapes, the PC cascade transmitted the gradient more reliably into the cell interior and the nucleus.

In summary, we observed a strong influence of cell size on localization and establishment of gradients by signaling cascade elements. For the cell with a protrusion the concentration of *P*_3_ was higher in the protrusion than in the opposite distal end, which is the spherical part of the cell. This effect emerged due to a higher local surface to volume ratio in the protrusion region. Therefore, a larger portion of cytosolic signaling molecules, which diffuse freely in the cytosol, is phosphorylated in the protrusion part leading to a gradient from the protrusion tip to the opposite distal end of the cell. The influence of cellular asymmetries has also been investigated in [23] for gradients of the small Rho-GTPase Cdc42 during cell polarization. However, this system reacts in the opposite way, since the flux of molecules during the establishment of a polarity site is directed from the cytosol onto the membrane and, therefore, a gradient from the distal end to the protrusion is established.

These effects occur due to the different architectures of both systems. In the PC and MMC signaling cascades, we have signal transduction from the membrane to the nucleus and, therefore, a diffusive flux of activated signaling molecules from the membrane into the cytosol, while in the polarization system the flux of signaling molecules during the establishment of a polarity site is directed from the cytosol onto the membrane, which is the opposite direction. Therefore, both system respond differently to cellular asymmetries with respect to gradient formation. This interplay of both systems is especially interesting, since in many organisms a polarization system is interacting with a MAPK cascade [55, 56] and might, therefore, precisely control cell shape and size.

For spherical cell shapes we furthermore investigated more complex external signal gradients, meaning heterogeneities with multiple maxima and minima (see S1 Appendix). As in [18, 57], a heterogeneous signal on a sphere can be decomposed using spherical harmonics

$$\begin{array}{ccc} P_{0}\left(\theta ,\phi ,t\right) & = & \sum_{l=0}^{\infty }\sum_{m=-l}^{l}A_{0,l}^{m}\left(t\right)Y_{l}^{m}\left(\theta ,\phi \right), \end{array}$$ (41)

$$\begin{array}{ccc} A_{0,l}^{m}\left(t\right) & = & \int_{0}^{2\pi }\int_{0}^{\pi }P_{0}\left(\theta ,\phi ,t\right)Y_{l}^{m*}\left(\theta ,\phi \right)\sin\left(\theta \right)\;d\theta d\phi . \end{array}$$ (42)

In this decomposition the amplitudes of higher order, where the order is denoted by *l*, are generally more strongly damped than gradients or spatial heterogeneities of lower order. In this manner, the results shown here can be extended to complex spatial signals on the cell surface. We provide full analytical solutions for the MMC and PC for a sphere with excluding nucleus (see S1 Appendix).

### Systems with feedback

In this section, we analyze the influence of cell size on signal transduction for an oscillating cascade consisting of two membrane-bound and one cytosolic member (MMC) and a cascade of three cytosolic elements (PC), meaning for *M* = 2 and *M* = 0, respectively. The case of a negative feedback and a constant homogeneous signal is investigated in the following.

Negative feedbacks are a frequent regulation element in signaling cascades and can be induced by the dephosphorylation of upstream components by the MAPK or phosphatases [39, 58–61]. Examples are given by Tyr phosphatases, which can induce a negative feedback [47] and dual specificity phosphatases (DUSPs) [59]. Some negative feedbacks, as for instance induced in the Src-Tyr cycle, lead to oscillations on the time scale of seconds [47], while others act on much longer time scales. For instance, during the yeast pheromone response the MAPK Fus3 undergoes sustained oscillations in the range of 2-3 hours, which control the periodic formation of mating projections. In this process Sst2 acts as a negative regulator of the G-Protein signaling at the membrane, while deactivation in the cytosol is mediated by the MAPK phosphatase Msg5 [39]. A classical and most simple example of an oscillator with negative feedback and non-linear reaction terms is the Goodwin oscillatory system [62, 63].

We adapt the mentioned, modified system and formulate the problem using partial differential equations by adding a diffusion term and formulating the boundary conditions accordingly to the models mentioned before. The phosphorylation and dephosphorylation rates for both models read as

$$\begin{array}{ccc} v_{1}^{a}= & \frac{P_{0}}{1+{\left(P_{3}/K_{m}\right)}^{p}},\;\; & v_{1}^{d}=\beta_{1}P_{1}, \end{array}$$ (43)

$$\begin{array}{ccc} v_{2}^{a}= & \beta_{2}P_{1},\;\;\; & v_{2}^{d}=\beta_{2}P_{2}, \end{array}$$ (44)

$$\begin{array}{ccc} v_{3}^{a}= & \beta_{3}P_{2},\;\;\; & v_{3}^{d}=\beta_{3}P_{3}, \end{array}$$ (45)

according to Eqs (1)–(5), respectively. The activation rate $v_{1}^{a}$ contains the negative feedback, since a high concentration of *P*_3_ leads to a lower activation of *P*_1_. We assume a constant external signal $P_{0}\left(\vec{x},t\right)=100\;nM\mu m$.

For the first model (MMC), the deactivation with rate $v_{3}^{d}$ takes place in the cytosol, whereas the activation occurs on the membrane and is therefore modeled as a boundary condition with *v*^*i*^ = 0 according to Eqs (2) and (3), as *P*_3_ is a solely cytosolic species. We assume zero-flux conditions for *P*_3_ on the nucleus, meaning that we set the nuclear-import reaction rate *ϵ* = 0 (compare Eq (8)).

For the second model (PC), all species are solely cytosolic, hence the activation rate $v_{1}^{a}$ for *P*_1_ is a boundary condition describing the activation of *P*_1_ on the membrane. We assume a zero-flux condition for *P*_3_ on the nucleus (*ϵ* = 0) and for *P*_1_ and *P*_2_ on both nucleus and membrane, meaning the whole boundary.

Both models contain non-linear kinetics as well as negative feedbacks, resulting in oscillations. Furthermore, in both models the activation rate for *P*_1_ depends on a parameter *p* > 0. It is shown, e.g. in [64], that for *p* > 8 the ODE system consisting of three species destabilizes, and that for longer cascades, i.e. for larger *N*, the system becomes instable for even lower values of *p* > 1.

Since an analytical solution is unknown for both models, numerical methods have to be employed to solve the systems. For simplicity reasons and due to the high computational overhead we solved the systems in two dimensions, using a disc to model the cell. We used a fixed-point scheme to solve the non-linear equations.

**Fig 6:**
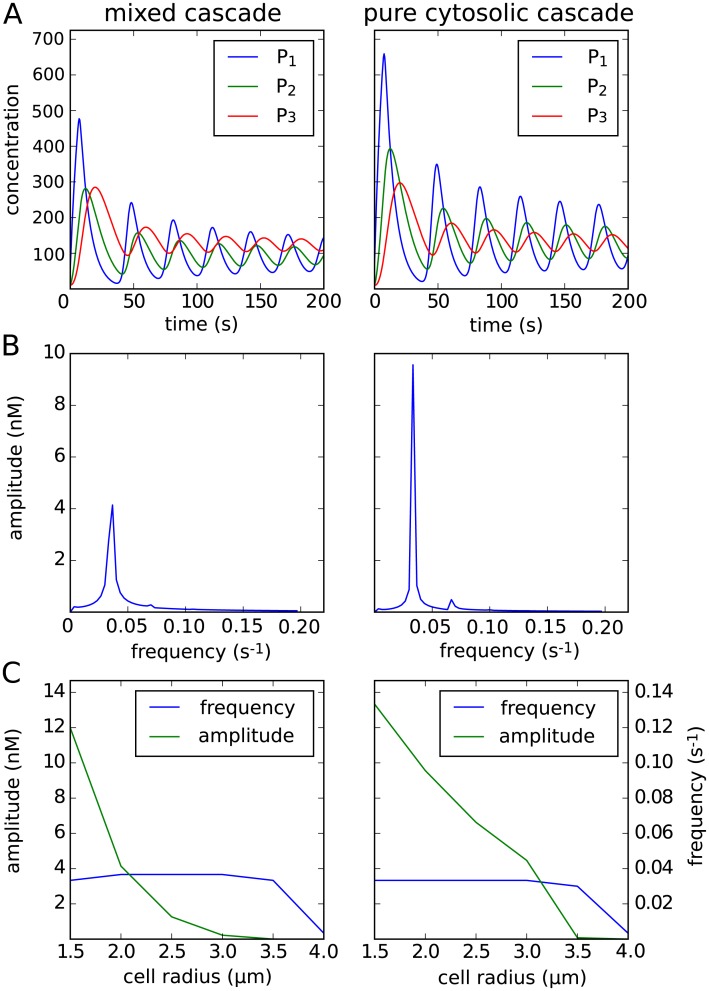
Size-dependent oscillations in a system with negative feedback. (**A**) Mean concentration of the *P*_1_, *P*_2_ and respectively *P*_3_ over time. After an initial peak both systems oscillate. Note that in case of the MMC, *P*_1_, *P*_2_ concentration levels are given in *n*M*μm*, while *P*_3_ concentration is given in *n*M. For the PC cascade all concentrations *P*_1_, *P*_2_ and *P*_3_ are given in *n*M. (**B**) Frequency analysis of the mean concentration of both models for the last species, *P*_3_. (**C**) Frequency and amplitude of the mean concentration of *P*_3_ for the two models in dependence of the cell size.

We chose the parameters *β*_1_ = *β*_2_ = *β*_3_ = 0.125 *s*^−1^, *D*_cyt_ = 1 *μm*^2^*s*^−1^, *D*_mem_ = 0.01 *μm*^2^*s*^−1^, *R*_nuc_ = 1 *μm* and *R*_cell_ = 2 *μm*. The initial conditions were *P*_1_ = *P*_2_ = 10 *n*M*μm* and *P*_3_ = 10 *n*M for the MMC cascade and *P*_1_ = *P*_2_ = *P*_3_ = 10 *n*M for the PC cascade. For *K*_*m*_ = 100 *n*M and the feedback strength *p* = 10, both models oscillated as expected, Fig 6A.

Therefore, in the case of a relatively small cell size of *R*_cell_ = 2 *μm*, both spatial models behave similarly to the original model, which was formulated as a system of ordinary differential equations. An analysis of the oscillation frequencies and the mean concentration can be seen in Fig 6B. Based on previous experiments and plots, the frequency analysis was conducted after *t* = 200 *s*, when the frequency and corresponding amplitudes of the mean concentrations for both models can be assumed to be constant. Both models show a very similar behavior, whereas the frequency and mean concentration are higher for the pure cytosolic model, as can be explained by the fact that the reactions do not only occur at the membrane, but everywhere in the cytosol.

In a next step we varied the cell size 1.5 *μm* ≤ *R*_cell_ ≤ 4.0 *μm* and again conducted an analysis of the frequencies and corresponding amplitudes for both models. The results of the analysis for the third component of both models are plotted in Fig 6C. As pointed out before, the frequency for both models is very similar, but the amplitude of the signal is higher for the cytosolic model.

Oscillations in the first, mixed cascade model only occur for a cell size up to *R*_cell_ ≤ 2.5 *μm*, and in the second, pure cytosolic model for a cell size up to *R*_cell_ ≤ 3.0 *μm*. The inital oscillations die down fast and both models converge against a steady state if the cell size is chosen bigger.

## Discussion

Stimulated by the progress in cell imaging and the increasing need to understand intracellular dynamics, we investigated and discussed a general approach of modeling cellular signal transduction in time and space. Signaling cascades of covalent protein modifications, such as mitogen-activated protein-kinase (MAPK) cascades and small GTPase cascades, occur in a plethora of variations [1, 13, 65]. The first signal component can be activated at the cell membrane by a membrane-bound enzyme such as a kinase or a guanine nucleotide exchange factor in GTPase signaling, while deactivation can occur at the membrane or in the cytosol, for instance, mediated by a phosphatase or GTPase activating protein [66]. Therefore, activation and deactivation can be spatially separated, which creates a number of different spatial arrangements and combinations in signal transduction.

We investigated signaling cascades with different spatial arrangements of signaling components. We showed that modeling of the membrane-cytosolic interface is crucial as well as the ratio of membrane area and cytosolic volume, which are both spatial properties. The results imply strong cell size and shape dependence of signal transduction within cells, which are likely to contribute to single cell variation in response to extracellular stimuli. We suggest that cells measure the cell membrane to cell volume ratio to coordinate growth and differentiation. For asymmetric cell shapes also local changes in cell volume to cell membrane ratio becomes important for intracellular signaling. Widely used time-dependent models of ordinary differential equations can naturally be extended into space by using bulk-surface differential equations. Applying this extension to a class of linear signal transduction models, we compared the assumption of a well mixed cell with two different spatial signal transduction motifs. We derived and discussed criteria that can be used to test the well-mixed assumption and showed that kinetics that connect membrane-bound species with cytosolic species naturally cause size dependence. The results are, therefore, of general importance for kinetic models of signal transduction.

Our findings have relevant biological implications. Since the signals transduced by linear signaling cascades from the cell membrane to the nucleus decrease exponentially on a length scale of a few microns, our theoretical findings suggest a strong cell size dependence in response to extracellular stimuli. Furthermore, the global cell volume to cell membrane area is important for average concentration levels. Mating projections as they occur in yeast act as pockets for signaling molecules, which can support biochemical feedbacks. Adaptations as lamellipodia in keratocytes or invaginations such as T-tubuli in myocytes can locally increase the accumulation of signaling molecules. These cellular structures are able to directly provide a feedback on signaling.

We suggest the normalized variance as a measure to quantify concentration differences and localization of signaling molecules, which can be obtained from spatially resolved microscopy data additionally to mean intensity levels of a fluorescence marker. For example, it would be enlightening to measure average concentration and normalized variance together with cell size and morphology. Interesting studies of the response in cell populations often lack the response behavior attributed to cell size and morphology. Examples range from the switch-like behavior in populations of oocytes [67] to the pheromone response in yeast cells [68, 69]. Therefore, single cell data where the cell size is assigned to these measurements is needed for a faithful quantitative investigation of the pathway, to disentangle biochemical properties of protein-protein interactions and morphological properties such as size and shape of whole cells. Targeting signaling proteins by lipidation modifications such as palmitoylation, prenylation or myristoylation [2, 3, 5] could change the sequestration of a signaling cascade from a pure cytosolic (PC) cascade to a mixed-membrane bound (MMC) cascade. In the case of the mixed-membrane MMC the geometry and size dependence is more pronounced, since the first signaling elements are tethered to the membrane. In contrast, for the investigated PC cascade, localization and strong intracellular gradients are reduced, but depending on the kinetic parameters, the geometric information can also be better transmitted through the whole cell.

In non-linear signaling systems, the differences that we observed in the linear signaling cascade models are likely to be amplified. Non-linear kinetics can amplify gradient formation, which leads to even stronger intracellular concentration differences [70]. This also holds for absolute concentration levels that can behave in a switch-like manner depending on the kinetics [67, 71]. Furthermore, higher order kinetics can amplify the accumulation time differences in different cellular locations [72], which can lead to spatial oscillations and phosphoprotein waves.

The analysis of the signaling cascade model can be extended to more complex spatial heterogeneities for example by using the Laplace series as suggested in [18, 57]. With this approach localized signals arising from membrane structures like lipid rafts, septins or co-localization due to protein-protein interactions can be represented. Since these are often precursors for cell shape and organelle structures, the interplay with cell shape and morphology needs to be addressed by future research. The intrinsic geometry dependence of signaling systems has recently been shown for ellipsoidal cell shapes in the MinE-MinD system [24, 73, 74], but also in the yeast system [23, 75–77]. Recent developments of mathematical methods such as the finite element method for bulk-surface equations [19, 20] as well as stability analysis techniques of these systems [23, 78–82] are expected to provide further insight in the behavior of cellular signal transduction.

## Methods

We used the finite-element software FEniCS [83, 84] to solve the arising partial differential equations in the Python programming language. The meshes were generated using the computational geometry algorithms library (CGAL) [85]. The non-linear equations were solved using a fixed-point scheme.

## Supporting information

S1 Appendix The appendix contains all derivations of the analytical solutions for the steady state of the MMC and PC cascades as well as a general cascade with an arbitrary number of elements. The correspondence of the homogeneous ordinary differential equation system to the spatial MMC and PC system is established. Furthermore, an analytical expression for accumulation time of the MMC cascade is derived.
(PDF)

## References

[1] Bhattacharyya RP, Reményi A, Yeh BJ, Lim WA. Domains, motifs, and scaffolds: the role of modular interactions in the evolution and wiring of cell signaling circuits. Annu Rev Biochem. 2006;75:655–680. doi: 10.1146/annurev.biochem.75.103004.142710 16756506 10.1146/annurev.biochem.75.103004.142710

[2] McLaughlin S, Aderem A. The myristoyl-electrostatic switch: a modulator of reversible protein-membrane interactions. Trends in biochemical sciences. 1995;20(7):272–276. doi: 10.1016/S0968-0004(00)89042-8 7667880 10.1016/s0968-0004(00)89042-8

[3] Rocks O, Peyker A, Kahms M, Verveer PJ, Koerner C, Lumbierres M, et al An acylation cycle regulates localization and activity of palmitoylated Ras isoforms. Science. 2005;307(5716):1746–1752. doi: 10.1126/science.1105654 15705808 10.1126/science.1105654

[4] Gelb MH, Brunsveld L, Hrycyna CA, Michaelis S, Tamanoi F, Van Voorhis WC, et al Therapeutic intervention based on protein prenylation and associated modifications. Nature chemical biology. 2006;2(10):518–528. doi: 10.1038/nchembio818 16983387 10.1038/nchembio818 PMC2892741

[5] Hayashi N, Titani K. N-myristoylated proteins, key components in intracellular signal transduction systems enabling rapid and flexible cell responses. Proceedings of the Japan Academy, Series B. 2010;86(5):494–508. doi: 10.2183/pjab.86.494 10.2183/pjab.86.494 PMC3108300 20467215

[6] Wang M, Casey PJ. Protein prenylation: unique fats make their mark on biology. Nature Reviews Molecular Cell Biology. 2016;17(2):110–122. doi: 10.1038/nrm.2015.11 26790532 10.1038/nrm.2015.11

[7] Gordley RM, Bugaj LJ, Lim WA. Modular engineering of cellular signaling proteins and networks. Current opinion in structural biology. 2016;39:106–114. doi: 10.1016/j.sbi.2016.06.012 27423114 10.1016/j.sbi.2016.06.012 PMC5127285

[8] Alberts B, Johnson A, Lewis J, Raff M, Roberts K, Walter P. Molecular Biology of the Cell, 5th edn, Garland Science, New York. ISBN. 2007;1174808063:1392.

[9] Heinrich R, Neel BG, Rapoport TA. Mathematical models of protein kinase signal transduction. Molecular cell. 2002;9(5):957–970. doi: 10.1016/S1097-2765(02)00528-2 12049733 10.1016/s1097-2765(02)00528-2

[10] Kofahl B, Klipp E. Modelling the dynamics of the yeast pheromone pathway. Yeast. 2004;21(10):831–850. doi: 10.1002/yea.1122 15300679 10.1002/yea.1122

[11] Klipp E, Liebermeister W. Mathematical modeling of intracellular signaling pathways. BMC neuroscience. 2006;7(1):S10 doi: 10.1186/1471-2202-7-S1-S10 17118154 10.1186/1471-2202-7-S1-S10 PMC1775040

[12] Beguerisse-Díaz M, Desikan R, Barahona M. Linear models of activation cascades: analytical solutions and coarse-graining of delayed signal transduction. Journal of The Royal Society Interface. 2016;13(121):20160409 doi: 10.1098/rsif.2016.0409 10.1098/rsif.2016.0409 PMC5014067 27581482

[13] Kholodenko B, Hancock J, Kolch W. Signalling ballet in space and time. Nature reviews Molecular cell biology. 2010;11(6):414–426. doi: 10.1038/nrm2901 20495582 10.1038/nrm2901 PMC2977972

[14] Maeder CI, Hink MA, Kinkhabwala A, Mayr R, Bastiaens PIH, Knop M. Spatial regulation of Fus3 MAP kinase activity through a reaction-diffusion mechanism in yeast pheromone signalling. Nat Cell Biol. 2007;9(11):1319–26. doi: 10.1038/ncb1652 17952059 10.1038/ncb1652

[15] Dudin O, Merlini L, Martin SG. Spatial focalization of pheromone/MAPK signaling triggers commitment to cell–cell fusion. Genes & development. 2016;30(19):2226–2239. doi: 10.1101/gad.286922.116 27798845 10.1101/gad.286922.116 PMC5088570

[16] Neves SR, Tsokas P, Sarkar A, Grace EA, Rangamani P, Taubenfeld SM, et al Cell shape and negative links in regulatory motifs together control spatial information flow in signaling networks. Cell. 2008;133(4):666–680. doi: 10.1016/j.cell.2008.04.025 18485874 10.1016/j.cell.2008.04.025 PMC2728678

[17] Chay A, Zamparo I, Koschinski A, Zaccolo M, Blackwell KT. Control of *β*AR-and N-methyl-D-aspartate (NMDA) receptor-dependent cAMP dynamics in hippocampal neurons. PLoS Comput Biol. 2016;12(2):e1004735 doi: 10.1371/journal.pcbi.1004735 26901880 10.1371/journal.pcbi.1004735 PMC4763502

[18] Klünder B, Freisinger T, Wedlich-Söldner R, Frey E. GDI-mediated cell polarization in yeast provides precise spatial and temporal control of Cdc42 signaling. PLoS Comput Biol. 2013;9(12):e1003396 doi: 10.1371/journal.pcbi.1003396 24348237 10.1371/journal.pcbi.1003396 PMC3861033

[19] Elliott CM, Ranner T. Finite element analysis for a coupled bulk–surface partial differential equation. IMA Journal of Numerical Analysis. 2012; p. drs022.

[20] Eigel M, Müller R. A posteriori error control for stationary coupled bulk-surface equations. IMA Journal of Numerical Analysis. 2017; p. drw080.

[21] Levine H, Rappel WJ. Membrane-bound Turing patterns. Physical Review E. 2005;72(6):061912 doi: 10.1103/PhysRevE.72.061912 10.1103/PhysRevE.72.061912 16485979

[22] Rätz A, Röger M. Turing instabilities in a mathematical model for signaling networks. Journal of mathematical biology. 2012;65(6):1215–1244. 22127438 10.1007/s00285-011-0495-4

[23] Giese W, Eigel M, Westerheide S, Engwer C, Klipp E. Influence of cell shape, inhomogeneities and diffusion barriers in cell polarization models. Physical biology. 2015;12(6):066014 doi: 10.1088/1478-3975/12/6/066014 26599916 10.1088/1478-3975/12/6/066014

[24] Thalmeier D, Halatek J, Frey E. Geometry-induced protein pattern formation. Proceedings of the National Academy of Sciences. 2016;113(3):548–553. doi: 10.1073/pnas.1515191113 10.1073/pnas.1515191113 PMC4725492 26739566

[25] Berezhkovskii AM, Sample C, Shvartsman SY. How long does it take to establish a morphogen gradient? Biophysical journal. 2010;99(8):L59–L61. doi: 10.1016/j.bpj.2010.07.045 20959075 10.1016/j.bpj.2010.07.045 PMC2955507

[26] Klipp E, Nordlander B, Krüger R, Gennemark P, Hohmann S. Integrative model of the response of yeast to osmotic shock. Nature biotechnology. 2005;23(8):975–982. doi: 10.1038/nbt1114 16025103 10.1038/nbt1114

[27] Muzzey D, Gómez-Uribe CA, Mettetal JT, van Oudenaarden A. A systems-level analysis of perfect adaptation in yeast osmoregulation. Cell. 2009;138(1):160–171. doi: 10.1016/j.cell.2009.04.047 19596242 10.1016/j.cell.2009.04.047 PMC3109981

[28] Nardozzi JD, Lott K, Cingolani G. Phosphorylation meets nuclear import: a review. Cell Communication and Signaling. 2010;8(1):32 doi: 10.1186/1478-811X-8-32 21182795 10.1186/1478-811X-8-32 PMC3022542

[29] Marco E, Wedlich-Soldner R, Li R, Altschuler SJ, Wu LF. Endocytosis optimizes the dynamic localization of membrane proteins that regulate cortical polarity. Cell. 2007;129(2):411–422. doi: 10.1016/j.cell.2007.02.043 17448998 10.1016/j.cell.2007.02.043 PMC2000346

[30] Chou CS, Nie Q, Yi TM. Modeling robustness tradeoffs in yeast cell polarization induced by spatial gradients. PloS one. 2008;3(9):e3103 doi: 10.1371/journal.pone.0003103 21267054 10.1371/journal.pone.0003103 PMC3021495

[31] Okada S, Leda M, Hanna J, Savage NS, Bi E, Goryachev AB. Daughter cell identity emerges from the interplay of Cdc42, septins, and exocytosis. Developmental cell. 2013;26(2):148–161. doi: 10.1016/j.devcel.2013.06.015 23906065 10.1016/j.devcel.2013.06.015 PMC3730058

[32] Brown GC, Kholodenko BN. Spatial gradients of cellular phospho-proteins. FEBS letters. 1999;457(3):452–454. doi: 10.1016/S0014-5793(99)01058-3 10471827 10.1016/s0014-5793(99)01058-3

[33] Meyers J, Craig J, Odde DJ. Potential for control of signaling pathways via cell size and shape. Current biology. 2006;16(17):1685–1693. doi: 10.1016/j.cub.2006.07.056 16950104 10.1016/j.cub.2006.07.056

[34] Elowitz MB, Surette MG, Wolf PE, Stock JB, Leibler S. Protein Mobility in the Cytoplasm ofEscherichia coli. Journal of bacteriology. 1999;181(1):197–203. 9864330 10.1128/jb.181.1.197-203.1999 PMC103549

[35] Elf J, Li GW, Xie XS. Probing transcription factor dynamics at the single-molecule level in a living cell. Science. 2007;316(5828):1191–1194. doi: 10.1126/science.1141967 17525339 10.1126/science.1141967 PMC2853898

[36] Takahashi K, Tănase-Nicola S, Ten Wolde PR. Spatio-temporal correlations can drastically change the response of a MAPK pathway. Proceedings of the National Academy of Sciences. 2010;107(6):2473–2478. doi: 10.1073/pnas.0906885107 10.1073/pnas.0906885107 PMC2811204 20133748

[37] Abramowitz M, Stegun IA. Handbook of mathematical functions: with formulas, graphs, and mathematical tables. vol. 55 Courier Corporation; 1964.

[38] Kholodenko BN, Birtwistle MR. Four-dimensional dynamics of MAPK information-processing systems. Wiley Interdisciplinary Reviews: Systems Biology and Medicine. 2009;1(1):28–44. doi: 10.1002/wsbm.16 20182652 10.1002/wsbm.16 PMC2826817

[39] Hilioti Z, Sabbagh W, Paliwal S, Bergmann A, Goncalves MD, Bardwell L, et al Oscillatory phosphorylation of yeast Fus3 MAP kinase controls periodic gene expression and morphogenesis. Curr Biol. 2008;18(21):1700–6. doi: 10.1016/j.cub.2008.09.027 18976914 10.1016/j.cub.2008.09.027 PMC2602854

[40] Diener C, Schreiber G, Giese W, del Rio G, Schröder A, Klipp E. Yeast Mating and Image-Based Quantification of Spatial Pattern Formation. PLoS Computational Biology. 2014;10(6). doi: 10.1371/journal.pcbi.1003690 24967739 10.1371/journal.pcbi.1003690 PMC4072512

[41] Stelling J, Kholodenko BN. Signaling cascades as cellular devices for spatial computations. Journal of mathematical biology. 2009;58(1-2):35–55. doi: 10.1007/s00285-008-0162-6 18283462 10.1007/s00285-008-0162-6 PMC2574718

[42] Muñoz-García J, Neufeld Z, Kholodenko BN. Positional information generated by spatially distributed signaling cascades. PLoS Comput Biol. 2009;5(3):e1000330 doi: 10.1371/journal.pcbi.1000330 19300504 10.1371/journal.pcbi.1000330 PMC2654021

[43] Groen FC, Young IT, Ligthart G. A comparison of different focus functions for use in autofocus algorithms. Cytometry Part A. 1985;6(2):81–91. doi: 10.1002/cyto.990060202 10.1002/cyto.990060202 3979220

[44] Sun Y, Duthaler S, Nelson BJ. Autofocusing in computer microscopy: selecting the optimal focus algorithm. Microscopy research and technique. 2004;65(3):139–149. doi: 10.1002/jemt.20118 15605419 10.1002/jemt.20118

[45] Yeo T, Ong S, Sinniah R, et al Autofocusing for tissue microscopy. Image and vision computing. 1993;11(10):629–639. doi: 10.1016/0262-8856(93)90059-P

[46] Gilbarg D, Trudinger NS. Elliptic partial differential equations of second order. Springer; 2001.

[47] Kaimachnikov NP, Kholodenko BN. Toggle switches, pulses and oscillations are intrinsic properties of the Src activation/deactivation cycle. The FEBS journal. 2009;276(15):4102–4118. doi: 10.1111/j.1742-4658.2009.07117.x 19627364 10.1111/j.1742-4658.2009.07117.x PMC2924194

[48] Berezhkovskii AM, Sample C, Shvartsman SY. Formation of morphogen gradients: Local accumulation time. Physical Review E. 2011;83(5):051906 doi: 10.1103/PhysRevE.83.051906 10.1103/PhysRevE.83.051906 PMC4957404 21728570

[49] Ellery AJ, Simpson MJ, McCue SW, Baker RE. Simplified approach for calculating moments of action for linear reaction-diffusion equations. Physical Review E. 2013;88(5):054102 doi: 10.1103/PhysRevE.88.054102 10.1103/PhysRevE.88.054102 24329386

[50] Simpson MJ, Ellery AJ, McCue SW, Baker RE. Critical timescales and time intervals for coupled linear processes. The ANZIAM Journal. 2013;54(03):127–142. doi: 10.21914/anziamj.v54i0.6242

[51] Moore TI, Chou CS, Nie Q, Jeon NL, Yi TM. Robust spatial sensing of mating pheromone gradients by yeast cells. PloS one. 2008;3(12):e3865 doi: 10.1371/journal.pone.0003865 19052645 10.1371/journal.pone.0003865 PMC2586657

[52] Peletier MA, Westerhoff HV, Kholodenko BN. Control of spatially heterogeneous and time-varying cellular reaction networks: a new summation law. Journal of Theoretical biology. 2003;225(4):477–487. doi: 10.1016/S0022-5193(03)00289-3 14615206 10.1016/s0022-5193(03)00289-3

[53] Merlini L, Dudin O, Martin SG. Mate and fuse: how yeast cells do it. Open biology. 2013;3(3):130008 doi: 10.1098/rsob.130008 23466674 10.1098/rsob.130008 PMC3718343

[54] Grebenkov DS, Nguyen BT. Geometrical structure of Laplacian eigenfunctions. SIAM Review. 2013;55(4):601–667. doi: 10.1137/120880173

[55] Thomson TM, Benjamin KR, Bush A, Love T, Pincus D, Resnekov O, et al Scaffold number in yeast signaling system sets tradeoff between system output and dynamic range. Proc Natl Acad Sci USA. 2011;108(50):20265–70. doi: 10.1073/pnas.1004042108 22114196 10.1073/pnas.1004042108 PMC3250143

[56] Ventura AC, Bush A, Vasen G, Goldín MA, Burkinshaw B, Bhattacharjee N, et al Utilization of extracellular information before ligand-receptor binding reaches equilibrium expands and shifts the input dynamic range. Proceedings of the National Academy of Sciences. 2014;111(37):E3860–E3869. doi: 10.1073/pnas.1322761111 10.1073/pnas.1322761111 PMC4169960 25172920

[57] Foteinopoulos P, Mulder BM. A microtubule-based minimal model for spontaneous and persistent spherical cell polarity. PloS one. 2017;12(9):e0184706 doi: 10.1371/journal.pone.0184706 28931032 10.1371/journal.pone.0184706 PMC5607169

[58] Macia J, Regot S, Peeters T, Conde N, Sole R, Posas F. Dynamic signaling in the Hog1 MAPK pathway relies on high basal signal transduction. Sci Signal. 2009;2(63):ra13–ra13. doi: 10.1126/scisignal.2000056 19318625 10.1126/scisignal.2000056

[59] Fritsche-Guenther R, Witzel F, Sieber A, Herr R, Schmidt N, Braun S, et al Strong negative feedback from Erk to Raf confers robustness to MAPK signalling. Molecular systems biology. 2011;7(1):489 doi: 10.1038/msb.2011.27 21613978 10.1038/msb.2011.27 PMC3130559

[60] Schaber J, Baltanas R, Bush A, Klipp E, Colman-Lerner A. Modelling reveals novel roles of two parallel signalling pathways and homeostatic feedbacks in yeast. Molecular systems biology. 2012;8(1):622 doi: 10.1038/msb.2012.53 23149687 10.1038/msb.2012.53 PMC3531907

[61] Baum K, Politi AZ, Kofahl B, Steuer R, Wolf J. Feedback, mass conservation and reaction kinetics impact the robustness of cellular oscillations. PLoS computational biology. 2016;12(12):e1005298 doi: 10.1371/journal.pcbi.1005298 28027301 10.1371/journal.pcbi.1005298 PMC5226835

[62] Goodwin BC. Oscillatory behavior in enzymatic control processes. Advances in Enzyme Regulation. 1965;3:425–437. doi: 10.1016/0065-2571(65)90067-1 5861813 10.1016/0065-2571(65)90067-1

[63] Ananthasubramaniam B, Herzel H. Positive feedback promotes oscillations in negative feedback loops. PLoS One. 2014;9(8):e104761 doi: 10.1371/journal.pone.0104761 25126951 10.1371/journal.pone.0104761 PMC4134231

[64] Tyson JJ. Biochemical oscillations In: Computational cell biology: An Introductory Text on Computer Modeling in Molecular and Cell Biology. Springer-Verlag, New York; 2002 p. 230–260.

[65] Legewie S, Herzel H, Westerhoff HV, Blüthgen N. Recurrent design patterns in the feedback regulation of the mammalian signalling network. Molecular systems biology. 2008;4(1):190 doi: 10.1038/msb.2008.29 18463614 10.1038/msb.2008.29 PMC2424294

[66] Jilkine A, Marée AF, Edelstein-Keshet L. Mathematical model for spatial segregation of the Rho-family GTPases based on inhibitory crosstalk. Bulletin of mathematical biology. 2007;69(6):1943–1978. doi: 10.1007/s11538-007-9200-6 17457653 10.1007/s11538-007-9200-6

[67] Ferrell JE, Machleder EM. The biochemical basis of an all-or-none cell fate switch in Xenopus oocytes. Science. 1998;280(5365):895–898. doi: 10.1126/science.280.5365.895 9572732 10.1126/science.280.5365.895

[68] Conlon P, Gelin-Licht R, Ganesan A, Zhang J, Levchenko A. Single-cell dynamics and variability of MAPK activity in a yeast differentiation pathway. Proceedings of the National Academy of Sciences. 2016; p. 201610081. 10.1073/pnas.1610081113 PMC5056058 27651485

[69] Banderas A, Koltai M, Anders A, Sourjik V. Sensory input attenuation allows predictive sexual response in yeast. Nature Communications. 2016;7 doi: 10.1038/ncomms12590 27557894 10.1038/ncomms12590 PMC5007329

[70] Wartlick O, Kicheva A, Gonzalez-Gaitan M. Morphogen gradient formation. Cold Spring Harbor perspectives in biology. 2009;1(3):a001255 doi: 10.1101/cshperspect.a001255 20066104 10.1101/cshperspect.a001255 PMC2773637

[71] Kholodenko BN. Negative feedback and ultrasensitivity can bring about oscillations in the mitogen-activated protein kinase cascades. European Journal of Biochemistry. 2000;267(6):1583–1588. doi: 10.1046/j.1432-1327.2000.01197.x 10712587 10.1046/j.1432-1327.2000.01197.x

[72] Gordon PV, Sample C, Berezhkovskii AM, Muratov CB, Shvartsman SY. Local kinetics of morphogen gradients. Proceedings of the National Academy of Sciences. 2011;108(15):6157–6162. doi: 10.1073/pnas.1019245108 10.1073/pnas.1019245108 PMC3076853 21444770

[73] Halatek J, Frey E. Highly canalized MinD transfer and MinE sequestration explain the origin of robust MinCDE-protein dynamics. Cell Reports. 2012;1(6):741–752. doi: 10.1016/j.celrep.2012.04.005 22813748 10.1016/j.celrep.2012.04.005

[74] Wu F, Halatek J, Reiter M, Kingma E, Frey E, Dekker C. Multistability and dynamic transitions of intracellular Min protein patterns. Molecular systems biology. 2016;12(6):873 doi: 10.15252/msb.20156724 27279643 10.15252/msb.20156724 PMC4923923

[75] Orlandini E, Marenduzzo D, Goryachev A. Domain formation on curved membranes: phase separation or Turing patterns? Soft Matter. 2013;9(39):9311–9318. doi: 10.1039/c3sm50650a

[76] Chen W, Nie Q, Yi TM, Chou CS. Modelling of Yeast Mating Reveals Robustness Strategies for Cell-Cell Interactions. PLoS computational biology. 2016;12(7):e1004988 doi: 10.1371/journal.pcbi.1004988 27404800 10.1371/journal.pcbi.1004988 PMC4942089

[77] Giese W. The choreography of yeast mating. Humboldt-Universität zu Berlin, Lebenswissenschaftliche Fakultät; 2016.

[78] Rubinstein B, Slaughter BD, Li R. Weakly nonlinear analysis of symmetry breaking in cell polarity models. Physical biology. 2012;9(4):045006 doi: 10.1088/1478-3975/9/4/045006 22871896 10.1088/1478-3975/9/4/045006 PMC3437610

[79] Edelstein-Keshet L, Holmes WR, Zajac M, Dutot M. From simple to detailed models for cell polarization. Philosophical Transactions of the Royal Society of London B: Biological Sciences. 2013;368(1629):20130003 doi: 10.1098/rstb.2013.0003 24062577 10.1098/rstb.2013.0003 PMC3785957

[80] Rätz A, Röger M. Symmetry breaking in a bulk–surface reaction–diffusion model for signalling networks. Nonlinearity. 2014;27(8):1805 doi: 10.1088/0951-7715/27/8/1805

[81] Garcke H, Kampmann J, Rätz A, Röger M. A coupled surface-Cahn–Hilliard bulk-diffusion system modeling lipid raft formation in cell membranes. Mathematical Models and Methods in Applied Sciences. 2016;26(06):1149–1189. doi: 10.1142/S0218202516500275

[82] Madzvamuse A, Ndakwo HS, Barreira R. Stability analysis of reaction-diffusion models on evolving domains: the effects of cross-diffusion. Discrete and Continuous Dynamical Systems-Series A. 2016;36(4):2133–2170. doi: 10.3934/dcds.2016.36.2133

[83] Alnæs MS, Blechta J, Hake J, Johansson A, Kehlet B, Logg A, et al The FEniCS Project Version 1.5. Archive of Numerical Software. 2015;3(100).

[84] Logg A, Mardal KA, Wells GN, et al Automated Solution of Differential Equations by the Finite Element Method. Springer; 2012.

[85] The CGAL Project. CGAL User and Reference Manual. 4.11 ed CGAL Editorial Board; 2017 Available from: http://doc.cgal.org/4.11/Manual/packages.html.

