# Supplementary material for: Spatial modeling of the membrane-cytosolic interface in protein kinase signal transduction
Source: PLoS Comput Biol. 2018 Apr 9;14(4):e1006075. doi: 10.1371/journal.pcbi.1006075 (PMC5908195; doi:10.1371/journal.pcbi.1006075)
Supplement: S1 Appendix — The correspondence of the homogeneous ordinary differential equation system to the spatial MMC and PC system is established. Furthermore, an analytical expression for accumulation time of the MMC cascade is derived. (PDF) [file pcbi.1006075.s001.pdf]

Supporting information: Spatial modeling of the membrane-cytosolic interface in protein kinase signal transduction

Wolfgang Giese, Gregor Milicic, Andreas Schröder, Edda Klipp*

*corresponding author: edda.klipp@rz.hu-berlin.de

Contents

1 Model equations **2**

2 Linear signaling cascade on the membrane **2**

3 The membrane cytosolic interface **3**
3.1 Spatially homogeneous signal 3
3.2 Spatially heterogeneous signal 5
3.3 Special case: Solution without nucleus for the mixed membrane-cytosolic (MMC) cascade 7

4 Linear cascades in the cytosol **8**
4.1 Spatially homogeneous signal 8
4.2 Special case: Solution without nucleus for the pure cytosolic (PC) cascade 11
4.3 Spatially heterogeneous signals 12
4.4 Special case: Linear gradient for the pure cytosolic (PC) cascade 15
4.4.1 Without nucleus 15
4.4.2 With nucleus 18

5 Comparison of the spatial model with the assumption of a well-mixed compartment **21**
5.1 ODE system for the average concentration in the case $\gamma = 0$ 21

6 Estimates for the maximum, minimum and average concentration levels **22**
6.1 Lower bound for the maximal concentration 22
6.2 Upper bound for the minimal concentration 22
6.3 Upper bound for average concentration 23

7 Estimates for concentration differences in the signaling cascade **23**

8 Time scaling **25**

9 SI Figures **27**

SI Appendix

This appendix contains analytical solutions, estimates and boundaries for the spatial signal cascade models described in the main text. In particular, the general analytical solution for a cascade with N components, where the first M components are membrane-bound while the remaining $N-M$ components diffuse in the cytosol, is derived in Sections 2 - 4. To ease the access for the reader, the solutions for the three-component system (PC) and (MMC), which are discussed in the main text, are given in separate subsections. These analytical solutions for the three-component system were implemented using the programming language Python. The time-dependent linear and non-linear model of the three-component signaling cascade were implemented in the Python-based finite element framework FEniCS [2, 8]. A

correspondence of the spatial cascade model with a description of the average concentration by ordinary differential equations is established in Section 5 for arbitrary cell shapes. Furthermore, boundaries and estimates for the components of the spatial signaling cascade are derived for arbitrary cell shapes in Section 7. In the last Section 8, an analytical expression of the accumulation time for the MMC cascade is derived.

1 Model equations

We start with a linear signaling cascade with different localizations of the membrane-cytosolic interface as explained in the main text (also compare Fig 1, main text). A simple cascade model from [7], in which stimulation of a receptor leads to the consecutive activation of several down stream protein kinases is extended into space in the following. We assume a linear cascade with N components, where the first $M < N$ components are attached to the membrane while the remaining $N - M$ components are assumed to freely diffuse in the cytosol. The equations for the membrane-bound components read

$$\frac{\partial P_n}{\partial t} = D_{\text{mem}} \Delta_\Gamma P_n + \alpha_n P_{n-1} - \beta_n P_n \qquad \text{on the membrane}, \quad n = 1, \dots, M, \tag{1}$$

where P_0 is the input signal. For the modeling of the membrane-cytosolic interface, we include diffusion in the cytosol and reactions on its boundaries, which are the membranes. These processes are modeled by a reaction-diffusion equation with a robin boundary condition

$$\frac{\partial P_{M+1}}{\partial t} = D_{\text{cyt}} \Delta P_{M+1} - \beta_{M+1} P_{M+1} \qquad \text{in the cytosol}, \tag{2}$$

$$-D_{\text{cyt}} \nabla P_{M+1} \cdot \mathbf{n} = \alpha_{M+1} P_M - \gamma P_{M+1} \qquad \text{on the cell membrane}. \tag{3}$$

For the flux on all other membrane enclosed organelles we assume a zero-flux condition

$$-D_{\text{cyt}} \nabla P_{M+1} \cdot \mathbf{n} = 0. \tag{4}$$

The equations for the cytosolic cascade read

$$\frac{\partial P_n}{\partial t} = D_{\text{cyt}} \Delta P_n + \alpha_n P_{n-1} - \beta_n P_n, \text{ in the cytosol}, \quad n = M+2, \dots, N. \tag{5}$$

For the downstream cytosolic components we assume zero-flux on the cell membrane

$$-D_{\text{cyt}} \nabla P_n \cdot \mathbf{n} = 0, \quad n = M+2, \dots, N, \tag{6}$$

and for the boundary condition on the nucleus we assume

$$-D_{\text{cyt}} \nabla P_n \cdot \mathbf{n} = 0, \quad n = M+2, \dots, N-1, \tag{7}$$

$$-D_{\text{cyt}} \nabla P_N \cdot \mathbf{n} = -\epsilon P_N, \tag{8}$$

where ϵ describes selective nuclear import of P_N. Here and in the following we associate time independent functions with a bar, meaning that $\bar{P}_i$ denotes the steady state.

2 Linear signaling cascade on the membrane

We assume that the signal can be represented as generalized Fourier series (also known as Laplace series)

$$P_0(\theta, \phi, t) = \sum_{l=0}^{\infty} \sum_{m=-l}^{l} A_{0,l}^m(t) Y_l^m(\theta, \phi), \tag{9}$$

$$A_{0,l}^m(t) = \int_0^{2\pi} \int_0^{\pi} P_0(\theta, \phi, t) Y_l^{m*}(\theta, \phi) \sin(\theta) \; d\theta d\phi, \tag{10}$$

where $Y_l^{m*} = (-1)^m Y_l^{-m}$ is the complex conjugate [9]. The spherical harmonics up to order $l = 3$ are illustrated in Fig A3. The index l denotes the degree of the spherical harmonics, where a larger l indicate a higher angular frequency on the surface of the sphere. The index m, which fulfills $-l \leq m \leq l$, describes the symmetry of the respective spherical harmonic. For instance, $m = 0$ indicates rotational symmetry around the z-Axis. We assume without loss of generality that the signal has the form

$$P_0(\theta,\phi,t) = A_{0,0}^0(t)Y_0^0 + A_{0,l}^m(t)Y_l^m(\theta,\phi).$$

For the membrane-bound species we use the ansatz

$$P_n(\theta,\phi,t) = A_{n,0}^0(t)Y_0^0 + A_{n,l}^m(t)Y_l^m(\theta,\phi), \quad n = 1,\ldots,M. \tag{11}$$

Inserting this ansatz into the equation system (1) gives

$$\frac{dA_{n,l}^m(t)}{dt}Y_l^m(\theta,\phi) = -l(l+1)\frac{D_{\text{mem}}}{R_{\text{cell}}^2}A_{n,l}^m(t)Y_l^m(\theta,\phi) + \alpha_n A_{n-1,l}^m(t)Y_l^m(\theta,\phi) - \beta_n A_{n,l}^m(t)Y_l^m(\theta,\phi) \tag{12}$$

for $n = 1,\ldots,M$. Canceling the $Y_l^m(\theta,\phi)$ term results in a set of ordinary differential equations:

$$\frac{dA_{n,l}^m(t)}{dt} = -l(l+1)\frac{D_{\text{mem}}}{R_{\text{cell}}^2}A_{n,l}^m(t) + \alpha_n A_{n-1,l}^m(t) - \beta_n A_{n,l}^m(t), \quad n = 1,\ldots,M. \tag{13}$$

Therefore, the set of surface partial-differential equations, see (1), becomes a set of ordinary-differential equations. We can also compute the steady state of these equations, which is given by

$$\bar{A}_{n,0}^0 = \frac{\alpha_n}{\beta_n}\bar{A}_{n-1,0}^0, \tag{14}$$

$$\bar{A}_{n,l}^m = \frac{\alpha_n}{\beta_n + l(l+1)\frac{D_{\text{mem}}}{R_{\text{cell}}^2}}\bar{A}_{n-1,l}^m, \quad n = 1,\ldots,M. \tag{15}$$

The the steady state level of the spatially homogeneous part of the signal changes by the factor of α_n/β_n from cascade level $n-1$ to n. However, the amplitudes of the spatially heterogeneous part of the signal are changed by a smaller factor $\frac{\alpha_n}{\beta_n + l(l+1)\frac{D_{\text{mem}}}{R_{\text{cell}}^2}} < \frac{\alpha_n}{\beta_n}$ from level $n-1$ to n. Therefore, the spatially heterogeneous part of the signal is balanced out in linear signaling cascades. For heterogeneities with multiple peaks ($l > 1$), this effect is more pronounced. However, D_{mem} is usually very slow and therefore $l(l+1)\frac{D_{\text{mem}}}{R_{\text{cell}}^2} \ll \beta_n$ for gradients ($l = 1$) or heterogeneities with a low number of peaks. Thus, gradients are usually well preserved for signaling cascades on the membrane.

3 The membrane cytosolic interface

In the previous section, we described signal propagation on the cell membrane and transformed the spatial reaction-diffusion equations into a system of ordinary differential equations using the ansatz of spherical harmonics. In the following we will describe the signal transduction into the cytoplasm, which involves reactions at the membrane-cytosolic interface. Since the derivation of an analytical steady state solution is slightly more intricate than in the previous section, we start with a spatially homogeneous signal and then continue with a spatially heterogeneous signal in the next step.

3.1 Spatially homogeneous signal

For the steady state of the cytosolic species P_{M+1}, we use the ansatz

$$\bar{P}_{M+1}(r) = A\frac{\exp(r\sqrt{\frac{\beta_{M+1}}{D_{\text{cyt}}}})}{r} + B\frac{\exp(-r\sqrt{\frac{\beta_{M+1}}{D_{\text{cyt}}}})}{r}. \tag{16}$$

Here, we assume $R_{\text{nuc}} > 0$ and therefore $r > 0$, the case of a cell without nucleus ($R_{\text{nuc}} = 0$) is treated in Section 3.3. In the case of a spatially homogeneous signal on the cell membrane, concentration changes

of P_{M+1} only occur in radial direction. The radial part of the Laplace operator in spherical coordinates is given by $\Delta_r = \frac{\partial^2}{\partial^2 r} + \frac{2}{r}\frac{\partial}{\partial r}$. Equation (2) is fulfilled, since we have the relation

$$\Delta_r \frac{\exp(\pm r\sqrt{\frac{\beta_{M+1}}{D_{\text{cyt}}}})}{r} = \frac{\beta_{M+1}}{D_{\text{cyt}}} \frac{\exp(\pm r\sqrt{\frac{\beta_{M+1}}{D_{\text{cyt}}}})}{r}.$$

For the determination of the coefficients A and B, we use the boundary conditions

$$D_{\text{cyt}} \frac{\partial}{\partial r} \bar{P}_{M+1}(r)\Big|_{r=R_{\text{cell}}} = \alpha_{M+1}\bar{P}_M - \gamma \bar{P}_{M+1}(R_{\text{cell}}), \qquad \text{on the cell membrane,} \tag{17}$$

$$-D_{\text{cyt}} \frac{\partial}{\partial r} \bar{P}_{M+1}(r)\Big|_{r=R_{\text{nuc}}} = -\epsilon \bar{P}_{M+1}(R_{\text{nuc}}), \qquad \text{at the nucleus.} \tag{18}$$

Note, that ϵ is assumed to be zero if P_{M+1} is not the last element in the signaling cascade. Inserting the ansatz (16) into the boundary conditions (17) - (18) results in the equation system

$$A D_{\text{cyt}} \frac{\exp(R_{\text{cell}}\sqrt{\frac{\beta_{M+1}}{D_{\text{cyt}}}})}{R_{\text{cell}}} \left[\frac{1}{R_{\text{cell}}} - \sqrt{\frac{\beta_{M+1}}{D_{\text{cyt}}}}\right] + B D_{\text{cyt}} \frac{\exp(-R_{\text{cell}}\sqrt{\frac{\beta_{M+1}}{D_{\text{cyt}}}})}{R_{\text{cell}}} \left[\frac{1}{R_{\text{cell}}} + \sqrt{\frac{\beta_{M+1}}{D_{\text{cyt}}}}\right]$$

$$= \alpha_{M+1}\bar{P}_M - \gamma A \frac{\exp(R_{\text{cell}}\sqrt{\frac{\beta_{M+1}}{D_{\text{cyt}}}})}{R_{\text{cell}}} - \gamma B \frac{\exp(-R_{\text{cell}}\sqrt{\frac{\beta_{M+1}}{D_{\text{cyt}}}})}{R_{\text{cell}}}, \tag{19}$$

$$- A D_{\text{cyt}} \frac{\exp(R_{\text{nuc}}\sqrt{\frac{\beta_{M+1}}{D_{\text{cyt}}}})}{R_{\text{nuc}}} \left[\frac{1}{R_{\text{nuc}}} - \sqrt{\frac{\beta_{M+1}}{D_{\text{cyt}}}}\right] - B D_{\text{cyt}} \frac{\exp(-R_{\text{nuc}}\sqrt{\frac{\beta_{M+1}}{D_{\text{cyt}}}})}{R_{\text{nuc}}} \left[\frac{1}{R_{\text{nuc}}} + \sqrt{\frac{\beta_{M+1}}{D_{\text{cyt}}}}\right]$$

$$= -\epsilon A \frac{\exp(R_{\text{nuc}}\sqrt{\frac{\beta_{M+1}}{D_{\text{cyt}}}})}{R_{\text{nuc}}} - \epsilon B \frac{\exp(-R_{\text{nuc}}\sqrt{\frac{\beta_{M+1}}{D_{\text{cyt}}}})}{R_{\text{nuc}}}. \tag{20}$$

Rearrangement of these equations results in a linear equation system for A and B:

$$\underbrace{A\,\frac{\exp(R_{\text{cell}}\sqrt{\frac{\beta_{M+1}}{D_{\text{cyt}}}})}{R_{\text{cell}}}\left[\frac{D_{\text{cyt}}}{R_{\text{cell}}} - \sqrt{D_{\text{cyt}}\beta_{M+1}} + \gamma\right]}_{\omega_{11}}$$

$$+ \underbrace{B\,\frac{\exp(-R_{\text{cell}}\sqrt{\frac{\beta_{M+1}}{D_{\text{cyt}}}})}{R_{\text{cell}}}\left[\frac{D_{\text{cyt}}}{R_{\text{cell}}} + \sqrt{D_{\text{cyt}}\beta_{M+1}} + \gamma\right]}_{\omega_{12}} = \underbrace{\alpha_{M+1}\bar{P}_{k-1}}_{\xi_1}, \tag{21}$$

$$\underbrace{A\,D_{\text{cyt}}\frac{\exp(R_{\text{nuc}}\sqrt{\frac{\beta_{M+1}}{D_{\text{cyt}}}})}{R_{\text{nuc}}}\left[\frac{D_{\text{cyt}}}{R_{\text{nuc}}} - \sqrt{D_{\text{cyt}}\beta_{M+1}} - \epsilon\right]}_{\omega_{21}}$$

$$+ \underbrace{B\,D_{\text{cyt}}\frac{\exp(-R_{\text{nuc}}\sqrt{\frac{\beta_{M+1}}{D_{\text{cyt}}}})}{R_{\text{nuc}}}\left[\frac{D_{\text{cyt}}}{R_{\text{nuc}}} + \sqrt{D_{\text{cyt}}\beta_{M+1}} - \epsilon\right]}_{\omega_{22}} = 0. \tag{22}$$

The solution for A and B is then given by

$$A = \frac{\omega_{22}\xi_1}{\omega_{11}\omega_{22} - \omega_{12}\omega_{21}},$$

$$B = \frac{-\omega_{21}\xi_1}{\omega_{11}\omega_{22} - \omega_{12}\omega_{21}}.$$

Note, that alternatively the equivalent ansatz

$$\bar{P}_{M+1}(r) = \tilde{A} i_0(r\sqrt{\frac{\beta_{M+1}}{D_{\text{cyt}}}}) + \tilde{B} k_0(r\sqrt{\frac{\beta_{M+1}}{D_{\text{cyt}}}}), \tag{23}$$

can be used. The modified spherical Bessel functions of the first and second kind are given by [1]

$$i_0(x) = \frac{\exp(x) - \exp(-x)}{x}, \tag{24}$$

$$k_0(x) = \frac{\exp(-x)}{x}. \tag{25}$$

Therefore, the coefficients $\hat{A}$ and $\hat{B}$ can be computed from

$$\hat{A} = A \tag{26}$$

$$\hat{B} = B - A. \tag{27}$$

3.2 Spatially heterogeneous signal

Without loss of generality, we assume that the spatially heterogeneous signal has the form

$$\bar{P}_0(\theta,\phi) = A^0_{0,0} + A^m_{0,l} Y^m_l(\theta,\phi),$$

where $Y^m_l(\theta,\phi)$ is a spherical harmonic function of degree l. The spatially homogeneous part of the steady state solution of P_{M+1} was derived in the previous section. The ansatz for the steady state solution of P_{M+1} with a heterogeneous signal reads

$$\begin{aligned}\bar{P}_{M+1}(r,\theta,\phi) =& A^0_{M+1,0} i_0(r\sqrt{\frac{\beta_{M+1}}{D_{\text{cyt}}}}) + B^0_{M+1,0} k_0(r\sqrt{\frac{\beta_{M+1}}{D_{\text{cyt}}}})) \\ &+ A^m_{M+1,l} i_l(r\sqrt{\frac{\beta_{M+1}}{D_{\text{cyt}}}}) Y^m_l(\theta,\phi) + B^m_{M+1,l} k_l(r\sqrt{\frac{\beta_{M+1}}{D_{\text{cyt}}}})) Y^m_l(\theta,\phi). \end{aligned} \tag{28}$$

Equation (2) is fulfilled , since we have the relation

$$\begin{aligned}\Delta_{\theta,\phi,r} i_l(r\sqrt{\frac{\beta_{M+1}}{D_{\text{cyt}}}}) Y^m_l(\theta,\phi) &= i_l(r\sqrt{\frac{\beta_{M+1}}{D_{\text{cyt}}}}) \Delta_{\theta,\phi} Y^m_l(\theta,\phi) + Y^m_l(\theta,\phi) \Delta_r i_l(r\sqrt{\frac{\beta_{M+1}}{D_{\text{cyt}}}}) \\ &= -\frac{l(l+1)}{r^2} i_l(r\sqrt{\frac{\beta_{M+1}}{D_{\text{cyt}}}}) Y^m_l(\theta,\phi) + Y^m_l(\theta,\phi) \left[\frac{\beta_{M+1}}{D_{\text{cyt}}} + \frac{l(l+1)}{r^2}\right] i_l(r\sqrt{\frac{\beta_{M+1}}{D_{\text{cyt}}}}) \\ &= \frac{\beta_{M+1}}{D_{\text{cyt}}} i_l(r\sqrt{\frac{\beta_{M+1}}{D_{\text{cyt}}}}) Y^m_l(\theta,\phi). \end{aligned}$$

Similarly, we obtain the relation

$$\Delta_{\theta,\phi,r} k_l(r\sqrt{\frac{\beta_{M+1}}{D_{\text{cyt}}}}) Y^m_l(\theta,\phi) = \frac{\beta_{M+1}}{D_{\text{cyt}}} k_l(r\sqrt{\frac{\beta_{M+1}}{D_{\text{cyt}}}}) Y^m_l(\theta,\phi).$$

As in the previous section, we set $A^0_{k,0} = \hat{A}$ and $B^0_{k,0} = \hat{B}$ for the spatially homogeneous part of the solution. For the determination of the coefficients $A^m_{k,l}$ and $B^m_{k,l}$, we insert the ansatz into the boundary conditions

$$D_{\text{cyt}} \frac{\partial}{\partial r} \bar{P}_{M+1}(\theta,\phi,r)\Big|_{r=R_{\text{cell}}} = \alpha_{M+1} \bar{P}_M(\theta,\phi,R_{\text{cell}}) - \gamma_{M+1} P_{M+1}(\theta,\phi,R_{\text{cell}}), \quad \text{on the cell membrane,} \tag{29}$$

$$-D_{\text{cyt}} \frac{\partial}{\partial r} \bar{P}_{M+1}(\theta,\phi,r)\Big|_{r=R_{\text{nuc}}} = -\epsilon \bar{P}_{M+1}(\theta,\phi,R_{\text{nuc}}), \quad \text{at the nucleus.} \tag{30}$$

Since the spatially homogeneous part already fulfills these two equations, we only consider the spatially heterogeneous part. We use the relationships (compare [1])

$$D_{\text{cyt}} \frac{\partial}{\partial r} i_l(r\sqrt{\frac{\beta_{M+1}}{D_{\text{cyt}}}}) Y_l^m(\theta,\phi,r)\Big|_{r=R} = \sqrt{D_{\text{cyt}}\beta_{M+1}} i_{l+1}(R\sqrt{\frac{\beta_{M+1}}{D_{\text{cyt}}}}) Y_l^m(\theta,\phi,r) + D_{\text{cyt}}\frac{l}{R} i_l(R\sqrt{\frac{\beta_{M+1}}{D_{\text{cyt}}}}) Y_l^m(\theta,\phi,r), \tag{31}$$

and

$$D_{\text{cyt}} \frac{\partial}{\partial r} k_l(r\sqrt{\frac{\beta_{M+1}}{D_{\text{cyt}}}}) Y_l^m(\theta,\phi,r)\Big|_{r=R} = -\sqrt{D_{\text{cyt}}\beta_{M+1}} k_{l+1}(R\sqrt{\frac{\beta_{M+1}}{D_{\text{cyt}}}}) Y_l^m(\theta,\phi,r) + D_{\text{cyt}}\frac{l}{R} k_l(R\sqrt{\frac{\beta_{M+1}}{D_{\text{cyt}}}}) Y_l^m(\theta,\phi,r), \tag{32}$$

where $R = R_{\text{cell}}$ or $R = R_{\text{nuc}}$. Thus, the boundary condition on the cell membrane becomes

$$\begin{aligned}
&D_{\text{cyt}} Y_l^m(\theta,\phi)\sqrt{\frac{\beta_{M+1}}{D_{\text{cyt}}}}\left(A^m_{M+1,l} i_{l+1}(R_{\text{cell}}\sqrt{\frac{\beta_{M+1}}{D_{\text{cyt}}}}) - B^m_{M+1,l} k_{l+1}(R_{\text{cell}}\sqrt{\frac{\beta_{M+1}}{D_{\text{cyt}}}})\right)\\
&+D_{\text{cyt}} Y_l^m(\theta,\phi)\frac{l}{R_{\text{cell}}}\left(A^m_{M+1,l} i_l(R_{\text{cell}}\sqrt{\frac{\beta_{M+1}}{D_{\text{cyt}}}}) + B^m_{M+1,l} k_l(R_{\text{cell}}\sqrt{\frac{\beta_{M+1}}{D_{\text{cyt}}}})\right)\\
&= \alpha_{M+1} A^m_{M,l} Y_l^m(\theta,\phi) - \gamma A^m_{M+1,l} i_l(R_{\text{cell}}\sqrt{\frac{\beta_{M+1}}{D_{\text{cyt}}}}) Y_l^m(\theta,\phi)\\
&- \gamma B^m_{M+1,l} k_l(R_{\text{cell}}\sqrt{\frac{\beta_{M+1}}{D_{\text{cyt}}}}) Y_l^m(\theta,\phi),
\end{aligned} \tag{33}$$

and for the boundary condtion at the nucleus we obtain

$$\begin{aligned}
&D_{\text{cyt}} Y_l^m(\theta,\phi)\sqrt{\frac{\beta_{M+1}}{D_{\text{cyt}}}}\left(A^m_{M+1,l} i_{l+1}(R_{\text{nuc}}\sqrt{\frac{\beta_{M+1}}{D_{\text{cyt}}}}) - B^m_{M+1,l} k_{l+1}(R_{\text{nuc}}\sqrt{\frac{\beta_{M+1}}{D_{\text{cyt}}}})\right)\\
&+D_{\text{cyt}} Y_l^m(\theta,\phi)\frac{l}{R_{\text{nuc}}}\left(A^m_{M+1,l} i_l(R_{\text{nuc}}\sqrt{\frac{\beta_{M+1}}{D_{\text{cyt}}}}) + B^m_{M+1,l} k_l(R_{\text{nuc}}\sqrt{\frac{\beta_{M+1}}{D_{\text{cyt}}}})\right)\\
&= \epsilon A^m_{M+1,l} i_l(R_{\text{nuc}}\sqrt{\frac{\beta_{M+1}}{D_{\text{cyt}}}}) Y_l^m(\theta,\phi) + \epsilon B^m_{M+1,l} k_l(R_{\text{nuc}}\sqrt{\frac{\beta_{M+1}}{D_{\text{cyt}}}}) Y_l^m(\theta,\phi).
\end{aligned} \tag{34}$$

Rearrangement and canceling of the term $Y_l^m(\theta,\phi)$ results in

$$\begin{aligned}
&A^m_{M+1,l}\underbrace{\left[\left(D_{\text{cyt}}\frac{l}{R_{\text{cell}}}+\gamma\right) i_l(R_{\text{cell}}\sqrt{\frac{\beta_{M+1}}{D_{\text{cyt}}}}) + \sqrt{D_{\text{cyt}}\beta_{M+1}} i_{l+1}(R_{\text{cell}}\sqrt{\frac{\beta_{M+1}}{D_{\text{cyt}}}})\right]}_{\omega_{11}}\\
&+B^m_{M+1,l}\underbrace{\left[\left(D_{\text{cyt}}\frac{l}{R_{\text{cell}}}+\gamma\right) k_l(R_{\text{cell}}\sqrt{\frac{\beta_{M+1}}{D_{\text{cyt}}}}) - \sqrt{D_{\text{cyt}}\beta_{M+1}} k_{l+1}(R_{\text{cell}}\sqrt{\frac{\beta_{M+1}}{D_{\text{cyt}}}})\right]}_{\omega_{12}}\\
&= \underbrace{\alpha_{M+1} A^m_{M,l}}_{\xi_1}, \qquad \text{on the cell membrane,}
\end{aligned} \tag{35}$$

and

$$A^m_{M+1,l}\underbrace{\left[\left(D_{\text{cyt}}\frac{l}{R_{\text{nuc}}}+\gamma\right)i_l(R_{\text{nuc}}\sqrt{\frac{\beta_{M+1}}{D_{\text{cyt}}}})+\sqrt{D_{\text{cyt}}\beta_{M+1}}i_{l+1}(R_{\text{nuc}}\sqrt{\frac{\beta_{M+1}}{D_{\text{cyt}}}})\right]}_{\omega_{21}}$$

$$+B^m_{M+1,l}\underbrace{\left[\left(D_{\text{cyt}}\frac{l}{R_{\text{nuc}}}+\gamma\right)k_l(R_{\text{nuc}}\sqrt{\frac{\beta_{M+1}}{D_{\text{cyt}}}})-\sqrt{D_{\text{cyt}}\beta_{M+1}}k_{l+1}(R_{\text{nuc}}\sqrt{\frac{\beta_{M+1}}{D_{\text{cyt}}}})\right]}_{\omega_{22}}$$

$$=0 \qquad \text{at the nucleus.} \tag{36}$$

The solution for $A^m_{M+1,l}$ and $B^m_{M+1,l}$ is then obtained from

$$A^m_{M+1,l}=\frac{\omega_{22}\xi_1}{\omega_{11}\omega_{22}-\omega_{12}\omega_{21}}, \tag{37}$$

$$B^m_{M+1,l}=\frac{-\omega_{21}\xi_1}{\omega_{11}\omega_{22}-\omega_{12}\omega_{21}}. \tag{38}$$

3.3 Special case: Solution without nucleus for the mixed membrane-cytosolic (MMC) cascade

In the case of the MMC as described in the main text, we have $N=3$ and $M=2$. Furthermore we assume that there is no nucleus in the cytosol, meaning that $R_{\text{nuc}}=0$. The signal and the first two cascade components are decomposed using spherical harmonics as in the previous section:

$$\bar{P}_0(\theta,\phi)=A^m_{0,l}Y^m_l(\theta,\phi)+A^0_{0,0}, \tag{39}$$

$$\bar{P}_1(\theta,\phi)=A^m_{1,l}Y^m_l(\theta,\phi)+A^0_{1,0}, \tag{40}$$

$$\bar{P}_2(\theta,\phi)=A^m_{2,l}Y^m_l(\theta,\phi)+A^0_{2,0}. \tag{41}$$

As in equation (15), the coefficients for spatially homogeneous part are given by

$$A^0_{1,0}=\frac{\alpha_1}{\beta_1}A^0_{0,0}, \tag{42}$$

$$A^0_{2,0}=\frac{\alpha_2}{\beta_2}A^0_{1,0}, \tag{43}$$

and for the spatially heterogeneous part

$$A^m_{1,l}=\frac{\alpha_1}{\beta_1+l(l+1)\frac{D_{\text{mem}}}{R^2_{\text{cell}}}}A^m_{0,l}, \tag{44}$$

$$A^m_{2,l}=\frac{\alpha_2}{\beta_2+l(l+1)\frac{D_{\text{mem}}}{R^2_{\text{cell}}}}A^m_{1,l}. \tag{45}$$

For the cytosolic species $\bar{P}_3$, we get for the coefficient of the spatially homogeneous part

$$A^0_{3,0}=\frac{\alpha_3A^0_{2,0}}{\sqrt{D_{\text{cyt}}\beta_3}i_1(R_{\text{cell}}\sqrt{\frac{\beta_3}{D_{\text{cyt}}}})+\gamma i_0(R_{\text{cell}}\sqrt{\frac{\beta_3}{D_{\text{cyt}}}})}. \tag{46}$$

and for the spatially heterogeneous part we obtain

$$A^m_{3,l}=\frac{\alpha_3A^m_{2,l}}{\sqrt{D_{\text{cyt}}\beta_3}i_{l+1}(R_{\text{cell}}\sqrt{\frac{\beta_3}{D_{\text{cyt}}}})+\left(\frac{D_{\text{cyt}}l}{R_{\text{cell}}}+\gamma\right)i_l(R_{\text{cell}}\sqrt{\frac{\beta_3}{D_{\text{cyt}}}})}. \tag{47}$$

Inserting the solution for $A^0_{1,0}, A^0_{2,0}$ and $A^m_{1,l}, A^m_{2,l}$ we find that the amplitudes depend on the signal in the following way

$$A^0_{3,0} = \frac{\alpha_1\alpha_2\alpha_3}{\beta_1\beta_2} \frac{A^0_{0,0}}{\sqrt{D_{\text{cyt}}\beta_3} i_1(R_{\text{cell}}\sqrt{\frac{\beta_3}{D_{\text{cyt}}}}) + \gamma i_0(R_{\text{cell}}\sqrt{\frac{\beta_3}{D_{\text{cyt}}}})}. \tag{48}$$

and for the spatially heterogeneous part

$$A^m_{3,l} = \frac{\alpha_1}{\left(\beta_1 + \frac{l(l+1)D_{\text{mem}}}{R^2_{\text{cell}}}\right)} \frac{\alpha_2}{\left(\beta_2 + \frac{l(l+1)D_{\text{mem}}}{R^2_{\text{cell}}}\right)} \frac{\alpha_3 A^m_{0,l}}{\sqrt{D_{\text{cyt}}\beta_3} i_{l+1}(R_{\text{cell}}\sqrt{\frac{\beta_3}{D_{\text{cyt}}}}) + \left(\frac{D_{\text{cyt}} l}{R_{\text{cell}}} + \gamma\right) i_l(R_{\text{cell}}\sqrt{\frac{\beta_3}{D_{\text{cyt}}}})}. \tag{49}$$

4 Linear cascades in the cytosol

Similarly to Section 3, we start with the assumption of a constant homogeneous signal P_0 on the cell membrane and derive an analytical solution for a cascade in the cytosol of arbitrary length for this case. Based on this solution we then derive an analytical solution which is induced from a constant but spatially heterogeneous signal P_0 on the cell membrane. These derivations are followed by providing the analytical solution of the PC cascade with three cytosolic elements with and without a nucleus in the cytoplasm explicitely.

4.1 Spatially homogeneous signal

The steady state solution for the first species P_{M+1} can be obtained as in the previous section. Therefore, we assume without loss of generality that $M = 0$ and $N > 0$. Furthermore, we assume $\beta = \beta_1 = \cdots = \beta_N$. The general ansatz for the solution of the n-th cascade level ($n \leq N$) of a cytosolic cascade is given by

$$\bar{P}_n(r) = \sum_{k=1}^{n} A_{n,k} r^{k-2} \exp(\sqrt{\frac{\beta}{D_{\text{cyt}}}} r) + \sum_{k=1}^{n} B_{n,k} r^{k-2} \exp(-\sqrt{\frac{\beta}{D_{\text{cyt}}}} r). \tag{50}$$

In the following, we will derive expressions for the coefficients $A_{n,k}$ and $B_{n,k}$. For the derivative of the summands we obtain

$$\frac{\partial}{\partial r} r^n \exp(\sqrt{\frac{\beta}{D_{\text{cyt}}}} r) = n r^{n-1} \exp(\sqrt{\frac{\beta}{D_{\text{cyt}}}} r) + \sqrt{\frac{\beta}{D_{\text{cyt}}}} r^n \exp(\sqrt{\frac{\beta}{D_{\text{cyt}}}} r),$$
$$\frac{\partial}{\partial r} r^n \exp(-\sqrt{\frac{\beta}{D_{\text{cyt}}}} r) = n r^{n-1} \exp(-\sqrt{\frac{\beta}{D_{\text{cyt}}}} r) - \sqrt{\frac{\beta}{D_{\text{cyt}}}} r^n \exp(-\sqrt{\frac{\beta}{D_{\text{cyt}}}} r).$$

Application of the Laplace operator results in

$$\begin{aligned}
&\Delta_r r^n \exp(\sqrt{\frac{\beta}{D_{\text{cyt}}}} r) \qquad (51)\\
=&\left(\frac{\partial^2}{\partial^2 r} + \frac{2}{r}\frac{\partial}{\partial r}\right) r^n \exp(\sqrt{\frac{\beta}{D_{\text{cyt}}}} r)\\
=&n(n-1) r^{n-2} \exp(\sqrt{\frac{\beta}{D_{\text{cyt}}}} r) + 2\sqrt{\frac{\beta}{D_{\text{cyt}}}} n r^{n-1} \exp(\sqrt{\frac{\beta}{D_{\text{cyt}}}} r) + \frac{\beta}{D_{\text{cyt}}} r^n \exp(\sqrt{\frac{\beta}{D_{\text{cyt}}}} r)\\
&+ 2n r^{n-2} \exp(\sqrt{\frac{\beta}{D_{\text{cyt}}}} r) + 2\sqrt{\frac{\beta}{D_{\text{cyt}}}} r^{n-1} \exp(\sqrt{\frac{\beta}{D_{\text{cyt}}}} r)\\
=&n(n+1) r^{n-2} \exp(\sqrt{\frac{\beta}{D_{\text{cyt}}}} r) + 2\sqrt{\frac{\beta}{D_{\text{cyt}}}} (n+1) r^{n-1} \exp(\sqrt{\frac{\beta}{D_{\text{cyt}}}} r) + \frac{\beta}{D_{\text{cyt}}} r^n \exp(\sqrt{\frac{\beta}{D_{\text{cyt}}}} r), \qquad (52)
\end{aligned}$$

and, similarly

$$
\begin{aligned}
&\Delta_r r^n \exp(-\sqrt{\frac{\beta}{D_{\text{cyt}}}}r) \\
=&n(n+1)r^{n-2}\exp(-\sqrt{\frac{\beta}{D_{\text{cyt}}}}r) - 2\sqrt{\frac{\beta}{D_{\text{cyt}}}}(n+1)r^{n-1}\exp(-\sqrt{\frac{\beta}{D_{\text{cyt}}}}r) + \frac{\beta}{D_{\text{cyt}}}r^n\exp(-\sqrt{\frac{\beta}{D_{\text{cyt}}}}r).
\end{aligned}
$$

Inserting the ansatz (50) into equation (5) yields

$$
\begin{aligned}
0 =& \sum_{k=1}^{n} D_{\text{cyt}}A_{n,k}\Delta_r r^{k-2}\exp(\sqrt{\frac{\beta}{D_{\text{cyt}}}}r) + \sum_{k=1}^{n} D_{\text{cyt}}B_{n,k}\Delta_r r^{k-2}\exp(-\sqrt{\frac{\beta}{D_{\text{cyt}}}}r) \\
&+ \alpha_n \sum_{k=1}^{n-1} A_{n-1,k}r^{k-2}\exp(\sqrt{\frac{\beta}{D_{\text{cyt}}}}r) + \alpha_n \sum_{k=1}^{n-1} B_{n-1,k}r^{k-2}\exp(-\sqrt{\frac{\beta}{D_{\text{cyt}}}}r) \\
&- \beta\sum_{k=1}^{n} A_{n,k}r^{k-2}\exp(\sqrt{\frac{\beta}{D_{\text{cyt}}}}r) - \beta\sum_{k=1}^{n} B_{n,k}r^{k-2}\exp(-\sqrt{\frac{\beta}{D_{\text{cyt}}}}r) \\
=& \sum_{k=1}^{n} D_{\text{cyt}}A_{n,k}\left((k-2)(k-1)r^{k-4} + 2\sqrt{\frac{\beta}{D_{\text{cyt}}}}(k-1)r^{k-3} + \frac{\beta}{D_{\text{cyt}}}r^{k-2}\right)\exp(\sqrt{\frac{\beta}{D_{\text{cyt}}}}r) \\
&+ \sum_{k=1}^{n} D_{\text{cyt}}B_{n,k}\left((k-2)(k-1)r^{k-4} - 2\sqrt{\frac{\beta}{D_{\text{cyt}}}}(k-1)r^{k-3} + \frac{\beta}{D_{\text{cyt}}}r^{k-2}\right)\exp(-\sqrt{\frac{\beta}{D_{\text{cyt}}}}r) \\
&+ \alpha_n \sum_{k=2}^{n} A_{n-1,k-1}r^{k-3}\exp(\sqrt{\frac{\beta}{D_{\text{cyt}}}}r) + \alpha_n \sum_{k=2}^{n} B_{n-1,k-1}r^{k-3}\exp(-\sqrt{\frac{\beta}{D_{\text{cyt}}}}r) \\
&- \beta\sum_{k=1}^{n} A_{n,k}r^{k-2}\exp(\sqrt{\frac{\beta}{D_{\text{cyt}}}}r) - \beta\sum_{k=1}^{n} B_{n,k}r^{k-2}\exp(-\sqrt{\frac{\beta}{D_{\text{cyt}}}}r) = (*).
\end{aligned}
$$

Canceling of the terms containing βr^{k-2} and further rearrangement of $(*)$ results in

$$
\begin{aligned}
(*) =& \left(\alpha_n A_{n-1,n-1} + 2A_{n,n}\sqrt{D_{\text{cyt}}\beta}(n-1)\right) r^{n-3}\exp(\sqrt{\frac{\beta}{D_{\text{cyt}}}}r) \\
&+ \sum_{k=2}^{n-1}\left[D_{\text{cyt}}A_{n,k+1}(k-1)k + \left(\alpha_n A_{n-1,k-1} + 2A_{n,k}\sqrt{D_{\text{cyt}}\beta}(k-1)\right)\right] r^{k-3}\exp(\sqrt{\frac{\beta}{D_{\text{cyt}}}}r) \\
&+ \left(\alpha_n B_{n-1,n-1} - 2B_{n,n}\sqrt{D_{\text{cyt}}\beta}(n-1)\right) r^{n-3}\exp(-\sqrt{\frac{\beta}{D_{\text{cyt}}}}r) \\
&+ \sum_{k=2}^{n}\left[D_{\text{cyt}}B_{n,k+1}(k-1)k + \left(\alpha_n B_{n-1,k-1} - 2B_{n,k}\sqrt{D_{\text{cyt}}\beta}(k-1)\right)\right] r^{k-3}\exp(-\sqrt{\frac{\beta}{D_{\text{cyt}}}}r).
\end{aligned}
$$

Equating the coefficients results in the relations

$$
A_{n,n} = -\frac{\alpha_n A_{n-1,n-1}}{2(n-1)\sqrt{D_{\text{cyt}}\beta}} \tag{53}
$$

and

$$
B_{n,n} = \frac{\alpha_n B_{n-1,n-1}}{2(n-1)\sqrt{D_{\text{cyt}}\beta}}, \tag{54}
$$

which directly give the coefficients $A_{n,n}$ and $B_{n,n}$. Furthermore, we obtain the recursive formulars

$$
A_{n,k} = -\frac{\alpha_n A_{n-1,k-1} + D_{\text{cyt}}(k-1)kA_{n,k+1}}{2(k-1)\sqrt{D_{\text{cyt}}\beta}} \text{ for } 2 \le k \le n-1, \tag{55}
$$

and

$$B_{n,k} = \frac{\alpha_n B_{n-1,k-1} + D_{\rm cyt}(k-1)kB_{n,k+1}}{2(k-1)\sqrt{D_{\rm cyt}\beta}} \text{ for } 2 \le k \le n-1. \tag{56}$$

which can be used to calculate the coefficients $A_{n,k}$ and $B_{n,k}$ for $2 \le k \le n-1$. The remaining coefficients $A_{n,1}$ and $B_{n,1}$ are determined by the two boundary conditions on the cell membrane and nucleus. The diffusive flux of the species P_n is given by

$$\begin{aligned} D_{\rm cyt}\frac{\partial \bar{P}_n(r)}{\partial r} &= \sum_{k=1}^{n} D_{\rm cyt}A_{n,k}\frac{\partial}{\partial r}\left[r^{k-2}\exp(\sqrt{\frac{\beta}{D_{\rm cyt}}}r)\right] + \sum_{k=1}^{n} D_{\rm cyt}B_{n,k}\frac{\partial}{\partial r}\left[r^{k-2}\exp(-\sqrt{\frac{\beta}{D_{\rm cyt}}}r)\right] \\ &= \sum_{k=1}^{n} D_{\rm cyt}A_{n,k}\left[(k-2)r^{k-3} + \sqrt{\frac{\beta}{D_{\rm cyt}}}r^{k-2}\right]\exp(\sqrt{\frac{\beta}{D_{\rm cyt}}}r) \\ &+ \sum_{k=1}^{n} D_{\rm cyt}B_{n,k}\left[(k-2)r^{k-3} - \sqrt{\frac{\beta}{D_{\rm cyt}}}r^{k-2}\right]\exp(-\sqrt{\frac{\beta}{D_{\rm cyt}}}r). \end{aligned}$$

The boundary condition evaluated on the cell membrane reads

$$\begin{aligned} 0 &= D_{\rm cyt}\frac{\partial \bar{P}_n(r)}{\partial r}|_{R_{\rm cell}} \\ &= \sum_{k=1}^{n} D_{\rm cyt}A_{n,k}\left[(k-2)R_{\rm cell}^{k-3}\exp(\sqrt{\frac{\beta}{D_{\rm cyt}}}R_{\rm cell}) + \sqrt{\frac{\beta}{D_{\rm cyt}}}R_{\rm cell}^{k-2}\right]\exp(\sqrt{\frac{\beta}{D_{\rm cyt}}}R_{\rm cell}) \\ &+ \sum_{k=1}^{n} D_{\rm cyt}B_{n,k}\left[(k-2)R_{\rm cell}^{k-3}\exp(\sqrt{\frac{\beta}{D_{\rm cyt}}}R_{\rm cell}) - \sqrt{\frac{\beta}{D_{\rm cyt}}}R_{\rm cell}^{k-2}\right]\exp(-\sqrt{\frac{\beta}{D_{\rm cyt}}}R_{\rm cell}). \end{aligned}$$

Similarly, evaluation of the boundary condition at the nucleus results in

$$\begin{aligned} 0 &= D_{\rm cyt}\frac{\partial \bar{P}_n(r)}{\partial r}|_{R_{\rm nuc}} \\ &= \sum_{k=1}^{n} D_{\rm cyt}A_{n,k}\left[(k-2)R_{\rm nuc}^{k-3} + \sqrt{\frac{\beta}{D_{\rm cyt}}}R_{\rm nuc}^{k-2}\right]\exp(\sqrt{\frac{\beta}{D_{\rm cyt}}}R_{\rm nuc}) \\ &+ \sum_{k=1}^{n} D_{\rm cyt}B_{n,k}\left[(k-2)R_{\rm nuc}^{k-3} - \sqrt{\frac{\beta}{D_{\rm cyt}}}R_{\rm nuc}^{k-2}\right]\exp(-\sqrt{\frac{\beta}{D_{\rm cyt}}}R_{\rm nuc}). \end{aligned}$$

After rearrangement of these two equations, we obtain the linear equation system

$$\begin{aligned} &A_{n,1}D_{\rm cyt}\left[R_{\rm cell}^{-2} - \sqrt{\frac{\beta}{D_{\rm cyt}}}R_{\rm cell}^{-1}\right]\exp(\sqrt{\frac{\beta}{D_{\rm cyt}}}R_{\rm cell}) + B_{n,1}D_{\rm cyt}\left[R_{\rm cell}^{-2} + \sqrt{\frac{\beta}{D_{\rm cyt}}}R_{\rm cell}^{-1}\right]\exp(-\sqrt{\frac{\beta}{D_{\rm cyt}}}R_{\rm cell}) \\ &= \sum_{k=2}^{n} D_{\rm cyt}A_{n,k}\left[(k-2)R_{\rm cell}^{k-3} + \sqrt{\frac{\beta}{D_{\rm cyt}}}R_{\rm cell}^{k-2}\right]\exp(\sqrt{\frac{\beta}{D_{\rm cyt}}}R_{\rm cell}) \\ &+ \sum_{k=2}^{n} D_{\rm cyt}B_{n,k}\left[(k-2)R_{\rm cell}^{k-3} - \sqrt{\frac{\beta}{D_{\rm cyt}}}R_{\rm cell}^{k-2}\right]\exp(-\sqrt{\frac{\beta}{D_{\rm cyt}}}R_{\rm cell}), \qquad (57) \\ &A_{n,1}D_{\rm cyt}\left[R_{\rm nuc}^{-2} - \sqrt{\frac{\beta}{D_{\rm cyt}}}R_{\rm nuc}^{-1}\right]\exp(\sqrt{\frac{\beta}{D_{\rm cyt}}}R_{\rm nuc}) + B_{n,1}D_{\rm cyt}\left[R_{\rm nuc}^{-2} + \sqrt{\frac{\beta}{D_{\rm cyt}}}R_{\rm nuc}^{-1}\right]\exp(-\sqrt{\frac{\beta}{D_{\rm cyt}}}R_{\rm nuc}) \\ &= \sum_{k=2}^{n} D_{\rm cyt}A_{n,k}\left[(k-2)R_{\rm nuc}^{k-3} + \sqrt{\frac{\beta}{D_{\rm cyt}}}R_{\rm nuc}^{k-2}\right]\exp(\sqrt{\frac{\beta}{D_{\rm cyt}}}R_{\rm nuc}) \\ &+ \sum_{k=2}^{n} D_{\rm cyt}B_{n,k}\left[(k-2)R_{\rm nuc}^{k-3} - \sqrt{\frac{\beta}{D_{\rm cyt}}}R_{\rm nuc}^{k-2}\right]\exp(-\sqrt{\frac{\beta}{D_{\rm cyt}}}R_{\rm nuc}). \qquad (58) \end{aligned}$$

These two equations form a linear equation system, which can be solved explicitly for $A_{n,1}$ and $B_{n,1}$ in the same manner as in the previous sections.

4.2 Special case: Solution without nucleus for the pure cytosolic (PC) cascade

A steady state solution for the first cascade element is given by

$$\bar{P}_1 = A_1 i_0(\sqrt{\frac{\beta}{D_{\text{cyt}}}} r),$$
$$A_1 = \frac{\alpha_1 \bar{P}_0}{\sqrt{D_{\text{cyt}}\beta}\, i_1(\sqrt{\frac{\beta}{D_{\text{cyt}}}} R_{\text{cell}}) + \gamma i_0(\sqrt{\frac{\beta}{D_{\text{cyt}}}} R_{\text{cell}})}. \tag{59}$$

For the second cascade level we use the ansatz

$$\bar{P}_2(r) = A_2 i_0(\sqrt{\frac{\beta}{D_{\text{cyt}}}} r) + B_2 \cosh(\sqrt{\frac{\beta}{D_{\text{cyt}}}} r). \tag{60}$$

This yields

$$\begin{aligned}
&D_{\text{cyt}} \Delta_r \bar{P}_2(r) + \alpha_1 \bar{P}_1 - \beta \bar{P}_2(r) \\
&= D_{\text{cyt}} \left(\frac{\partial^2}{\partial^2 r} + \frac{2}{r} \frac{\partial}{\partial r} \right) B_2 \cosh(\sqrt{\frac{\beta}{D_{\text{cyt}}}} r) + \alpha_1 \bar{P}_1 - \beta B_2 \cosh(\sqrt{\frac{\beta}{D_{\text{cyt}}}} r) \\
&= \beta B_2 \cosh(\sqrt{\frac{\beta}{D_{\text{cyt}}}} r) + B_2 \frac{2}{r} \sqrt{D_{\text{cyt}}\beta} \sinh(\sqrt{\frac{\beta}{D_{\text{cyt}}}} r) + \alpha_1 A_1 i_0(\sqrt{\frac{\beta}{D_{\text{cyt}}}} r) - \beta B_2 \cosh(\sqrt{\frac{\beta}{D_{\text{cyt}}}} r) \\
&= 2 B_2 \beta i_0(\sqrt{\frac{\beta}{D_{\text{cyt}}}} r) + \alpha_1 A_1 i_0(\sqrt{\frac{\beta}{D_{\text{cyt}}}} r).
\end{aligned}$$

Equating the coefficients leads to the relations

$$0 = 2 B_2 \beta + \alpha_1 A_1, \tag{61}$$

which can be solved for B_2:

$$B_2 = -\frac{\alpha_1 A_1}{2\beta}. \tag{62}$$

The constant A_2 is determined from the boundary conditions

$$\begin{aligned}
0 &= D_{\text{cyt}} \left. \frac{\partial}{\partial r} \bar{P}_2(r) \right|_{r=R_{\text{cell}}} \\
&= D_{\text{cyt}} A_2 \sqrt{\frac{\beta}{D_{\text{cyt}}}} i_1(\sqrt{\frac{\beta}{D_{\text{cyt}}}} R_{\text{cell}}) + D_{\text{cyt}} B_2 \sqrt{\frac{\beta}{D_{\text{cyt}}}} \sinh(\sqrt{\frac{\beta}{D_{\text{cyt}}}} R_{\text{cell}}).
\end{aligned} \tag{63}$$

This yields the equation

$$A_2 = -\frac{B_2 \sinh(\sqrt{\frac{\beta}{D_{\text{cyt}}}} R_{\text{cell}})}{i_1(\sqrt{\frac{\beta}{D_{\text{cyt}}}} R_{\text{cell}})}. \tag{64}$$

The ansatz for the third cascade element reads

$$\bar{P}_3(r) = A_3 i_0(\sqrt{\frac{\beta}{D_{\text{cyt}}}} r) + B_3 \cosh(\sqrt{\frac{\beta}{D_{\text{cyt}}}} r) + C_3 r \sinh(\sqrt{\frac{\beta}{D_{\text{cyt}}}} r). \tag{65}$$

Application of the Laplace operator results in

$$
\begin{aligned}
&D_{\mathrm{cyt}}\Delta_r\bar{P}_3(r)+\alpha_3\bar{P}_2(r)-\beta\bar{P}_3(r)\\
=&2B_3\beta i_0(\sqrt{\frac{\beta}{D_{\mathrm{cyt}}}}r)+C_3D_{\mathrm{cyt}}\left(\frac{\partial^2}{\partial^2 r}+\frac{2}{r}\frac{\partial}{\partial r}\right)r\sinh(\sqrt{\frac{\beta}{D_{\mathrm{cyt}}}}r)+\alpha_3\bar{P}_2(r)-\beta C_3 r\sinh(\sqrt{\frac{\beta}{D_{\mathrm{cyt}}}}r)\\
=&2B_3\beta i_0(\sqrt{\frac{\beta}{D_{\mathrm{cyt}}}}r)+C_3D_{\mathrm{cyt}}\Bigg(2\sqrt{\frac{\beta}{D_{\mathrm{cyt}}}}\cosh(\sqrt{\frac{\beta}{D_{\mathrm{cyt}}}}r)+r\frac{\beta}{D_{\mathrm{cyt}}}\sinh(\sqrt{\frac{\beta}{D_{\mathrm{cyt}}}}r)+\frac{2}{r}\sinh(\sqrt{\frac{\beta}{D_{\mathrm{cyt}}}}r)\\
&+2\sqrt{\frac{\beta}{D_{\mathrm{cyt}}}}\cosh(\sqrt{\frac{\beta}{D_{\mathrm{cyt}}}}r)\Bigg)+\alpha_3\bar{P}_2(r)-\beta C_3 r\sinh(\sqrt{\frac{\beta}{D_{\mathrm{cyt}}}}r)\\
=&(\alpha_3A_2+2B_3\beta+2C_3\sqrt{D_{\mathrm{cyt}}\beta})i_0(\sqrt{\frac{\beta}{D_{\mathrm{cyt}}}}r)+(\alpha_3B_2+4C_3\sqrt{D_{\mathrm{cyt}}\beta})\cosh(\sqrt{\frac{\beta}{D_{\mathrm{cyt}}}}r).
\end{aligned}
$$

This leads to the conditions

$$
C_3=-\frac{\alpha_3B_2}{4\sqrt{D_{\mathrm{cyt}}\beta}}, \tag{66}
$$

$$
B_3=-\frac{\alpha_3A_2+2C_3\sqrt{D_{\mathrm{cyt}}\beta}}{2\beta}. \tag{67}
$$

For the boundary condition on the cell membrane, we get

$$
\begin{aligned}
0=&D_{\mathrm{cyt}}\frac{\partial}{\partial r}\bar{P}_3(r)|_{r=R_{\mathrm{cell}}}\\
=&D_{\mathrm{cyt}}A_3\sqrt{\frac{\beta}{D_{\mathrm{cyt}}}}i_1(\sqrt{\frac{\beta}{D_{\mathrm{cyt}}}}R_{\mathrm{cell}})+D_{\mathrm{cyt}}B_3\sqrt{\frac{\beta}{D_{\mathrm{cyt}}}}\sinh(\sqrt{\frac{\beta}{D_{\mathrm{cyt}}}}R_{\mathrm{cell}})\\
&+D_{\mathrm{cyt}}C_3\sinh(\sqrt{\frac{\beta}{D_{\mathrm{cyt}}}}R_{\mathrm{cell}})+D_{\mathrm{cyt}}C_3R_{\mathrm{cell}}\sqrt{\frac{\beta}{D_{\mathrm{cyt}}}}\cosh(\sqrt{\frac{\beta}{D_{\mathrm{cyt}}}}R_{\mathrm{cell}}),
\end{aligned}
$$

which results in

$$
A_3=-B_3\frac{\sinh(\sqrt{\frac{\beta}{D_{\mathrm{cyt}}}}R_{\mathrm{cell}})}{i_1(\sqrt{\frac{\beta}{D_{\mathrm{cyt}}}}R_{\mathrm{cell}})}-C_3\frac{\sqrt{\frac{D_{\mathrm{cyt}}}{\beta}}\sinh(\sqrt{\frac{\beta}{D_{\mathrm{cyt}}}}R_{\mathrm{cell}})+R_{\mathrm{cell}}\cosh(\sqrt{\frac{\beta}{D_{\mathrm{cyt}}}}R_{\mathrm{cell}})}{i_1(\sqrt{\frac{\beta}{D_{\mathrm{cyt}}}}R_{\mathrm{cell}})}. \tag{68}
$$

4.3 Spatially heterogeneous signals

Without loss of generality, we assume a spatially heterogeneous signal of the form

$$
\bar{P}_0(\theta,\phi)=A^0_{0,0}+A^m_{0,l}Y^m_l(\theta,\phi).
$$

The ansatz for a cytosolic cascade with a spatially heterogeneous signal reads

$$
\begin{aligned}
\bar{P}_n(r)=&\sum_{k=0}^{n-1}A_{n,k}r^{k-1}\exp(\sqrt{\frac{\beta}{D_{\mathrm{cyt}}}}r)+\sum_{k=0}^{n-1}B_{n,k}r^{k-1}\exp(-\sqrt{\frac{\beta}{D_{\mathrm{cyt}}}}r)\\
&+Y^m_l(\theta,\phi)\sum_{k=-l}^{n-1}A^m_{n,k,l}r^{k-1}\exp(\sqrt{\frac{\beta}{D_{\mathrm{cyt}}}}r)+Y^m_l(\theta,\phi)\sum_{k=-l}^{n-1}B^m_{n,k,l}r^{k-1}\exp(-\sqrt{\frac{\beta}{D_{\mathrm{cyt}}}}r),
\end{aligned} \tag{69}
$$

where $n\le N$ and we assume $M=0$. We already know that the spatially homogeneous part of the ansatz (69) satisfies equation (5). Applying the spherical Laplacian to the spatially heterogeneities part of (69)

results in

$$
\begin{aligned}
0 =& D_{\text{cyt}}\Delta_{\theta,\phi,r}Y_l^m(\theta,\phi)\sum_{k=-l}^{n-1}A_{n,k,l}^m r^{k-1}\exp(\sqrt{\frac{\beta}{D_{\text{cyt}}}}r) + \alpha_n Y_l^m(\theta,\phi)\sum_{k=-l}^{n-2}A_{n-1,k,l}^m r^{k-1}\exp(\sqrt{\frac{\beta}{D_{\text{cyt}}}}r)\\
&- \beta Y_l^m(\theta,\phi)\sum_{k=-l}^{n-1}A_{n,k,l}^m r^{k-1}\exp(\sqrt{\frac{\beta}{D_{\text{cyt}}}}r)\\
=& - D_{\text{cyt}}\frac{l(l+1)}{r^2}Y_l^m(\theta,\phi)\sum_{k=-l}^{n-1}A_{n,k,l}^m r^{k-1}\exp(\sqrt{\frac{\beta}{D_{\text{cyt}}}}r) + D_{\text{cyt}}Y_l^m(\theta,\phi)\Delta_r\sum_{k=-l}^{n-1}A_{n,k,l}^m r^{k-1}\exp(\sqrt{\frac{\beta}{D_{\text{cyt}}}}r)\\
&+ \alpha_n Y_l^m(\theta,\phi)\sum_{k=-l}^{n-2}A_{n-1,k,l}^m r^{k-1}\exp(\sqrt{\frac{\beta}{D_{\text{cyt}}}}r) - \beta Y_l^m(\theta,\phi)\sum_{k=-l}^{n-1}A_{n,k,l}^m r^{k-1}\exp(\sqrt{\frac{\beta}{D_{\text{cyt}}}}r),
\end{aligned}
$$

where we employed the relation $\Delta_{\theta,\phi}Y_l^m(\theta,\phi) = -\frac{l(l+1)}{r^2}Y_l^m(\theta,\phi)$. From equation (52), we further obtain

$$
\begin{aligned}
0 =& - l(l+1)D_{\text{cyt}}Y_l^m(\theta,\phi)\sum_{k=-l}^{n-1}A_{n,k,l}^m r^{k-3}\exp(\sqrt{\frac{\beta}{D_{\text{cyt}}}}r)\\
&+ Y_l^m(\theta,\phi)\sum_{k=-l}^{n-1}A_{n,k,l}^m\left(D_{\text{cyt}}(k-1)kr^{k-3} + 2\sqrt{D_{\text{cyt}}\beta}kr^{k-2} + \beta r^{k-1}\right)\exp(\sqrt{\frac{\beta}{D_{\text{cyt}}}}r)\\
&+ \alpha_n Y_l^m(\theta,\phi)\sum_{k=-l}^{n-2}A_{n-1,k,l}^m r^{k-1}\exp(\sqrt{\frac{\beta}{D_{\text{cyt}}}}r) - \beta Y_l^m(\theta,\phi)\sum_{k=-l}^{n-1}A_{n,k,l}^m r^{k-1}\exp(\sqrt{\frac{\beta}{D_{\text{cyt}}}}r).
\end{aligned}
$$

After canceling terms and rearranging, we have

$$
\begin{aligned}
0 =& \sum_{k=-l}^{n-1}A_{n,k,l}^m D_{\text{cyt}}\left[(k-1)k - l(l+1)\right]r^{k-3}\\
&\sum_{k=-l}^{n-1}A_{n,k,l}^m 2\sqrt{D_{\text{cyt}}\beta}kr^{k-2} + \alpha_n\sum_{k=-l+1}^{n-1}A_{n-1,k-1,l}^m r^{k-2}\\
=& \sum_{k=-l+1}^{n-1}A_{n,k,l}^m D_{\text{cyt}}\left[(k-1)k - l(l+1)\right]r^{k-3}\\
&\sum_{k=-l+1}^{n}A_{n,k-1,l}^m 2\sqrt{D_{\text{cyt}}\beta}(k-1)r^{k-3} + \alpha_n\sum_{k=-l+2}^{n}A_{n-1,k-2,l}^m r^{k-3}.
\end{aligned}
$$

Adjusting the sums for equating the coefficients results in

$$
\begin{aligned}
0 =& \sum_{k=-l+2}^{n-1}A_{n,k,l}^m D_{\text{cyt}}\left[(k-1)k - l(l+1)\right]r^{k-3}\\
&+ A_{n,-l,l}^m D_{\text{cyt}}\left[(-l)(-l+1) - l(l+1)\right]r^{-l-2}\\
&+ \sum_{k=-l+2}^{n-1}A_{n,k-1,l}^m 2\sqrt{D_{\text{cyt}}\beta}(k-1)r^{k-3}\\
&- A_{n,-l,l}^m 2l\sqrt{D_{\text{cyt}}\beta}r^{-l-2} + A_{n,n-1,l}^m 2D_{\text{cyt}}\sqrt{\frac{\beta}{D_{\text{cyt}}}}(n-1)r^{n-3}\\
&+ \sum_{k=-l+2}^{n-1}A_{n-1,k-2,l}^m\alpha_n r^{k-3} + \alpha_n A_{n-1,n-2,l}^m r^{n-3} = (*).
\end{aligned}
$$

After grouping the terms, we obtain

$$(*) = \sum_{k=-l+2}^{n-1} \left(A_{n,k,l}^m D_{\text{cyt}} \left[(k-1)k - l(l+1)\right] + A_{n,k-1,l}^m 2\sqrt{D_{\text{cyt}}\beta}(k-1) + \alpha_n A_{n-1,k-2,l}^m \right) r^{k-3}$$
$$- (A_{n,-l+1,l}^m + \sqrt{\frac{\beta}{D_{\text{cyt}}}} A_{n,-l,l}^m) D_{\text{cyt}} 2l r^{-l-2}$$
$$+ \left(\alpha_n A_{n-1,n-2,l}^m + 2(n-1) D_{\text{cyt}} A_{n,n-1,l}^m \sqrt{\frac{\beta}{D_{\text{cyt}}}} \right) r^{n-3}.$$

Therefore, we get the set of equations

$$D_{\text{cyt}} A_{n,k,l}^m \left[(k-1)k - l(l+1)\right] + A_{n,k-1,l}^m 2\sqrt{D_{\text{cyt}}\beta}(k-1) + \alpha_n A_{n-1,k-2,l}^m = 0, \qquad k = -l+2, \ldots, n-1$$
$$A_{n,-l+1,l}^m + \sqrt{\frac{\beta}{D_{\text{cyt}}}} A_{n,-l,l}^m = 0,$$
$$\alpha_n A_{n-1,n-2,l}^m + 2(n-1)\sqrt{D_{\text{cyt}}\beta} A_{n,n-1,l}^m = 0,$$

and similarly

$$D_{\text{cyt}} B_{n,k,l}^m \left[(k-1)k - l(l+1)\right] - B_{n,k-1,l}^m 2\sqrt{D_{\text{cyt}}\beta}(k-1) + \alpha_n B_{n-1,k-2,l}^m = 0, \qquad k = -l+2, \ldots, n-1$$
$$B_{n,-l+1,l}^m - \sqrt{\frac{\beta}{D_{\text{cyt}}}} B_{n,-l,l}^m = 0,$$
$$\alpha_n B_{n-1,n-2,l}^m + 2(n-1)\sqrt{D_{\text{cyt}}\beta} B_{n,n-1,l}^m = 0.$$

To compute the coefficients, we have to split these recursive relations into the cases $k \geq 2$ for $n \geq 2$, and $k \leq 1$. For $n \geq 2$ and $k \geq 2$, we have

$$A_{n,k-1,l}^m = -\frac{\alpha_n A_{n-1,k-2,l}^m + D_{\text{cyt}} A_{n,k,l}^m \left[(k-1)k - l(l+1)\right]}{2\sqrt{D_{\text{cyt}}\beta}(k-1)}, \qquad k = 2, \ldots, n-1, \tag{70}$$

with initial conditions

$$A_{n,n-1,l}^m = -\frac{\alpha_n A_{n-1,n-2,l}^m}{2(n-1)\sqrt{D_{\text{cyt}}\beta}}. \tag{71}$$

And similarly

$$B_{n,k-1,l}^m = \frac{\alpha_n B_{n-1,k-2,l}^m + D_{\text{cyt}} B_{n,k,l}^m \left[(k-1)k - l(l+1)\right]}{2\sqrt{D_{\text{cyt}}\beta}(k-1)}, \qquad k = 2, \ldots, n-1, \tag{72}$$

with initial conditions

$$B_{n,n-1,l}^m = -\frac{\alpha_n B_{n-1,n-2,l}^m}{2(n-1)\sqrt{D_{\text{cyt}}\beta}}. \tag{73}$$

For $k \leq 1$, we get

$$A_{n,k,l}^m = -\frac{A_{n,k-1,l}^m 2\sqrt{D_{\text{cyt}}\beta}(k-1) + \alpha_n A_{n-1,k-2,l}^m}{D_{\text{cyt}} \left[(k-1)k - l(l+1)\right]}, \qquad k = -l+2, \ldots, 1$$

with initial conditions

$$A_{n,-l+1,l}^m = \sqrt{\frac{\beta}{D_{\text{cyt}}}} A_{n,-l,l}^m.$$

And similarly, we have the recursive relations

$$B^m_{n,k,l} = \frac{B^m_{n,k-1,l} 2\sqrt{D_{\text{cyt}}\beta}(k-1) - \alpha_n B^m_{n-1,k-2,l}}{D_{\text{cyt}}\left[(k-1)k - l(l+1)\right]}, \qquad k = -l+2, \dots, 1$$

with initial conditions

$$B^m_{n,-l+1,l} = \sqrt{\frac{\beta}{D_{\text{cyt}}}} B^m_{n,-l,l}.$$

Using the above relations, all coefficients $A^m_{n,k,l}$ and $B^m_{n,k,l}$ can be expressed in terms of the coefficients of the upstream cascade elements $A^m_{n-1,k,l}$ and $B^m_{n-1,k,l}$, and the unkowns $A^m_{n,-l,l}$ and $B^m_{n,-l,l}$. For the determination of the coefficients $A^m_{n,-l,l}$ and $B^m_{n,-l,l}$, we use the boundary conditions (6) and (8). The diffusive flux of species P_n is given by

$$\begin{aligned}
D_{\text{cyt}} \frac{\partial \bar{P}^{\text{het}}_n(r,\theta,\phi)}{\partial r} &= \sum_{k=-l}^{n-1} D_{\text{cyt}} A^m_{n,k,l} Y^m_l(\theta,\phi) \frac{\partial}{\partial r} r^{k-1} \exp\left(\sqrt{\frac{\beta}{D_{\text{cyt}}}} r\right) \\
&\quad + \sum_{k=1}^{n-1} D_{\text{cyt}} B^m_{n,k,l} Y^m_l(\theta,\phi) \frac{\partial}{\partial r} r^{k-1} \exp\left(-\sqrt{\frac{\beta}{D_{\text{cyt}}}} r\right) \\
&= \sum_{k=-l}^{n-1} D_{\text{cyt}} A^m_{n,k,l} Y^m_l(\theta,\phi) \left[(k-1) r^{k-2} + \sqrt{\frac{\beta}{D_{\text{cyt}}}} r^{k-1}\right] \exp\left(\sqrt{\frac{\beta}{D_{\text{cyt}}}} r\right) \\
&\quad + \sum_{k=-l}^{n-1} D_{\text{cyt}} B^m_{n,k,l} Y^m_l(\theta,\phi) \left[(k-1) r^{k-2} - \sqrt{\frac{\beta}{D_{\text{cyt}}}} r^{k-1}\right] \exp\left(-\sqrt{\frac{\beta}{D_{\text{cyt}}}} r\right).
\end{aligned}$$

Assuming that cascade levels P_n with $n > 1$ have zero-boundary conditions on the cell membrane and nuclear membrane, we obtain from canceling the $Y^m_l(\theta,\phi)$ term

$$0 = \sum_{k=-l}^{n-1} A^m_{n,k,l} \left[(k-1) R^{k-2} + \sqrt{\frac{\beta}{D_{\text{cyt}}}} R^{k-1}\right] \exp\left(\sqrt{\frac{\beta}{D_{\text{cyt}}}} R\right) \tag{74}$$

$$+ \sum_{k=-l}^{n-1} B^m_{n,k,l} \left[(k-1) R^{k-2} - \sqrt{\frac{\beta}{D_{\text{cyt}}}} R^{k-1}\right] \exp\left(-\sqrt{\frac{\beta}{D_{\text{cyt}}}} R\right), \tag{75}$$

for $R = R_{\text{cell}}$ or $R = R_{\text{nuc}}$. This relation results in a linear equation system, that can be solved for $A^m_{n,-l,l}$ and $B^m_{n,-l,l}$.

4.4 Special case: Linear gradient for the pure cytosolic (PC) cascade

4.4.1 Without nucleus

In this case, we assume that the signal is of the form

$$\bar{P}_0(\theta,\phi) = A^0_{0,0,0} + A^m_{n,0,1} Y^m_1(\theta,\phi). \tag{76}$$

For the spatially homogeneous part we know the solution. Furthermore, we already the solution of the heterogeneous part of the first cascade level from section 3.2, which reads

$$\begin{aligned}
\bar{P}_1 &= A_1 i_1(\sqrt{\frac{\beta}{D_{\text{cyt}}}}r) = A_1 \frac{(\sqrt{\frac{\beta}{D_{\text{cyt}}}}r)\cosh(\sqrt{\frac{\beta}{D_{\text{cyt}}}}r) - \sinh(\sqrt{\frac{\beta}{D_{\text{cyt}}}}r)}{(\sqrt{\frac{\beta}{D_{\text{cyt}}}}r)^2} \\
&= -A_1 \frac{D_{\text{cyt}}}{\beta} r^{-2} \exp(\sqrt{\frac{\beta}{D_{\text{cyt}}}}r) + A_1 \frac{D_{\text{cyt}}}{\beta} r^{-2} \exp(-\sqrt{\frac{\beta}{D_{\text{cyt}}}}r) \\
&+ A_1 \sqrt{\frac{D_{\text{cyt}}}{\beta}} r^{-1} \exp(\sqrt{\frac{\beta}{D_{\text{cyt}}}}r) + A_1 \sqrt{\frac{D_{\text{cyt}}}{\beta}} r^{-1} \exp(-\sqrt{\frac{\beta}{D_{\text{cyt}}}}r) \\
&= A_{1,-1,1} r^{-2} \exp(\sqrt{\frac{\beta}{D_{\text{cyt}}}}r) + B_{1,-1,1} r^{-2} \exp(-\sqrt{\frac{\beta}{D_{\text{cyt}}}}r) \\
&+ A_{1,0,1} r^{-1} \exp(\sqrt{\frac{\beta}{D_{\text{cyt}}}}r) + B_{1,0,1} r^{-1} \exp(-\sqrt{\frac{\beta}{D_{\text{cyt}}}}r),
\end{aligned} \tag{77}$$

where $A_1 = -\frac{\beta}{D_{\text{cyt}}} A_{1,-1,1} = \frac{\beta}{D_{\text{cyt}}} B_{1,-1,1} = \sqrt{\frac{\beta}{D_{\text{cyt}}}} A_{1,0,1} = \sqrt{\frac{\beta}{D_{\text{cyt}}}} B_{1,0,1}$ fulfills the ansatz for the first cascade level. For the second cascade level we use the ansatz

$$\bar{P}_2(r,\theta,\phi) = Y_1(\theta,\phi) \left(A_2 i_1(\sqrt{\frac{\beta}{D_{\text{cyt}}}}r) + B_2 \sinh(\sqrt{\frac{\beta}{D_{\text{cyt}}}}) \right). \tag{78}$$

The Laplace operator of the second term gives

$$\begin{aligned}
&\Delta B_2 Y_1(\theta,\phi) \sinh(\sqrt{\frac{\beta}{D_{\text{cyt}}}}) \\
&= B_2 Y_1(\theta,\phi) \Delta_r \sinh(\sqrt{\frac{\beta}{D_{\text{cyt}}}}) + B_2 \Delta_{\theta,\phi} Y_1(\theta,\phi) \sinh(\sqrt{\frac{\beta}{D_{\text{cyt}}}}) \\
&= B_2 Y_1(\theta,\phi) \left(\frac{\partial^2}{\partial^2 r} + \frac{2}{r}\frac{\partial}{\partial r} \right) \sinh(\sqrt{\frac{\beta}{D_{\text{cyt}}}}r) - 2B_2 Y_1(\theta,\phi) \frac{\sinh(\sqrt{\frac{\beta}{D_{\text{cyt}}}}r)}{r^2} \\
&= B_2 Y_1(\theta,\phi) \left(\frac{\beta}{D_{\text{cyt}}} \sinh(r) + \frac{2}{r}\sqrt{\frac{\beta}{D_{\text{cyt}}}} \cosh(r) \right) - 2B_2 Y_1(\theta,\phi) \frac{\sinh(\sqrt{\frac{\beta}{D_{\text{cyt}}}}r)}{r^2} \\
&= B_2 Y_1(\theta,\phi) \frac{\beta}{D_{\text{cyt}}} \sinh(\sqrt{\frac{\beta}{D_{\text{cyt}}}}r) + 2\frac{\beta}{D_{\text{cyt}}} B_2 Y_1(\theta,\phi) i_1(\sqrt{\frac{\beta}{D_{\text{cyt}}}}r).
\end{aligned} \tag{79}$$

Therefore, we have

$$\begin{aligned}
0 &= D_{\text{cyt}} \Delta \bar{P}_2 + \alpha_2 \bar{P}_1 - \beta \bar{P}_2 \\
&= \alpha_2 A_1 Y_1(\theta,\phi) i_1(\sqrt{\frac{\beta}{D_{\text{cyt}}}}r) + 2B_2 \beta Y_1(\theta,\phi) i_1(\sqrt{\frac{\beta}{D_{\text{cyt}}}}r),
\end{aligned} \tag{80}$$

which gives the relation $B_2 = -\frac{\alpha_2 A_1}{2\beta}$. This can also be rephrased in the relationships

$$A_2 = -\frac{\beta}{D_{\text{cyt}}} A_{2,-1,1} = \frac{\beta}{D_{\text{cyt}}} B_{2,-1,1} = \sqrt{\frac{\beta}{D_{\text{cyt}}}} A_{2,0,1} = \sqrt{\frac{\beta}{D_{\text{cyt}}}} B_{2,0,1} \tag{81}$$

$$B_2 = -\frac{\alpha_2 A_1}{2\beta} = A_{2,1,1} = B_{2,1,1}. \tag{82}$$

For the determination of A_2, we use the boundary condition

$$
\begin{aligned}
0 &= D_{\text{cyt}} \left.\frac{\partial}{\partial r}\bar{P}_2(r)\right|_{r=R_{\text{cell}}} \\
&= D_{\text{cyt}}A_2\sqrt{\frac{\beta}{D_{\text{cyt}}}}i_2(\sqrt{\frac{\beta}{D_{\text{cyt}}}}R_{\text{cell}}) + D_{\text{cyt}}\frac{A_2}{R_{\text{cell}}}i_1(\sqrt{\frac{\beta}{D_{\text{cyt}}}}R_{\text{cell}}) + D_{\text{cyt}}B_2\sqrt{\frac{\beta}{D_{\text{cyt}}}}\cosh(\sqrt{\frac{\beta}{D_{\text{cyt}}}}R_{\text{cell}}).
\end{aligned} \tag{83}
$$

This yields the relation

$$
A_2 = -\frac{B_2\cosh(\sqrt{\frac{\beta}{D_{\text{cyt}}}}R_{\text{cell}})}{i_2(\sqrt{\frac{\beta}{D_{\text{cyt}}}}R_{\text{cell}}) + \sqrt{\frac{D_{\text{cyt}}}{\beta}}\frac{1}{R_{\text{cell}}}i_1(\sqrt{\frac{\beta}{D_{\text{cyt}}}}R_{\text{cell}})}. \tag{84}
$$

For the third cascade element, we use the ansatz

$$
\bar{P}_3(r,\theta,\phi) = Y_1(\theta,\phi)\left(A_3 i_1(\sqrt{\frac{\beta}{D_{\text{cyt}}}}r) + B_3\sinh(\sqrt{\frac{\beta}{D_{\text{cyt}}}}) + C_3 r\cosh(\sqrt{\frac{\beta}{D_{\text{cyt}}}}r)\right) \tag{85}
$$

The Laplace operator of the third term gives

$$
\begin{aligned}
&\Delta C_3 Y_1(\theta,\phi) r\cosh(\sqrt{\frac{\beta}{D_{\text{cyt}}}}r) \\
&= C_3Y_1(\theta,\phi)\left(\frac{\partial^2}{\partial^2 r} + \frac{2}{r}\frac{\partial}{\partial r}\right) r\cosh(\sqrt{\frac{\beta}{D_{\text{cyt}}}}r) - 2C_3Y_1(\theta,\phi)\frac{\cosh(\sqrt{\frac{\beta}{D_{\text{cyt}}}}r)}{r} \\
&= C_3Y_1(\theta,\phi)\left(2\sqrt{\frac{\beta}{D_{\text{cyt}}}}\sinh(\sqrt{\frac{\beta}{D_{\text{cyt}}}}r) + r\frac{\beta}{D_{\text{cyt}}}\cosh(\sqrt{\frac{\beta}{D_{\text{cyt}}}}r) + \frac{2}{r}\cosh(\sqrt{\frac{\beta}{D_{\text{cyt}}}}r)\right. \\
&\left. +2\sqrt{\frac{\beta}{D_{\text{cyt}}}}\sinh(\sqrt{\frac{\beta}{D_{\text{cyt}}}}r)\right) - 2C_3Y_1(\theta,\phi)\frac{\cosh(\sqrt{\frac{\beta}{D_{\text{cyt}}}}r)}{r} \\
&= 4C_3\sqrt{\frac{\beta}{D_{\text{cyt}}}}Y_1(\theta,\phi)\sinh(\sqrt{\frac{\beta}{D_{\text{cyt}}}}r) + \frac{\beta}{D_{\text{cyt}}}C_3Y_1(\theta,\phi)r\cosh(\sqrt{\frac{\beta}{D_{\text{cyt}}}}r).
\end{aligned} \tag{86}
$$

Therefore, we have

$$
\begin{aligned}
0 &= D_{\text{cyt}}\Delta\bar{P}_3 + \alpha_3\bar{P}_2 - \beta\bar{P}_3 \\
&= \alpha_3A_2Y_1(\theta,\phi)i_1(\sqrt{\frac{\beta}{D_{\text{cyt}}}}r) + \alpha_3B_2Y_1(\theta,\phi)\sinh(\sqrt{\frac{\beta}{D_{\text{cyt}}}}r) \\
&+ 2B_3\beta Y_1(\theta,\phi)i_1(\sqrt{\frac{\beta}{D_{\text{cyt}}}}r) + 4C_3\sqrt{D_{\text{cyt}}\beta}Y_1(\theta,\phi)\sinh(\sqrt{\frac{\beta}{D_{\text{cyt}}}}r),
\end{aligned} \tag{87}
$$

which gives the relation $B_3 = -\frac{\alpha_3 A_2}{2\beta}$ and $C_3 = -\frac{\alpha_3 B_2}{4\sqrt{D_{\text{cyt}}\beta}}$. This can also be rephrased in the relationships

$$
A_3 = -\frac{\beta}{D_{\text{cyt}}}A_{3,-1,1} = \frac{\beta}{D_{\text{cyt}}}B_{3,-1,1} = \sqrt{\frac{\beta}{D_{\text{cyt}}}}A_{3,0,1} = \sqrt{\frac{\beta}{D_{\text{cyt}}}}B_{3,0,1}, \tag{88}
$$

$$
B_3 = -\frac{\alpha_3 A_2}{2\beta} = A_{3,1,1} = B_{3,1,1}, \tag{89}
$$

$$
C_3 = -\frac{\alpha_3 B_2}{4\sqrt{D_{\text{cyt}}\beta}} = A_{3,2,1} = B_{3,2,1}. \tag{90}
$$

For the determination of A_3, we use the boundary condition

$$0 = D_{\text{cyt}} \left.\frac{\partial}{\partial r}\bar{P}_3(r)\right|_{r=R_{\text{cell}}}$$
$$= D_{\text{cyt}} A_3 \sqrt{\frac{\beta}{D_{\text{cyt}}}} i_2(\sqrt{\frac{\beta}{D_{\text{cyt}}}} R_{\text{cell}}) + D_{\text{cyt}} \frac{A_3}{R_{\text{cell}}} i_1(\sqrt{\frac{\beta}{D_{\text{cyt}}}} R_{\text{cell}}) + D_{\text{cyt}} B_3 \sqrt{\frac{\beta}{D_{\text{cyt}}}} \cosh(\sqrt{\frac{\beta}{D_{\text{cyt}}}} R_{\text{cell}})$$
$$+ D_{\text{cyt}} C_3 \cosh(\sqrt{\frac{\beta}{D_{\text{cyt}}}} R_{\text{cell}}) + D_{\text{cyt}} C_3 \sqrt{\frac{\beta}{D_{\text{cyt}}}} R_{\text{cell}} \sinh(\sqrt{\frac{\beta}{D_{\text{cyt}}}} R_{\text{cell}}). \tag{91}$$

Therefore, A_3 is given by

$$A_3 = -\frac{B_3 \sqrt{\frac{\beta}{D_{\text{cyt}}}} \cosh(\sqrt{\frac{\beta}{D_{\text{cyt}}}} R_{\text{cell}}) + C_3 \cosh(\sqrt{\frac{\beta}{D_{\text{cyt}}}} R_{\text{cell}}) + C_3 \sqrt{\frac{\beta}{D_{\text{cyt}}}} R_{\text{cell}} \sinh(\sqrt{\frac{\beta}{D_{\text{cyt}}}} R_{\text{cell}})}{\sqrt{\frac{\beta}{D_{\text{cyt}}}} i_2(\sqrt{\frac{\beta}{D_{\text{cyt}}}} R_{\text{cell}}) + \frac{1}{R_{\text{cell}}} i_1(\sqrt{\frac{\beta}{D_{\text{cyt}}}} R_{\text{cell}})}.$$

4.4.2 With nucleus

For the first cascade level we use the ansatz

$$\bar{P}_1^{\text{het}}(\theta,\phi,r) = Y_l^m(\theta,\phi) \sum_{k=-1}^{0} A^m_{n,k,l} r^{k-1} \exp(\sqrt{\frac{\beta}{D_{\text{cyt}}}} r) + Y_l^m(\theta,\phi) \sum_{k=-1}^{0} B^m_{n,k,l} r^{k-1} \exp(-\sqrt{\frac{\beta}{D_{\text{cyt}}}} r). \tag{92}$$

We already know that

$$\bar{P}_1^{\text{het}}(\theta,\phi,r) = A^m_{1,1} i_1(r\sqrt{\frac{\beta}{D_{\text{cyt}}}}) Y_l^m(\theta,\phi) + B^m_{1,1} k_1(r\sqrt{\frac{\beta}{D_{\text{cyt}}}})) Y_l^m(\theta,\phi)$$
$$= A^m_{1,1} \frac{\left(\sqrt{\frac{\beta}{D_{\text{cyt}}}} r\right)\left(\exp(r\sqrt{\frac{\beta}{D_{\text{cyt}}}}) + \exp(-r\sqrt{\frac{\beta}{D_{\text{cyt}}}})\right) - \exp(r\sqrt{\frac{\beta}{D_{\text{cyt}}}}) + \exp(-r\sqrt{\frac{\beta}{D_{\text{cyt}}}})}{2\left(\sqrt{\frac{\beta}{D_{\text{cyt}}}} r\right)^2}$$
$$+ B^m_{1,1} \frac{\exp(-r\sqrt{\frac{\beta}{D_{\text{cyt}}}})\left(1 + \sqrt{\frac{\beta}{D_{\text{cyt}}}} r\right)}{\left(\sqrt{\frac{\beta}{D_{\text{cyt}}}} r\right)^2}$$
$$= -A^m_{1,l} \frac{D_{\text{cyt}}}{2\beta} r^{-2} \exp(r\sqrt{\frac{\beta}{D_{\text{cyt}}}}) + A^m_{1,l} \sqrt{\frac{D_{\text{cyt}}}{4\beta}} r^{-1} \exp(r\sqrt{\frac{\beta}{D_{\text{cyt}}}})$$
$$+ (B^m_{1,l} + \frac{1}{2} A^m_{1,l}) \frac{D_{\text{cyt}}}{\beta} r^{-2} \exp(-r\sqrt{\frac{\beta}{D_{\text{cyt}}}}) + (B^m_{1,l} + \frac{1}{2} A^m_{1,l}) \sqrt{\frac{D_{\text{cyt}}}{\beta}} r^{-1} \exp(-r\sqrt{\frac{\beta}{D_{\text{cyt}}}})$$

Therefore, we have $A^m_{1,-1,1} = -A^m_{1,1} \frac{D_{\text{cyt}}}{2\beta}$, $A^m_{1,0,1} = A^m_{1,1} \sqrt{\frac{D_{\text{cyt}}}{4\beta}}$, $B^m_{1,-1,1} = (B^m_{1,1} + \frac{1}{2} A^m_{1,l}) \frac{D_{\text{cyt}}}{\beta}$ and $B^m_{1,0,1} = (B^m_{1,1} + \frac{1}{2} A^m_{1,l}) \sqrt{\frac{D_{\text{cyt}}}{\beta}}$. For the next cascade level, we can use the following relations:

$$-2D_{\text{cyt}} A^m_{2,1,1} + \alpha_2 A^m_{1,-1,1} = 0, \tag{93}$$

$$A^m_{2,0,1} + \sqrt{\frac{\beta}{D_{\text{cyt}}}} A^m_{2,-1,1} = 0, \tag{94}$$

$$\alpha_2 A^m_{1,0,1} + 2D_{\text{cyt}} A^m_{2,1,1} \sqrt{\frac{\beta}{D_{\text{cyt}}}} = 0, \tag{95}$$

and

$$-2D_{\mathrm{cyt}}B^m_{2,1,1} + \alpha_n B^m_{1,-1,1} = 0, \tag{96}$$

$$B^m_{2,0,1} - \sqrt{\frac{\beta}{D_{\mathrm{cyt}}}}B^m_{2,-1,1} = 0, \tag{97}$$

$$\alpha_2 B^m_{1,0,1} - 2D_{\mathrm{cyt}}B^m_{2,1,1}\sqrt{\frac{\beta}{D_{\mathrm{cyt}}}} = 0. \tag{98}$$

Therefore, we get $A^m_{2,1,1} = \frac{\alpha_2 A^m_{1,-1,1}}{2D_{\mathrm{cyt}}}$ and $B^m_{2,1,1} = \frac{\alpha_2 B^m_{1,-1,1}}{2D_{\mathrm{cyt}}}$. For the determination of $A^m_{2,-1,1}$, $A^m_{2,0,1}$, $B^m_{2,-1,1}$ and $B^m_{2,0,1}$, we use the boundary conditions

$$\begin{aligned}
0 &= \sum_{k=-l}^{n-1} A^m_{n,k,l}\left[(k-1)R^{k-2} + \sqrt{\frac{\beta}{D_{\mathrm{cyt}}}}R^{k-1}\right]\exp(\sqrt{\frac{\beta}{D_{\mathrm{cyt}}}}R)\\
&+ \sum_{k=-l}^{n-1} B^m_{n,k,l}\left[(k-1)R^{k-2} - \sqrt{\frac{\beta}{D_{\mathrm{cyt}}}}R^{k-1}\right]\exp(-\sqrt{\frac{\beta}{D_{\mathrm{cyt}}}}R)\\
&= A^m_{2,-1,1}\left[-2R^{-3} + \sqrt{\frac{\beta}{D_{\mathrm{cyt}}}}R^{-2}\right]\exp(\sqrt{\frac{\beta}{D_{\mathrm{cyt}}}}R)\\
&+ A^m_{2,0,1}\left[-R^{-2} + \sqrt{\frac{\beta}{D_{\mathrm{cyt}}}}R^{-1}\right]\exp(\sqrt{\frac{\beta}{D_{\mathrm{cyt}}}}R) + A^m_{2,1,1}\sqrt{\frac{\beta}{D_{\mathrm{cyt}}}}\exp(\sqrt{\frac{\beta}{D_{\mathrm{cyt}}}}R)\\
&+ B^m_{2,-1,1}\left[-2R^{-3} - \sqrt{\frac{\beta}{D_{\mathrm{cyt}}}}R^{-2}\right]\exp(-\sqrt{\frac{\beta}{D_{\mathrm{cyt}}}}R)\\
&+ B^m_{2,0,1}\left[-R^{-2} - \sqrt{\frac{\beta}{D_{\mathrm{cyt}}}}R^{-1}\right]\exp(-\sqrt{\frac{\beta}{D_{\mathrm{cyt}}}}R) - B^m_{2,1,1}\sqrt{\frac{\beta}{D_{\mathrm{cyt}}}}\exp(-\sqrt{\frac{\beta}{D_{\mathrm{cyt}}}}R),
\end{aligned}$$

at $R = R_{\mathrm{cell}}$ and $R = R_{\mathrm{nuc}}$. Using the relationships $A^m_{2,0,1} = -\sqrt{\frac{\beta}{D_{\mathrm{cyt}}}}A^m_{2,-1,1}$ and $B^m_{2,0,1} = \sqrt{\frac{\beta}{D_{\mathrm{cyt}}}}B^m_{2,-1,1}$ and further rearrangement yields

$$\begin{aligned}
&A^m_{2,-1,1}\left[-2R^{-3} + 2\sqrt{\frac{\beta}{D_{\mathrm{cyt}}}}R^{-2} - \frac{\beta}{D_{\mathrm{cyt}}}R^{-1}\right]\exp(\sqrt{\frac{\beta}{D_{\mathrm{cyt}}}}R)\\
&+ B^m_{2,-1,1}\left[-2R^{-3} - 2\sqrt{\frac{\beta}{D_{\mathrm{cyt}}}}R^{-2} - \frac{\beta}{D_{\mathrm{cyt}}}R^{-1}\right]\exp(-\sqrt{\frac{\beta}{D_{\mathrm{cyt}}}}R)\\
&= B^m_{2,1,1}\sqrt{\frac{\beta}{D_{\mathrm{cyt}}}}\exp(-\sqrt{\frac{\beta}{D_{\mathrm{cyt}}}}R) - A^m_{2,1,1}\sqrt{\frac{\beta}{D_{\mathrm{cyt}}}}\exp(\sqrt{\frac{\beta}{D_{\mathrm{cyt}}}}R)
\end{aligned}$$

This linear equation system can then be solved for $A^m_{2,-1,1}$ and $B^m_{2,-1,1}$. For the third cascade level ($n = 3$) we obtan

$$\begin{aligned}
-2D_{\mathrm{cyt}}A^m_{3,1,1} + \alpha_3 A^m_{2,-1,1} &= 0,\\
A^m_{3,1,1}2\sqrt{D_{\mathrm{cyt}}\beta} + \alpha_3 A^m_{2,0,1} &= 0,\\
A^m_{3,0,1} + \sqrt{\frac{\beta}{D_{\mathrm{cyt}}}}A^m_{3,-1,1} &= 0,\\
\alpha_3 A^m_{2,1,1} + 4D_{\mathrm{cyt}}A^m_{3,2,1}\sqrt{\frac{\beta}{D_{\mathrm{cyt}}}} &= 0,
\end{aligned}$$

and

$$-2D_{\text{cyt}}B^m_{3,1,1} + \alpha_3 B^m_{2,-1,1} = 0,$$
$$-B^m_{3,1,1}2\sqrt{D_{\text{cyt}}\beta} + \alpha_3 B^m_{2,0,1} = 0,$$
$$B^m_{3,0,1} - \sqrt{\frac{\beta}{D_{\text{cyt}}}}B^m_{3,-1,1} = 0,$$
$$\alpha_3 B^m_{2,1,1} - 4D_{\text{cyt}}B^m_{3,2,1}\sqrt{\frac{\beta}{D_{\text{cyt}}}} = 0.$$

Therefore, we get $A^m_{3,1,1} = \frac{\alpha_3 A^m_{2,-1,1}}{2D_{\text{cyt}}}$, $B^m_{3,1,1} = \frac{\alpha_3 B^m_{2,-1,1}}{2D_{\text{cyt}}}$, $A^m_{3,2,1} = -\frac{\alpha_3 A^m_{2,1,1}}{4\sqrt{D_{\text{cyt}}\beta}}$ and $B^m_{3,2,1} = -\frac{\alpha_3 B^m_{2,1,1}}{4\sqrt{D_{\text{cyt}}\beta}}$. For the determination of $A^m_{3,-1,1}$ and $B^m_{3,-1,1}$, we use the boundary conditions

$$\begin{aligned}
0 &= \sum_{k=-l}^{n-1} A^m_{n,k,l}\left[(k-1)R^{k-2} + \sqrt{\frac{\beta}{D_{\text{cyt}}}}R^{k-1}\right]\exp(\sqrt{\frac{\beta}{D_{\text{cyt}}}}R) \\
&+ \sum_{k=-l}^{n-1} B^m_{n,k,l}\left[(k-1)R^{k-2} - \sqrt{\frac{\beta}{D_{\text{cyt}}}}R^{k-1}\right]\exp(-\sqrt{\frac{\beta}{D_{\text{cyt}}}}R) \\
&= A^m_{3,-1,1}\left[-2R^{-3} + \sqrt{\frac{\beta}{D_{\text{cyt}}}}R^{-2}\right]\exp(\sqrt{\frac{\beta}{D_{\text{cyt}}}}R) \\
&+ A^m_{3,0,1}\left[-R^{-2} + \sqrt{\frac{\beta}{D_{\text{cyt}}}}R^{-1}\right]\exp(\sqrt{\frac{\beta}{D_{\text{cyt}}}}R) + A^m_{3,1,1}\sqrt{\frac{\beta}{D_{\text{cyt}}}}\exp(\sqrt{\frac{\beta}{D_{\text{cyt}}}}R) \\
&+ A^m_{3,2,1}\left[1 + \sqrt{\frac{\beta}{D_{\text{cyt}}}}R\right]\exp(\sqrt{\frac{\beta}{D_{\text{cyt}}}}R) \\
&+ B^m_{3,-1,1}\left[-2R^{-3} - \sqrt{\frac{\beta}{D_{\text{cyt}}}}R^{-2}\right]\exp(-\sqrt{\frac{\beta}{D_{\text{cyt}}}}R) \\
&+ B^m_{3,0,1}\left[-R^{-2} - \sqrt{\frac{\beta}{D_{\text{cyt}}}}R^{-1}\right]\exp(-\sqrt{\frac{\beta}{D_{\text{cyt}}}}R) - B^m_{3,1,1}\sqrt{\frac{\beta}{D_{\text{cyt}}}}\exp(-\sqrt{\frac{\beta}{D_{\text{cyt}}}}R) \\
&+ B^m_{3,2,1}\left[1 - \sqrt{\frac{\beta}{D_{\text{cyt}}}}R\right]\exp(-\sqrt{\frac{\beta}{D_{\text{cyt}}}}R)
\end{aligned}$$

for $R = R_{\text{cell}}$ and $R = R_{\text{nuc}}$. Using the relationships $A^m_{3,0,1} = -\sqrt{\frac{\beta}{D_{\text{cyt}}}}A^m_{3,-1,1}$ and $B^m_{3,0,1} = \sqrt{\frac{\beta}{D_{\text{cyt}}}}B^m_{3,-1,1}$ and further rearrangement yields

$$\begin{aligned}
&A^m_{3,-1,1}\left[-2R^{-3} + 2\sqrt{\frac{\beta}{D_{\text{cyt}}}}R^{-2} - \frac{\beta}{D_{\text{cyt}}}R^{-1}\right]\exp(\sqrt{\frac{\beta}{D_{\text{cyt}}}}R) \\
&+ B^m_{3,-1,1}\left[-2R^{-3} - 2\sqrt{\frac{\beta}{D_{\text{cyt}}}}R^{-2} - \frac{\beta}{D_{\text{cyt}}}R^{-1}\right]\exp(-\sqrt{\frac{\beta}{D_{\text{cyt}}}}R) \\
&= -A^m_{3,1,1}\sqrt{\frac{\beta}{D_{\text{cyt}}}}\exp(\sqrt{\frac{\beta}{D_{\text{cyt}}}}R) + B^m_{3,1,1}\sqrt{\frac{\beta}{D_{\text{cyt}}}}\exp(-\sqrt{\frac{\beta}{D_{\text{cyt}}}}R) \\
&- A^m_{3,2,1}\left[1 + \sqrt{\frac{\beta}{D_{\text{cyt}}}}R\right]\exp(\sqrt{\frac{\beta}{D_{\text{cyt}}}}R) - B^m_{3,2,1}\left[1 - \sqrt{\frac{\beta}{D_{\text{cyt}}}}R\right]\exp(-\sqrt{\frac{\beta}{D_{\text{cyt}}}}R),
\end{aligned} \tag{99}$$

for $R = R_{\text{cell}}$ and $R = R_{\text{nuc}}$. This is again a linear equation system that can be solved explicitely for $A^m_{3,-1,1}$ and $B^m_{3,-1,1}$.

5 Comparison of the spatial model with the assumption of a well-mixed compartment

Integration of the equations (1) for the membrane-bound components over the surface M_{cell} gives

$$\frac{d}{dt}\int\limits_{M_{\text{cell}}} P_n dA = D_{\text{mem}} \int\limits_{M_{\text{cell}}} \Delta_\Gamma P_n dA + \alpha_n \int\limits_{M_{\text{cell}}} P_{n-1} dA - \beta_n \int\limits_{M_{\text{cell}}} P_n dA \qquad n = 1, \ldots, M, \tag{100}$$

where P_0 is the input signal. Since there are no boundaries on the membrane, Green's theorem gives

$$\frac{d}{dt}\int\limits_{M_{\text{cell}}} P_n dA = \alpha_n \int\limits_{M_{\text{cell}}} P_{n-1} dA - \beta_n \int\limits_{M_{\text{cell}}} P_n dA \qquad n = 1, \ldots, M, \tag{101}$$

which is an ordinary differential equation for the total number of molecules per cell. Dividing by the cell-membrane area $|M_{\text{cell}}|$, we obtain

$$\frac{d}{dt} P_n^{\text{avg}} = \alpha_n P_{n-1}^{\text{avg}} - \beta_n P_n^{\text{avg}}. \tag{102}$$

Equation (2), which is describing the membrane-cytosolic interface, is integrated over the cell volume V_{cell}, which gives

$$\frac{d}{dt}\int\limits_{V_{\text{cell}}} P_{M+1} dV = D_{\text{cyt}} \int\limits_{V_{\text{cell}}} \Delta P_{M+1} dV - \beta_{M+1} \int\limits_{V_{\text{cell}}} P_{M+1} dV. \tag{103}$$

Applying Green's theorem gives

$$\frac{d}{dt}\int\limits_{V_{\text{cell}}} P_{M+1} dV = \int\limits_{M_{\text{cell}}} \alpha_{M+1} P_M - \gamma P_{M+1} dA - \beta_{M+1} \int\limits_{V_{\text{cell}}} P_{M+1} dV. \tag{104}$$

This relation is a mass conservation equation. Similarly, we obtain for the cytosolic components P_n ($M+1 < n \leq N$) after integration over the cell volume V_{cell} and applying Green's theorem :

$$\frac{d}{dt}\int\limits_{V_{\text{cell}}} P_n dV = \alpha_n \int\limits_{V_{\text{cell}}} P_{n-1} dV - \beta_n \int\limits_{V_{\text{cell}}} P_n dV. \tag{105}$$

5.1 ODE system for the average concentration in the case $\gamma = 0$

In the case $\gamma = 0$, the dynamics of the average concentration of signaling components can be described exactly by an ODE system. The mass conservation equation (104) becomes

$$\frac{d}{dt}\int\limits_{V_{\text{cell}}} P_{M+1} dV = \alpha_{M+1} \int\limits_{M_{\text{cell}}} P_M dA - \beta_{M+1} \int\limits_{V_{\text{cell}}} P_{M+1} dV. \tag{106}$$

The equation can be rewritten in for the average number per volume for P_{M+1} or membrane area for P_M:

$$\frac{d}{dt} P_{M+1}^{\text{avg}} = \alpha_{M+1} \frac{|M_{\text{cell}}|}{|V_{\text{cell}}|} P_M^{\text{avg}} - \beta_{M+1} P_{M+1}^{\text{avg}}. \tag{107}$$

Similarly, we obtain for the average cocnentration of cytosolic components P_n ($M+1 < n \leq N$)

$$\frac{d}{dt} P_n^{\text{avg}} = \alpha_n P_{n-1}^{\text{avg}} - \beta_n P_n^{\text{avg}}. \tag{108}$$

In summary, this results in an equation system of ordinary differential equations for the average concentration

$$\frac{d}{dt}P_1^{\text{avg}} = \alpha_1 P_0^{\text{avg}} - \beta_1 P_1^{\text{avg}} \qquad \text{on the membrane,} \tag{109}$$

$$\vdots$$

$$\frac{d}{dt}P_M^{\text{avg}} = \alpha_M P_{M-1}^{\text{avg}} - \beta_M P_M^{\text{avg}} \qquad \text{on the membrane,} \tag{110}$$

$$\frac{d}{dt}P_{M+1}^{\text{avg}} = \alpha_{M+1}\frac{|M_{\text{cell}}|}{|V_{\text{cell}}|}P_M^{\text{avg}} - \beta_{M+1}P_{M+1}^{\text{avg}} \qquad \text{at the membrane-cytosolic interface,} \tag{111}$$

$$\frac{d}{dt}P_{M+2}^{\text{avg}} = \alpha_{M+2} P_{M+1}^{\text{avg}} - \beta_{M+2} P_{M+2}^{\text{avg}} \qquad \text{in the cytosol,} \tag{112}$$

$$\vdots$$

$$\frac{d}{dt}P_N^{\text{avg}} = \alpha_N P_{N-1}^{\text{avg}} - \beta_N P_N^{\text{avg}} \qquad \text{in the cytosol.} \tag{113}$$

6 Estimates for the maximum, minimum and average concentration levels

6.1 Lower bound for the maximal concentration

Using the mass conservation equation (104) we get the inequality for the maximal concentration $P_{M+1}^{\max}$

$$\frac{d}{dt}\int\limits_{V_{\text{cell}}} P_{M+1}dV \geq \int\limits_{M_{\text{cell}}} \alpha_{M+1}P_M - \gamma P_{M+1}^{\max}dA - \beta_{M+1}\int\limits_{V_{\text{cell}}} P_{M+1}^{\max}dV. \tag{114}$$

Dividing by the cytosolic volume gives

$$\frac{1}{|V_{\text{cell}}|}\frac{d}{dt}\int\limits_{V_{\text{cell}}} P_{M+1}dV \geq \alpha_{M+1}\frac{|M_{\text{cell}}|}{|V_{\text{cell}}|}P_M^{\text{avg}} - \gamma\frac{|M_{\text{cell}}|}{|V_{\text{cell}}|}P_{M+1}^{\max} - \beta_{M+1}P_{M+1}^{\max}. \tag{115}$$

In the steady state we thus obtain

$$\left(\gamma\frac{|M_{\text{cell}}|}{|V_{\text{cell}}|} + \beta_{M+1}\right)\bar{P}_{M+1}^{\max} \geq \alpha_{M+1}\frac{|M_{\text{cell}}|}{|V_{\text{cell}}|}\bar{P}_M^{\text{avg}}. \tag{116}$$

Rearranging gives the estimate

$$\bar{P}_{M+1}^{\max} \geq \frac{\alpha_{M+1}|M_{\text{cell}}|\bar{P}_M^{\text{avg}}}{\gamma|M_{\text{cell}}| + \beta_{M+1}|V_{\text{cell}}|}. \tag{117}$$

Due to the maximum principle [6] for elliptic partial differential equations, the maximum of P_{M+1} is obtained on the boundary, which is the membrane of the cell. Note that all estimates are only true for positive functions P_n, which is a natural assumption for this biophysical system.

6.2 Upper bound for the minimal concentration

From the equation of mass conservation (104) we get the estimate for the minimal concentration $P_{M+1}^{\min}$

$$\frac{d}{dt}\int\limits_{V_{\text{cell}}} P_{M+1}dV \leq \int\limits_{M_{\text{cell}}} \alpha_{M+1}P_M - \gamma P_{M+1}^{\min}dA - \beta_{M+1}\int\limits_{V_{\text{cell}}} P_{M+1}^{\min}dV \tag{118}$$

Dividing by the cytosolic volume gives

$$\frac{1}{|V_{\text{cell}}|}\frac{d}{dt}\int\limits_{V_{\text{cell}}} P_{M+1}dV \leq \alpha_{M+1}\frac{|M_{\text{cell}}|}{|V_{\text{cell}}|}P_M^{\text{avg}} - \gamma\frac{|M_{\text{cell}}|}{|V_{\text{cell}}|}P_{M+1}^{\min} - \beta_{M+1}P_{M+1}^{\min}. \tag{119}$$

For the steady state we thus obtain

$$\left(\gamma\frac{|M_{\text{cell}}|}{|V_{\text{cell}}|}+\beta_{M+1}\right)\bar{P}_{M+1}^{\min}\leq\alpha_{M+1}\frac{|M_{\text{cell}}|}{|V_{\text{cell}}|}\bar{P}_{M}^{\text{avg}}. \tag{120}$$

Rearranging gives the estimate

$$\bar{P}_{M+1}^{\min}\leq\frac{\alpha_{M+1}|M_{\text{cell}}|\bar{P}_{M}^{\text{avg}}}{\gamma|M_{\text{cell}}|+\beta_{M+1}|V_{\text{cell}}|}. \tag{121}$$

6.3 Upper bound for average concentration

In case of a spherical cell and a homogeneous signal on the cell surface, the maximum concentration is obtained on the membrane, which follows from the maximum principle. Therefore, we obtain the inequality

$$P_{M+1}^{\text{avg}}=\frac{1}{|V_{\text{cell}}|}\int\limits_{V_{\text{cell}}}P_{M+1}dV\leq\frac{1}{|M_{\text{cell}}|}\int\limits_{M_{\text{cell}}}P_{M+1}dA=P_{M+1}^{\max}. \tag{122}$$

Applying this inequality to the equation of mass conservation (104) yields

$$\frac{d}{dt}\int\limits_{V_{\text{cell}}}P_{M+1}dV\leq\int\limits_{M_{\text{cell}}}\alpha_{M+1}P_M-\gamma P_{M+1}^{\text{avg}}dA-\beta_{M+1}\int\limits_{V_{\text{cell}}}P_{M+1}dV. \tag{123}$$

Dividing by $|V_{\text{cell}}|$ and substition of P_M^{avg} and P_M^{avg} results in

$$\frac{d}{dt}P_{M+1}^{\text{avg}}\leq\alpha_{M+1}\frac{|M_{\text{cell}}|}{|V_{\text{cell}}|}P_{M}^{\text{avg}}-\gamma\frac{|M_{\text{cell}}|}{|V_{\text{cell}}|}P_{M+1}^{\text{avg}}-\beta_{M+1}P_{M+1}^{\text{avg}}. \tag{124}$$

For the steady state we thus obtain

$$\left(\gamma\frac{|M_{\text{cell}}|}{|V_{\text{cell}}|}+\beta_{M+1}\right)\bar{P}_{M+1}^{\text{avg}}\leq\alpha_{M+1}\frac{|M_{\text{cell}}|}{|V_{\text{cell}}|}\bar{P}_{M}^{\text{avg}}. \tag{125}$$

Rearranging gives the estimate

$$\bar{P}_{M+1}^{\text{avg}}\leq\frac{\alpha_{M+1}|M_{\text{cell}}|\bar{P}_{M}^{\text{avg}}}{\gamma|M_{\text{cell}}|+\beta_{M+1}|V_{\text{cell}}|}. \tag{126}$$

7 Estimates for concentration differences in the signaling cascade

We want to investigate the concentration difference and gradients that are generated in the cytosol in steady state. Therefore, we can multiply equation (5) for $M+1\leq n\leq N$ with $P_n-P_n^{\text{avg}}$, integrate over the domain V_{cell} and obtain

$$0=D_{\text{cyt}}\int\limits_{V_{\text{cell}}}\Delta\bar{P}_n(\bar{P}_n-\bar{P}_n^{\text{avg}})dV+\alpha_n\int\limits_{V_{\text{cell}}}\bar{P}_n(\bar{P}_n-\bar{P}_n^{\text{avg}})dV-\beta_n\int\limits_{V_{\text{cell}}}\bar{P}_n(\bar{P}_n-\bar{P}_n^{\text{avg}})dV. \tag{127}$$

Applying Green's theorem results in

$$D_{\text{cyt}}\int\limits_{V_{\text{cell}}}\nabla\bar{P}_n\cdot\nabla\bar{P}_ndV=\alpha_n\int\limits_{V_{\text{cell}}}\bar{P}_{n-1}(\bar{P}_n-\bar{P}_n^{\text{avg}})dV-\beta_n\int\limits_{V_{\text{cell}}}\bar{P}_n(\bar{P}_n-\bar{P}_n^{\text{avg}})dV. \tag{128}$$

Further applying Poincaré inequality yields

$$\frac{D_{\text{cyt}}}{C^2(V_{\text{cell}})}\int\limits_{V_{\text{cell}}}(\bar{P}_n-\bar{P}_n^{\text{avg}})^2dV\leq\alpha_n\int\limits_{V_{\text{cell}}}\bar{P}_{n-1}(\bar{P}_n-\bar{P}_n^{\text{avg}})dV-\beta_n\int\limits_{V_{\text{cell}}}\bar{P}_n(\bar{P}_n-\bar{P}_n^{\text{avg}})dV, \tag{129}$$

where C is the Poincaré constant that only depends on size and shape of the domain.
Since $\int\limits_{V_{\text{cell}}} (\bar{P}_n - \bar{P}_n^{\text{avg}})dV = 0$, we continue by adding a "zero", which results in

$$\frac{D_{\text{cyt}}}{C^2(V_{\text{cell}})}\|\bar{P}_n - \bar{P}_n^{\text{avg}}\|^2_{V_{\text{cell}},2} \leq \alpha_n \int\limits_{V_{\text{cell}}} (\bar{P}_{n-1} - \bar{P}_{n-1}^{\text{avg}})(\bar{P}_n - \bar{P}_n^{\text{avg}})dV - \beta_n \int\limits_{V_{\text{cell}}} (\bar{P}_n - \bar{P}_n^{\text{avg}})^2 dV. \quad (130)$$

We apply Hölder's inequality

$$(\frac{D_{\text{cyt}}}{C^2(V_{\text{cell}})} + \beta_n)\|\bar{P}_n - \bar{P}_n^{\text{avg}}\|^2_{V_{\text{cell}},2} \leq \alpha_n \|\bar{P}_{n-1} - \bar{P}_{n-1}^{\text{avg}}\|_{V_{\text{cell}},2}\|\bar{P}_n - \bar{P}_n^{\text{avg}}\|_{V_{\text{cell}},2}. \quad (131)$$

Further rearranging and employing the notation $\Sigma_n = \|\bar{P}_n - \bar{P}_n^{\text{avg}}\|_{V_{\text{cell}},2}$ results in

$$\begin{aligned}\Sigma_n = \|\bar{P}_n - \bar{P}_n^{\text{avg}}\|_{V_{\text{cell}},2} &\leq \frac{\alpha_n}{\frac{D_{\text{cyt}}}{C^2(V_{\text{cell}})} + \beta_n}\|\bar{P}_{n-1} - \bar{P}_{n-1}^{\text{avg}}\|_{V_{\text{cell}},2} \\ &= \frac{\alpha_n}{\frac{D_{\text{cyt}}}{C^2(V_{\text{cell}})} + \beta_n}\Sigma_{n-1}. \end{aligned} \quad (132)$$

Take the square of this inequality gives

$$\Sigma_n^2 \leq \frac{\alpha_n^2}{(\frac{D_{\text{cyt}}}{C^2(V_{\text{cell}})} + \beta_n)^2}\Sigma_{n-1}^2. \quad (133)$$

We now divide this inequality by $\bar{P}_n^{\text{avg}}|V_{\text{cell}}|$, which gives

$$\frac{\Sigma_n^2}{\bar{P}_n^{\text{avg}}|V_{\text{cell}}|} \leq \frac{\alpha_n^2}{(\frac{D_{\text{cyt}}}{C^2(V_{\text{cell}})} + \beta_n)^2}\frac{\Sigma_{n-1}^2}{\bar{P}_n^{\text{avg}}|V_{\text{cell}}|}. \quad (134)$$

Since $\bar{P}_n^{\text{avg}} = \frac{\alpha_n}{\beta_n}\bar{P}_{n-1}^{\text{avg}}$, we get

$$\frac{\Sigma_n^2}{\bar{P}_n^{\text{avg}}|V_{\text{cell}}|} \leq \frac{\alpha_n\beta_n}{(\frac{D_{\text{cyt}}}{C^2(V_{\text{cell}})} + \beta_n)^2}\frac{\Sigma_{n-1}^2}{\bar{P}_{n-1}^{\text{avg}}|V_{\text{cell}}|}. \quad (135)$$

Remark. The Poincaré constant scales linearliy with size, meaning that $C = C_s d$, where d is the diameter of the domain and C_s is the Poincaré constant for a domain with the same shape but a diameter of $d = 1$.

Proof. Assume we have an arbitrary cell shape in three space dimensions. We assume that the Poincaré inequality holds

$$\int\limits_{V_{\text{cell}}} (\bar{P}_n - \bar{P}_n^{\text{avg}})^2 dV \leq C^2 \int\limits_{V_{\text{cell}}} \nabla\bar{P}_n \cdot \nabla\bar{P}_n dV, \quad (136)$$

for all $P_n \in W^{1,2}(V_{\text{cell}})$ for a constant $C > 0$. We introduce the linear transformation $T : (x_1, x_2, x_3) \to (\xi x_1, \xi x_2, \xi x_3)$ with a scaling factor $\xi > 0$. Furthermore we define $\tilde{V}_{\text{cell}} = \{T\mathbf{x}|\mathbf{x} \in V_{\text{cell}}\}$ and $\tilde{P}_n(T\mathbf{x}) := P_n(\mathbf{x})$. In this case, we obtain $\tilde{\nabla}\tilde{P}_n = \frac{1}{\xi}\nabla P_n$. Setting $\tilde{C} = \xi C$ results in

$$\begin{aligned}\int\limits_{\tilde{V}_{\text{cell}}} (\tilde{P}_n - \tilde{P}_n^{\text{avg}})^2 d\tilde{V} &= \int\limits_{V_{\text{cell}}} (\bar{P}_n - \bar{P}_n^{\text{avg}})^2 \xi^3 dV \\ &\leq C^2\xi^3 \int\limits_{V_{\text{cell}}} \nabla\bar{P}_n \cdot \nabla\bar{P}_n dV \\ &= \tilde{C}^2 \int\limits_{V_{\text{cell}}} \frac{1}{\xi}\nabla\bar{P}_n \cdot \frac{1}{\xi}\nabla\bar{P}_n \xi^3 dV \\ &= \tilde{C}^2 \int\limits_{\tilde{V}_{\text{cell}}} \nabla\tilde{P}_n \cdot \nabla\tilde{P}_n d\tilde{V},\end{aligned}$$

which is the Poincaré inequality for $\tilde{V}_{\text{cell}}$. For $\xi = 1/d$ the domain $\tilde{V}_{\text{cell}}$ has a $\text{diam}(\tilde{V}_{\text{cell}}) = 1$. Therefore, $C_s := C/d$ is the sought-after constant. □

8 Time scaling

Using the method from [3], we can calculate the time scaling exactly. In case of the (MMC) cascade and a homogeneous signal the Laplace transform is given by

$$sF_1(s) = \frac{\alpha_1 \bar{P}_0}{s} - \beta_1 F_1(s) \qquad \text{on the membrane,} \tag{137}$$

$$sF_2(s) = \alpha_2 F_1(s) - \beta_2 F_2(s) \qquad \text{on the membrane,} \tag{138}$$

$$sF_3(r,s) = D_{\text{cyt}} \Delta F_3(r,s) - \beta_3 F_3(r,s) \qquad \text{in the cytosol,} \tag{139}$$

$$D_{\text{cyt}} \frac{\partial F_3(r,s)}{\partial r} = \alpha_3 F_2(s) - \gamma F_3(r,s) \qquad \text{on the membrane,} \tag{140}$$

$$-D_{\text{cyt}} \frac{\partial F_3(r,s)}{\partial r} = -\epsilon F_3(r,s) \qquad \text{at the nucleus.} \tag{141}$$

Note, that F_1, F_2 and F_3 are independent of ϕ and θ, since we assume that the signal is constant in space and time $P_0(\phi,\theta,t) \equiv P_0$ and the initial conditions are $P_1(\phi,\theta,0) = P_2(\phi,\theta,0) \equiv 0$ as well as $P_3(\phi,\theta,0) \equiv 0$. For a fixed $s > 0$ and $R_{\text{nuc}} = 0$ an explicit solution of the system is given by

$$\begin{aligned}
F_1(s) &= \frac{\alpha_1 \bar{P}_0}{s(\beta_1+s)}, \\
F_2(s) &= \frac{\alpha_1 \bar{P}_0}{s(\beta_1+s)} \frac{\alpha_2}{(\beta_2+s)}, \\
F_3(s) &= \frac{\alpha_1 \bar{P}_0}{s(\beta_1+s)} \frac{\alpha_2}{(\beta_2+s)} \frac{\alpha_3 P_2}{\sqrt{D_{\text{cyt}}(\beta_3+s)}\, i_1\left(R_{\text{cell}}\sqrt{\frac{\beta_3+s}{D_{\text{cyt}}}}\right) + \gamma i_0\left(R_{\text{cell}}\sqrt{\frac{(\beta_3+s)}{D_{\text{cyt}}}}\right)} i_0\left(r\sqrt{\frac{\beta_3+s}{D_{\text{cyt}}}}\right) \\
&= \frac{\alpha_1 \bar{P}_0}{s(\beta_1+s)} \frac{\alpha_2}{(\beta_2+s)} \frac{\alpha_3 P_2 \mathcal{Z}(s)}{\mathcal{N}(s)}.
\end{aligned}$$

We define the following functions

$$\begin{aligned}
R_1(s) &= \frac{1}{s} - \frac{F_1(s)}{\bar{P}_1(r)}, \\
R_2(s) &= \frac{1}{s} - \frac{F_2(s)}{\bar{P}_2(r)}, \\
R_3(r,s) &= \frac{1}{s} - \frac{F_3(r,s)}{\bar{P}_3(r)}.
\end{aligned}$$

From [4] we know that the local accumulation time is given by

$$\tau_1 = \lim_{s\to 0} R_1(s) = \frac{1}{\beta_1}, \tag{142}$$

$$\tau_2 = \lim_{s\to 0} R_2(s) = \frac{1}{\beta_1} + \frac{1}{\beta_2}. \tag{143}$$

For $\tau_3 = \lim\limits_{s\to 0} R_3(r,s)$ can be calculated as follows:

$$\begin{aligned}
\lim_{s\to 0} R_3(r,s) &= \lim_{s\to 0}\left[\frac{1}{s} - \frac{\beta_1}{s(\beta_1+s)} \frac{\beta_2}{(\beta_2+s)} \frac{\mathcal{Z}(s)\mathcal{N}(0)}{\mathcal{Z}(0)\mathcal{N}(s)}\right] \\
&= \lim_{s\to 0}\left[\frac{(\beta_1+s)(\beta_2+s)\mathcal{Z}(0)\mathcal{N}(s) - \beta_1\beta_2\mathcal{Z}(s)\mathcal{N}(0)}{s(\beta_1+s)(\beta_2+s)\mathcal{Z}(0)\mathcal{N}(s)}\right] \\
&= \lim_{s\to 0}\left[\frac{(\beta_2+s)\mathcal{Z}(0)\mathcal{N}(s) + (\beta_1+s)\mathcal{Z}(0)\mathcal{N}(s) + (\beta_1+s)(\beta_2+s)\mathcal{Z}(0)\mathcal{N}'(s) - \beta_1\beta_2\mathcal{Z}'(s)\mathcal{N}(0)}{(\beta_1+s)(\beta_2+s)\mathcal{Z}(0)\mathcal{N}(s) + s(\beta_2+s)\mathcal{Z}(0)\mathcal{N}(s) + s(\beta_1+s)\mathcal{Z}(0)\mathcal{N}(s) + s(\beta_1+s)(\beta_2+s)\mathcal{Z}(0)\mathcal{N}'(s)}\right] \\
&= \left[\frac{\beta_2\mathcal{Z}(0)\mathcal{N}(0) + \beta_1\mathcal{Z}(0)\mathcal{N}(0) + \beta_1\beta_2\mathcal{Z}(0)\mathcal{N}'(0) - \beta_1\beta_2\mathcal{Z}'(0)\mathcal{N}(0)}{\beta_1\beta_2\mathcal{Z}(0)\mathcal{N}(0) + 0}\right] \\
&= \frac{1}{\beta_1} + \frac{1}{\beta_2} + \underbrace{\frac{\mathcal{Z}(0)\mathcal{N}'(0) - \mathcal{Z}'(0)\mathcal{N}(0)}{\mathcal{Z}(0)\mathcal{N}(0)}}_{=\mathcal{T}}.
\end{aligned}$$

In the case of $R_{\text{nuc}} = 0$ an explicit form of $\mathcal{T}$ was derived in [5]. The analytical solution of τ_3 is plotted for a range of parameters in Fig A2.

References

[1] Milton Abramowitz and Irene A Stegun. *Handbook of mathematical functions: with formulas, graphs, and mathematical tables*, volume 55. Courier Corporation, 1964.

[2] Martin S. Alnæs, Jan Blechta, Johan Hake, August Johansson, Benjamin Kehlet, Anders Logg, Chris Richardson, Johannes Ring, Marie E. Rognes, and Garth N. Wells. The FEniCS Project Version 1.5. *Archive of Numerical Software*, 3(100), 2015.

[3] Alexander M Berezhkovskii, Christine Sample, and Stanislav Y Shvartsman. How long does it take to establish a morphogen gradient? *Biophysical journal*, 99(8):L59–L61, 2010.

[4] Alexander M Berezhkovskii, Christine Sample, and Stanislav Y Shvartsman. Formation of morphogen gradients: Local accumulation time. *Physical Review E*, 83(5):051906, 2011.

[5] Adam J Ellery, Matthew J Simpson, Scott W McCue, and Ruth E Baker. Simplified approach for calculating moments of action for linear reaction-diffusion equations. *Physical Review E*, 88(5):054102, 2013.

[6] David Gilbarg and Neil S Trudinger. *Elliptic partial differential equations of second order*. Springer, 2015.

[7] Reinhart Heinrich, Benjamin G Neel, and Tom A Rapoport. Mathematical models of protein kinase signal transduction. *Molecular cell*, 9(5):957–970, 2002.

[8] Anders Logg, Kent-Andre Mardal, Garth N. Wells, et al. *Automated Solution of Differential Equations by the Finite Element Method*. Springer, 2012.

[9] Elias M Stein and Guido L Weiss. *Introduction to Fourier Analysis on Euclidean Spaces*, volume 1. Princeton University Press, 1971.

9 SI Figures

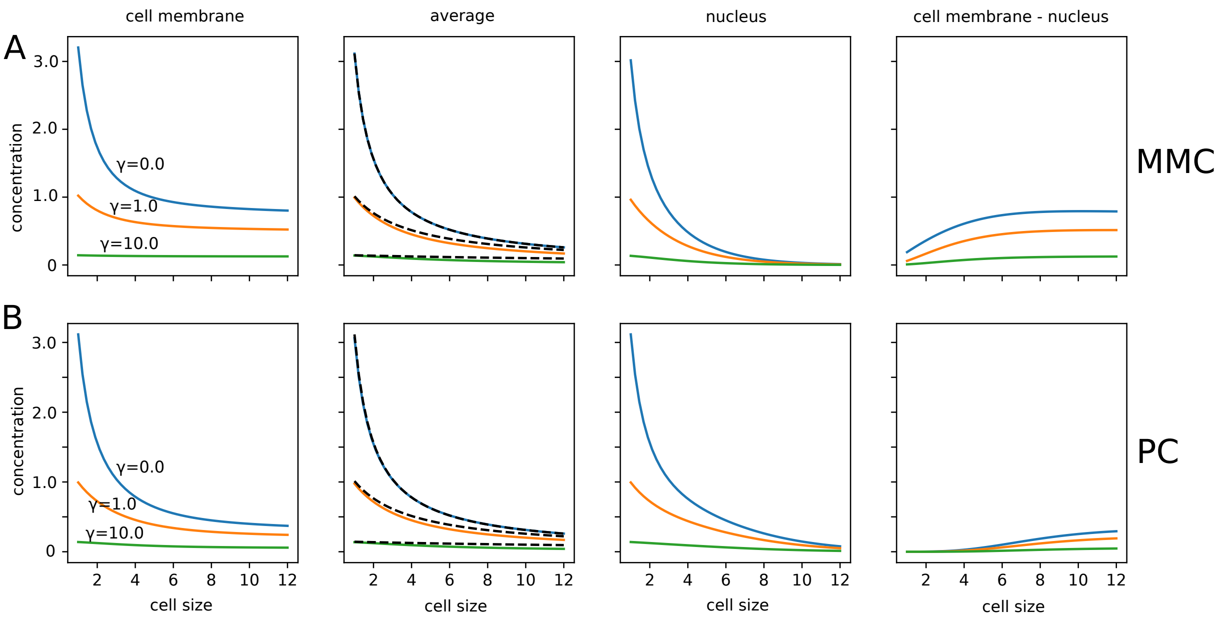

Fig A 1: **Dependence of the concentration on cell size.** The concentration of the third cascade element at the cell-membrane, the nucleus as well as the concentration difference from cell-membrane to cell nucleus was plotted. The phosphorylation rates and phosphatase rates were fixed at $\alpha_1 = \alpha_2 = \alpha_3 = 1.5$ and $\beta_1 = \beta_2 = \beta_3 = 1.5$. **A:** A MMC cascade with two membrane-bound components and only one cytosolic species is shown. **B:** In contrast the PC cascade, where all three components diffuse freely in the cytosol exhibits much shallower gradients. For both spatial motifs size dependence of the concentration level decreases with higher values of γ.

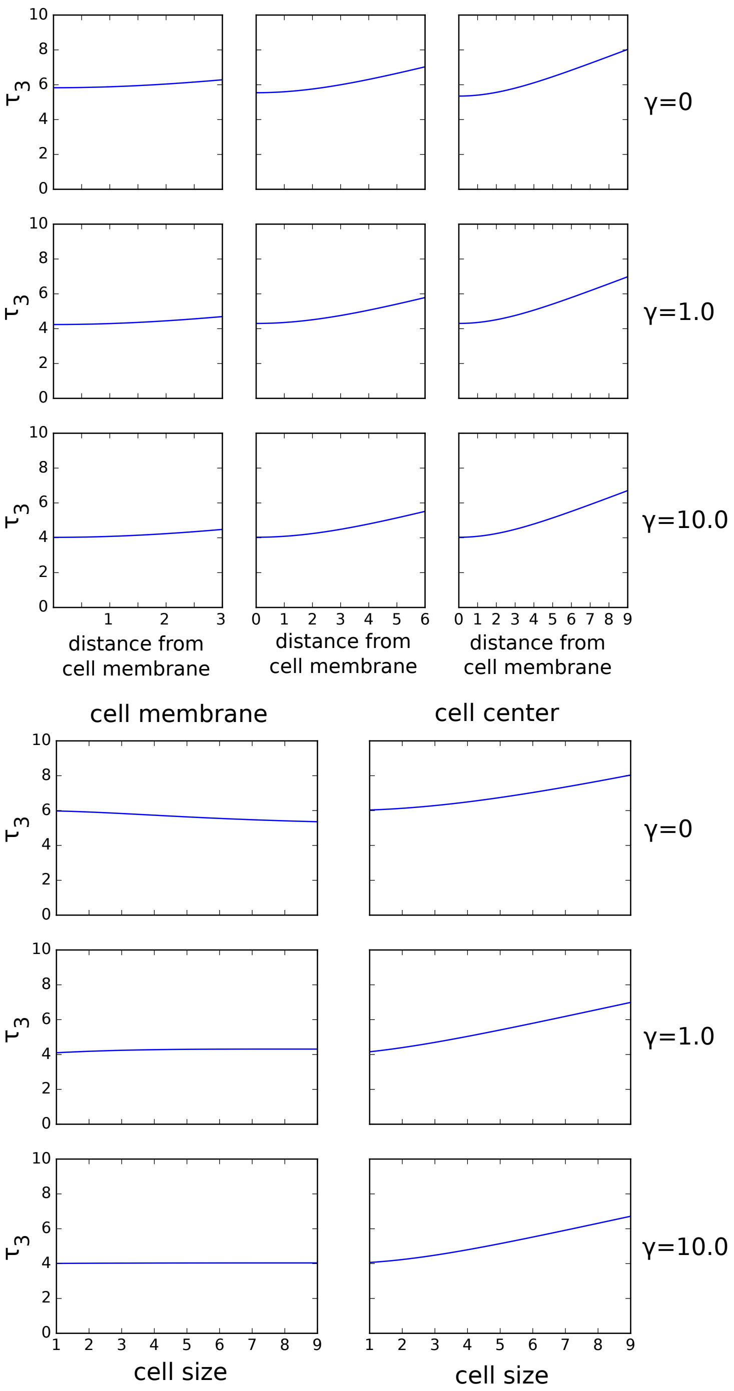

Fig A 2: **Accumulation time scales.** Plot of the accumulation time τ_3 of P_3 for the mixed membrane-cytosolic model (MMC). The parameters used were $\alpha_1, \alpha_2, \alpha_3 = 1$ and $\beta_1, \beta_2, \beta_3 = 0.5$.

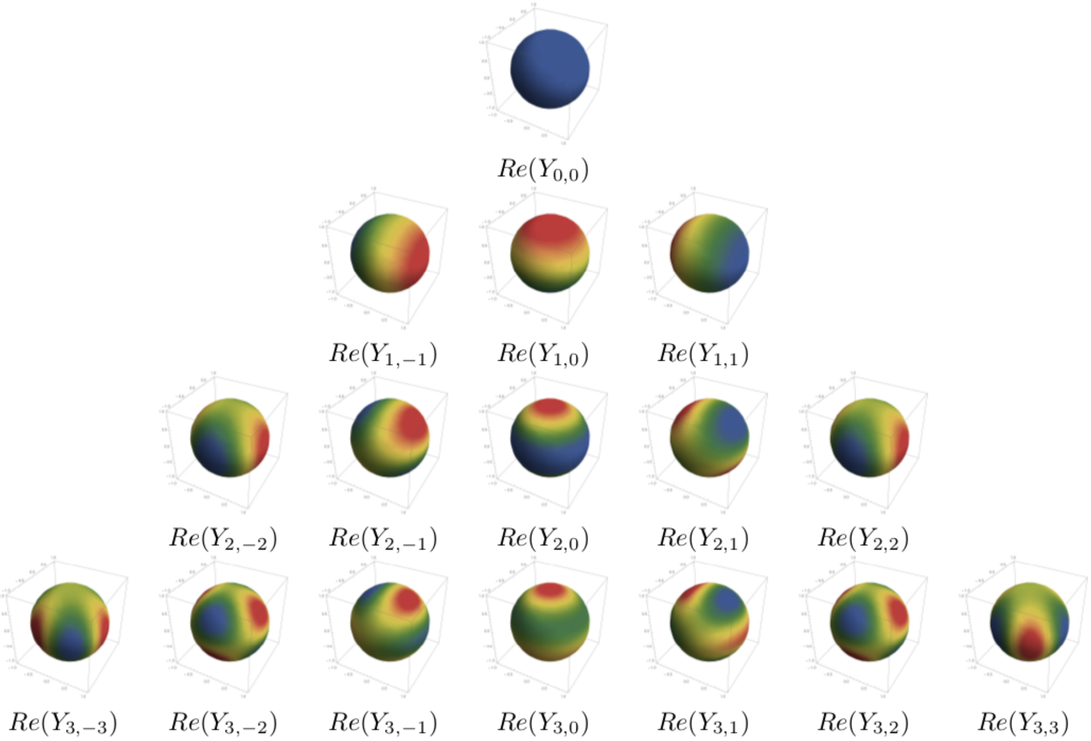

Fig A 3: **Illustration of the spherical harmonics.**
